# Fine Mapping and Functional Research of Key Genes for Photoperiod Sensitivity in Maize

**DOI:** 10.3389/fpls.2022.890780

**Published:** 2022-07-12

**Authors:** Jianbo Fei, Qingping Jiang, Mingyang Guo, Jianyu Lu, Piwu Wang, Siyan Liu, Jing Qu, Yiyong Ma, Shuyan Guan

**Affiliations:** ^1^College of Bioscience, Jilin Agricultural University, Changchun, China; ^2^Joint Laboratory of International Cooperation in Modern Agricultural Technology of Ministry of Education, Jilin Agricultural University, Changchun, China; ^3^College of Agriculture, Jilin Agricultural University, Changchun, China

**Keywords:** maize, quantitative trait loci, photoperiod sensitivity, transcriptome, *ZmPRR9.3*, *ZmPRR3-a*, *ZmTOC1b*

## Abstract

Maize is native to the tropics and is very sensitive to photoperiod. Planting in temperate regions with increased hours of daylight always leads to late flowering, sterility, leggy plants, and increased numbers of maize leaves. This phenomenon severely affects the utilization of tropical maize germplasm resources. The sensitivity to photoperiod is mainly reflected in differences in plant height (PH), ear height (EH), total leaf number (LN), leaf number under ear (LE), silking stage (SS), and anthesis stage (AT) in the same variety under different photoperiod conditions. These differences are more pronounced for varieties that are more sensitive to photoperiod. In the current study, a high-density genetic map was constructed from a recombinant inbred line (RIL) population containing 209 lines to map the quantitative trait loci (QTL) for photoperiod sensitivity of PH, EH, LN, LE, SS, and AT. A total of 39 QTL were identified, including three consistent major QTL. We identified candidate genes in the consensus major QTL region by combined analysis of transcriptome data, and after enrichment by GO and KEGG, we identified a total of four genes (Zm00001d006212, Zm00001d017241, Zm00001d047761, and Zm00001d047632) enriched in the plant circadian rhythm pathway (KEGG:04712). We analyzed the expression levels of these four genes, and the analysis results showed that there were significant differences in response under different photoperiod conditions for three of them (Zm00001d047761, Zm00001d006212 and Zm00001d017241). The results of functional verification showed that the expression patterns of genes rhythmically oscillated, which can affect the length of the hypocotyl and the development of the shoot apical meristem. We also found that the phenotypes of the positive plants were significantly different from the control plants when they overexpressed the objective gene or when it was knocked out, and the expression period, phase, and amplitude of the target gene also shifted. The objective gene changed its own rhythmic oscillation period, phase, and amplitude with the change in the photoperiod, thereby regulating the photoperiod sensitivity of maize. These results deepen our understanding of the genetic structure of photoperiod sensitivity and lay a foundation for further exploration of the regulatory mechanism of photoperiod sensitivity.

## Introduction

Maize is native to Mexico. It is a typical short-day plant that is sensitive to sunlight for 12–13 h (Ellis et al., 1992a; Bonhomme et al., 1994). However, the world’s three major golden maize belts are all located at N40–42° latitude, and the average sunshine duration in summer at this latitude is more than 14 h, which results in late flowering, sterile, and leggy maize varieties (Struik et al., 1986; Fedorov, 1987; Muchow and Carberry, 1989). These factors severely affect the yield of maize and the utilization of germplasm resources. This phenomenon is mainly caused by the different sensitivity of maize to photoperiod (Francis et al., 1969; Francis and Grogan, 1972). The photoperiod-sensitive varieties are significantly affected by different durations of sunshine, while the photoperiod-insensitive varieties are less affected (Ellis et al., 1992b). Kiniry et al. (1983) reported that maize was insensitive to photoperiod changes from 4 to 8 days (morphologically 35–50% visible leaf stage) before tassel differentiation, and the tassel differentiation stage was a sensitive stage for maize to respond to the photoperiod. Rood and Major (1980) proposed for the first time that the photoperiod sensitivity index was the number of growth days delayed by each 1 h extension of the photoperiod. Bonhomme et al. (1994) proposed an indicator that combines light duration and temperature to measure the light sensitivity of maize varieties. They regarded the average heat unit with less than 13 h of light in the interval from sowing to tasseling as the basic heat unit, and used the regression value of the heat unit and the basic heat unit when the cultivar exceeded a 13 h photoperiod as a photoperiod-sensitive indicator.

Gouesnard et al. (2002) calculated the accumulated temperature from sowing to tassel flowering with reference to Bonhomme’s method, and used the regression slope of accumulated temperature and photoperiod as the photoperiod sensitivity value of the population. Research has also been performed using RD to estimate the photoperiod sensitivity index: RD (%) = [(L-S)/S] × 100 (Moutiq et al., 2002). In the formula, L represents the average value of a certain property of a material in a long-day (LD), and S represents the average value of a certain property of a material in a short-day (SD). This method is widely used because it can comprehensively be used to measure photoperiod sensitivity for different traits. Previous studies (Ellis et al., 1992b; Goodman, 1992; Koester et al., 1993) have shown that the maize anthesis period, plant height, ear height, interval of silking and anthesis, number of male flower branches, leaf area at ear position, number of leaves under ear position, total number of leaves, and other traits are closely related to photoperiod sensitivity.

Although photoperiod sensitivity is an important trait, its genetic mechanisms are largely unknown. There are few studies on quantitative trait loci (QTL) mapping of maize photoperiod sensitivity. Jin et al. (2018) linked 53 CCT family genes related to flowering in maize based on genome-wide-association studies (GWAS), and verified that the *ZmCOL3* gene changes the plant flowering time by interfering with plant endogenous rhythms. Through QTL mapping and transcriptome analysis under LD light, Ku et al. (2016) determined that the *ZmCCT* gene not only corresponds to changes in photoperiod but it also enhances maize resistance to head blight and drought. Hung et al. (2012) collected maize inbred lines from different latitudes around the world to construct natural populations, and identified two major genes, *ZmCCT9* and *ZmCCT10*, through GWAS association analysis of photoperiod sensitivity, and knocked them out using CRISPR-Cas9 technology, verifying their effect on maize flowering.

The most important node in the life cycle of higher plants is the transition from vegetative growth to reproductive growth (Ha et al., 2010). During this period, the shoot apical meristem (SAM) no longer forms leaf primordia and axillary bud primordia, but forms flower primordia or floret primordia (Teichmann and Muhr, 2015). Studies have shown that before SAM differentiation produces morphological changes, there is a critical period that is very sensitive to external photoperiod changes, and the pathway corresponding to this period is the photoperiod pathway (Taoka et al., 2011, 2013). This pathway includes the input, core oscillator, and output pathways of the optical signal.

In Arabidopsis, the input pathways are photoreceptors, including phytochromes and cryptochromes, which transmit light signals to core oscillators by sensing different spectra of light (Harmer, 2009). The composition of the core oscillator is relatively complex, consisting of a transcription–translation feedback inhibition loop composed of a morning loop, a night loop, and a central loop (Alabadi et al., 2001; Gendron et al., 2012; Huang et al., 2012). *CCA1/LHY* and *TOC1* together constitute the central loop. *CCA1* and *LHY* are expressed in the morning and together suppress the expression of *TOC1*. The expression of *CCA1* and *LHY* was inhibited at night, and the expression of *TOC1* reached a peak (Alabadi et al., 2001). *CCA1/LHY* also formed a morning loop with *PRR9/PRR7*, and *PRR9/7* generally reached peak expression in the morning, inhibiting the expression of *CCA1* and *LHY* at the transcriptional level (Nakamichi et al., 2010). Conversely, the expression of *CCA1* and *LHY* also inhibited the expression of *PRR9/7* (Wang et al., 2013). The night loop mainly relies on the EC complex to function (Nusinow et al., 2011). The EC complex is composed of nuclear proteins *ELF3*, *ELF4*, and *LUX*, which can regulate each other with *CCA1/LHY* and indirectly promote the expression of *CCA1/LHY* (Adams et al., 2015).

The core oscillator integrates the signals transmitted by the input pathway through the communication between the morning loop, the night loop, and the central loop, and transmits the rhythm signal to the output pathway while regulating its own rhythm (Hayama et al., 2017). The *GI* integrates and activates the expression of the *CO* gene, thereby regulating the downstream flowering gene, *FT* (Tripathi et al., 2017). The *FT* gene is transported to the SAM *via* vascular bundles, thereby activating or inhibiting the differentiation and development of floral organs (Tripathi et al., 2017). Studies have shown that although rice crops are short-day plants, the *OSMADS50, Hdl(sel), Hd3a,* and *OsGI* genes in rice are orthologs of Arabidopsis *SOC1, CO, FT*, and *GI*, respectively, and the photoperiod input pathway of rice is similar to that of Arabidopsis (Zhao et al., 2012; Yang et al., 2013; Atamian and Harmer, 2016; Sakuraba et al., 2016). In addition to rice, orthologs of Arabidopsis photoperiod-regulated genes have also been found in barley, morning glory, wheat, and other plants (Koo et al., 2013; Boden et al., 2014; Atamian and Harmer, 2016; Ford et al., 2016). However, some genes respond differently to the environment as compared to Arabidopsis, indicating that the photoperiod regulatory pathway is relatively conserved in different plants but not identical (Meng et al., 2011; Lu et al., 2017; Yue et al., 2017).

In maize, the mechanism that regulates photoperiod remains unclear, and it is necessary for photoperiod-related genes to be explored and verified. In the current study, we identified QTL that were associated with photoperiod sensitivity using a recombinant inbred line (RIL) population constructed from the photoperiod-sensitive variety Q102 and photoperiod-insensitive variety S122, whereby phenotypic data was collected, and high-throughput sequencing was performed. We also used transcriptome data and candidate genes in the consistent main effect QTL region for joint analysis, finally identified objective genes through qPCR analysis, and then verified their functions.

## Materials and Methods

### Plant Materials

**Figure 1:**
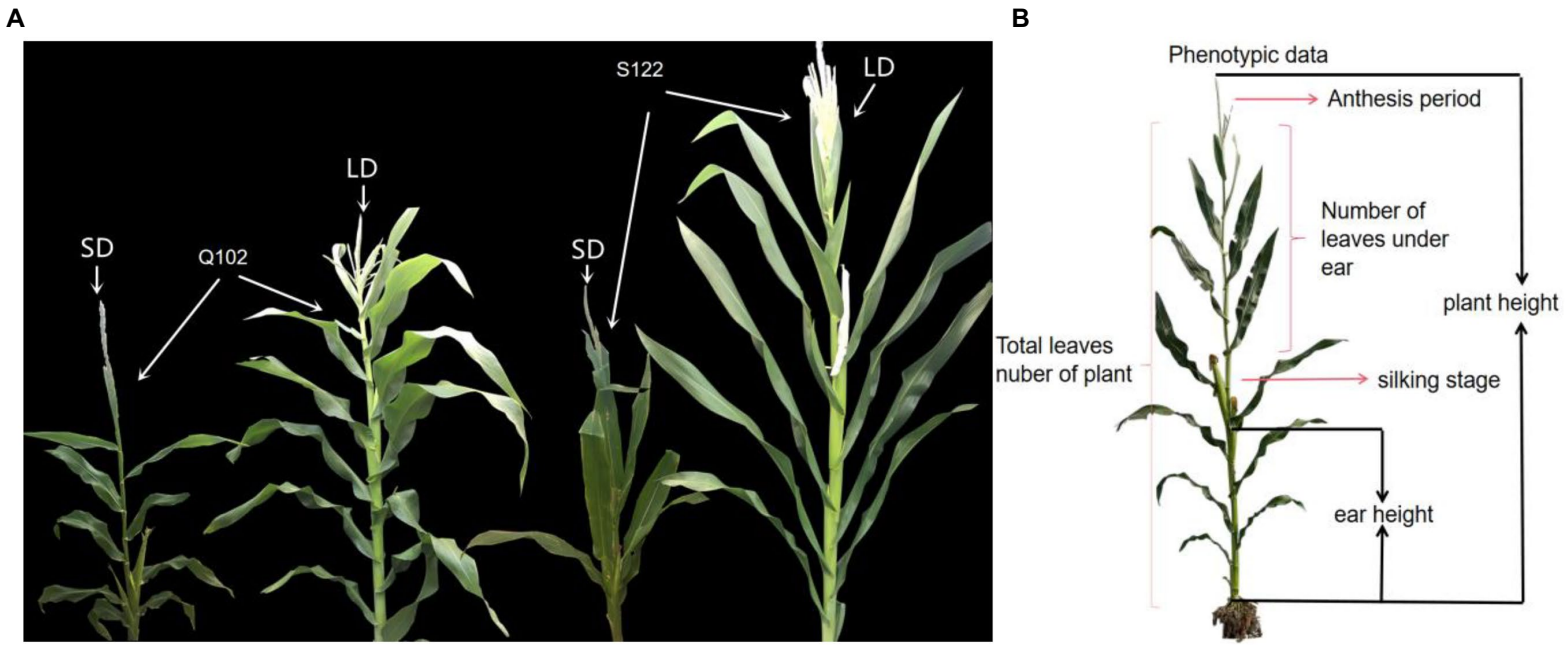
Photoperiod-sensitive phenotypes in maize plants. **(A)** Phenotypes of RIL population parents under LD (15/9 h, light/dark) (Fei et al., 2022) and SD (9/15 h, light/dark) conditions. **(B)** Schematic diagram of the measurement positions of photoperiod-sensitive phenotypes.

The maize inbred lines S122 (male parent; LD photoperiod-sensitive) and Q102 (female parent; LD photoperiod-insensitive; HZ4 sister line) were acquired from the Huadian Qiufeng Agricultural Research Institute, and were subsequently selected to construct a population of RILs including 209 lines by single seed propagation (Figure 1). The RIL mapping population was planted at three sites (Hainan Sanya N: 18°09′/E: 108°56′; Jilin Changchun N: 43°05′/E: 124°18′) and Gongzhuling (N: 43°31′/E: 124°49′) for two consecutive years (2018 and 2019). At each location, there was a randomized complete block design with three replications for the field experiment. The study protocol complied with relevant institutional, national, and international guidelines and legislation.

HZ4 (photoperiod-insensitive) and H496 (photoperiod-sensitive) plant material was used for transcriptome sequencing. H496 is a near-isogenic line (NIL) of HZ4, obtained by crossing HZ4 (recurrent parent) and CML288 (donor parent). HZ4 is from the typical Chinese heterotic group of Tangsipingtou, while CML288 is a tropical inbred line variety from the International Maize and Wheat Improvement Center in Mexico. The newly developed 4th and 5th leaves and SAMs were collected at the same time under LD light (15/9 h, light/dark), with three biological replicates per group of samples. HZ4 and H496 plant materials, Sample acquisition, storage, and sampling methods for transcriptomics analyses were have published in previously research by Ku et al. (2016).

The maize inbred line Dan988s (LD photoperiod-sensitive) is the recipient material for genetic transformation. It is an improved variety of Dan988, with a high callus induction rate and genetic transformation efficiency, and was provided by the Plant Biotechnology Center of Jilin Agricultural University.

### Phenotype Collection

The phenotypic data affected by maize photoperiod sensitivity can be measured: plant height (PH), ear height (EH), total leaves number for the plant (LN), number of leaves under the ear (LE), silking stage (SS), and anthesis stage (AT). Plant height is measured from the ground to the tip of the primary inflorescence, while ear height is measured from the ground to the node of the primary ear. The total number of leaves on the plant includes all the leaves from the first leaf near the root to the last leaf near the tassel. The number of leaves under a plant ear includes all leaves from the first leaf near the root to the last leaf near the ear. The silking stage includes the number of days from the emergence of the seedling to the silking of the ear. The anthesis stage is the number of days from the emergence of the seedling to the anthesis of the tassel (Figure 1). The photoperiod sensitivity was calculated based on the phenotypes under LD light (Jilin Changchun N: 43°05′/E: 124°18′ and Gongzhuling N: 43°31′/E: 124°49′) and SD light (Hainan Sanya N: 18°09′/E: 108°56′), and the calculation method was based on previous studies.

RD = [(L−S)/S × 100]

In the formula, RD denotes the photoperiod sensitivity, L denotes the phenotypic value under LD light, and S denotes the phenotypic value under SD light. The phenotypic values measured under long-day and short-day are brought into the formula to finally obtain the plant height photoperiod sensitivity (PHPS), ear height photoperiod sensitivity (EHPS), plant total leaves number photoperiod sensitivity (LNPS), under ear leaves number photoperiod sensitivity (LEPS), silking stage photoperiod sensitivity (SSPS), and anthesis stage photoperiod sensitivity (ATPS).

### DNA Extraction and Genotyping

DNA extraction was performed using cetyl trimethylammonium bromide (CTAB; Mu et al., 2010). Genotyping was performed by specific locus amplified fragment sequencing (SLAF-seq), and SLAF tags were designed based on the Zm-B73-REFERENCE-GRAMENE-4.0 reference genome. The *Hea*III and *Hpy*166II double-enzyme digestion scheme designed by bioinformatics software was used, and the average length of the tags was 414–464 bp. PolyA was added to the 3′ segment of the digested fragment, which was then amplified and purified, and the purified product was subsequently sequenced on the Illumina HiSeq 2,500 paired-end sequencing platform. Low-quality sequences were removed, and Burrows–Wheeler alignment (BWA) software was used to map the remaining sequences to the reference genome. Sequences with a similarity greater than 95% were considered the same SLAFs. All the SLAF markers that were consistent in parents and offspring were genotyped.

### Genetic Map Construction and QTL Mapping

In this study, the genetic map of the RIL population was constructed using ICIMapping 4.2 software. A limit of detection (LOD) threshold of 2.5 was used to assign markers to the same linkage group. All the above marks must meet the following requirements: (1) Filter the parental sequencing depths to less than 10-fold. Genotyping of progeny according to parents, high-depth parental sequencing depth ensures the correctness of progeny typing. (2) Offspring typing correction. If the SNP depth of the progeny is less than 2-fold, the depth is too low and is corrected as missing. (3) Integrity rung filtering. Determine the genotypes that cover at least the markers of more than 75% of all progeny individuals. That is, for a single polymorphic marker site, there should be at least 75 individuals in 100 offspring with a definite genotype. (4) Partial separation marker filtering. Partial segregation markers are ubiquitous and generally do not affect the construction of maps, but may have an impact on QTL positioning. The polymorphism markers with severe partial separation (chi-square test *p* < 0.01) were filtered by the partial separation marker processing method for reference. The observed frequencies at each marker were tested against the expected Mendelian segregation ratio of 1:2:1 using a chi-square test for goodness of fit. Using the complete interval mapping method in ICIMapping 4.2, QTL mapping was performed on the RIL mapping population. The QTL positioning results are displayed by circos plot (Yiming et al., 2018) made by shinycircos online software.^1^

### Transcriptome Data Analysis and Candidate Gene Identification

Transcriptome data come from the National Center for Biotechnology Information (NCBI; accession # SRP072496; BioProject ID PRJNA316482). Data quality control was performed by fastq. According to the quality control results, data filtering was performed using Sickle software to remove low-quality sequences and ensure paired-end symmetry.

TopHat2 software was used for transcriptome-genome alignment, and BowTie2 software was used to build an index with Zm-B73-REFERENCE-V4 as the reference genome before alignment. The comparison results were counted by ht-seq, and the count value of the different experimental groups was finally output. The significance of gene expression differences between different experimental groups was calculated by DEseq. Significantly different genes (*p* ≤ 0.05) were compared with genes in the consistent QTL region to identify candidate genes. Gene ontology (GO) was used for the functional annotation of candidate genes, and Kyoto Encyclopedia of Genes and Genomes (KEGG) was used for the annotation of involved regulatory pathways. Finally, candidate genes related to photoperiod regulation were identified through the annotation results.

### RNA Extraction and Real-Time PCR

To verify the function of the candidate genes, the expression levels of the candidate genes were analyzed using the parents cultured to the 5-leaf stage under LD (15/9 h, light/dark) and SD (9/15 h, light/dark) conditions. The total RNA was isolated from 100 mg plant tissues following a TRIzol method, while the quantity and quality of RNA was determined using a NanoDrop spectrophotometer.

The cDNA reverse transcript was produced from RNA using a Thermo cDNA kit. qRT-PCR primers were designed using IDT online software, the internal reference gene was Actin II, and the primers were synthesized by Kumei Biological Co., Ltd. The reactions were completed in a 25-μL total volume using the SYBR Green PCR Master Mix kit and a Light Cycler® 480II Sequence Detection System. The relative expression levels of candidate genes were calculated by comparison using the 2^-∆∆ct^ method. The qRT-PCR and data analysis were performed using methods described by Chen et al. (2004).

### Bioinformatics Analysis of Object Genes

MEGA7.0 software^2^ was used to align the PRR family proteins of maize, rice, and Arabidopsis to construct a phylogenetic tree. This tree was based on the Jones Taylor Thornton model and 1,000 bootstrap replicates using the neighbor-joining (NJ) method, and protein sequence information for all genes was obtained from the NCBI. All protein secondary domain analyses of the PRR family were performed using pfam online software.^3^ DNAman software^4^ is used for the sequence alignment of the coding region and promoter region of the target gene. The online software plantCARE^5^ is used to analyze the cis-acting elements in the promoter region of the object gene.

### Vector Construction and Plant Transformation

The cloning vector was pMD-18 T, which was purchased from TaKaRa Co., Ltd. The plant expression vector used was pCambia3301, the prokaryotic expression vector was PET-22b, and DH5α *Escherichia coli* strain and Agrobacterium EHA105 strain were all provided by the Plant Biotechnology Center of Jilin Agricultural University. The expression vector connection method adopted seamless cloning technology, and the seamless cloning kit was purchased from Biyuntian Biotechnology Co., Ltd.

The CRISPR/Cas9 Vector Construction Kit was purchased from Hangzhou Baige Biotechnology Co., Ltd. The construction of the CRISPR/Cas9 vector and target design were implemented according to Wu et al. (2020). All primers were designed by Primer Premier 5.0^6^ software (Supplementary Table S7). The genetic transformation of maize is shown in Supplementary Figure S8 with reference to previous studies (Moutiq et al., 2002).

### Phenotypic Identification of Transgenic Materials

We analyzed the growth and development of SAMs in transgenic plants and controls under LD light (15/9 h, light/dark). The SAMs at five stages (23, 27, 30, 33, 36 days) after emergence were selected and dissected under an optical microscope, washed with 70% ethanol, and thinly sliced. The morphology was observed and recorded under an electron microscope. In addition, we also measured the hypocotyls of maize. The seeds were transferred to LD light (15/9 h, light/dark) for 5 days after germination in the dark, and the hypocotyl length was measured with a vernier caliper. Other phenotypic (PH, EH, LN, LE, SS, AT) data collection for transgenic and control plants was the same as before.

### Data Statistics and Analysis

The data involved in this study consist of three independent replicates expressed as the mean ± standard error. Descriptive statistical analysis, ANOVA, frequency distribution analyses, correlation analysis, and heritability calculations were all performed by SPSS v25 software. The broad-sense heritability was calculated according to previous method (Mandozai et al., 2021):


$$H^{2}=\delta^{2}{}_{G}/\delta^{2}{}_{P},\delta^{2}{}_{G}=\left(\mathrm{MSG}-\mathrm{MSE}/\mathrm{rep}\right)$$

$$\delta^{2}{}_{P}=\left(\mathrm{MSG}-\mathrm{MSE}/\mathrm{rep}\right)+\mathrm{MSE}$$

In the formula, δ^2^_G_ denotes the variance of the genotype, δ^2^_P_ denotes the variance of the phenotype, MSE denotes the mean square error, MSG denotes the mean square genotype, and rep (rep = 3) indicates the number of repetitions for each experiment.

## Results

### Phenotypic Analysis

**Figure 2:**
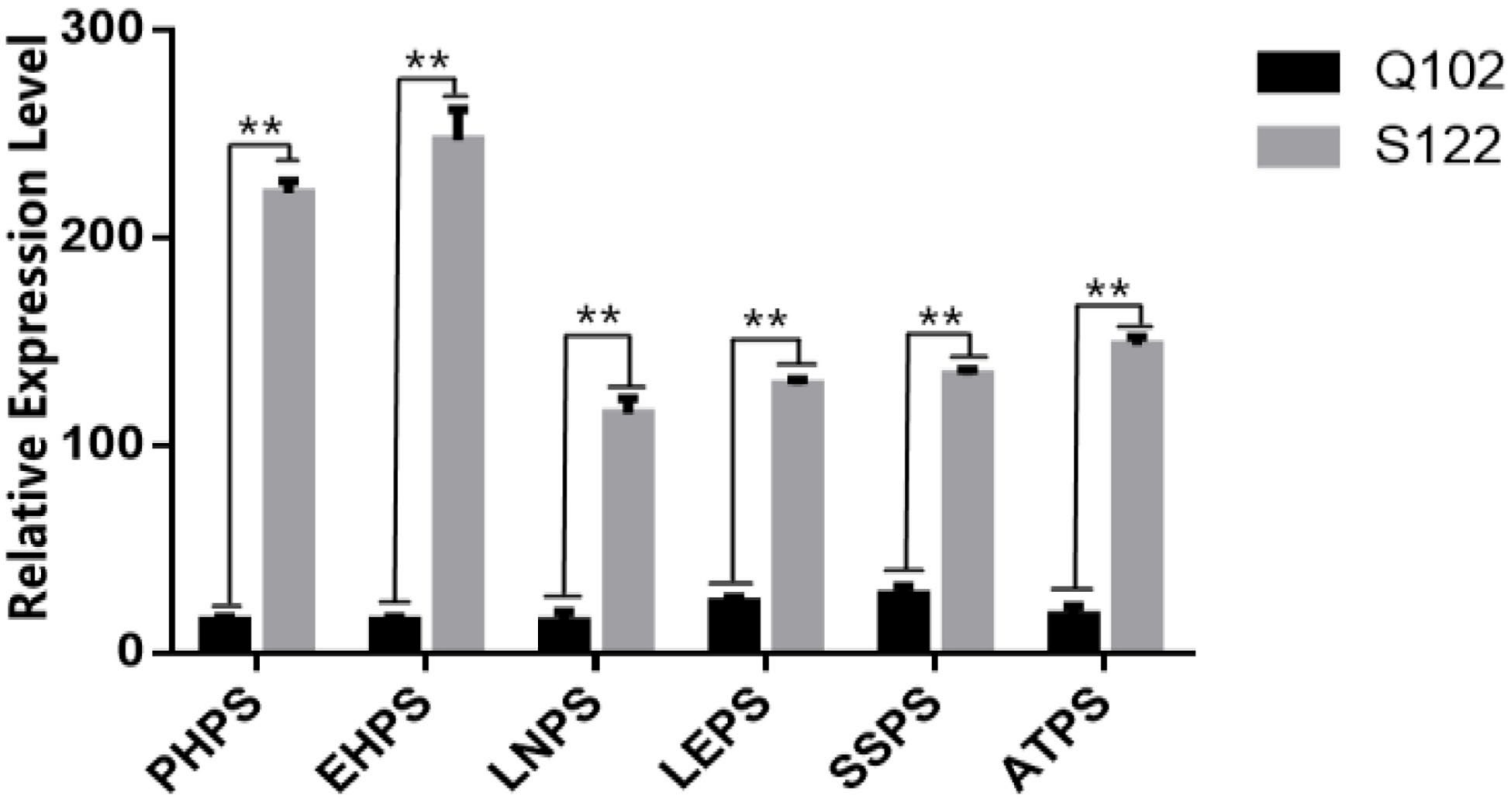
Difference analysis of photoperiod sensitivity between parents. The asterisks (^*^ or ^**^) represent the significant differences at *p* < 0.05 or *p* < 0.01, respectively.

The phenotypic data analysis for the parents showed that the phenotypes of S122 and Q102 were significantly different (Figure 2). The photoperiod sensitivity of S122 was higher than that of Q102 for six phenotypic traits (PHPS, EHPS, LNPS, LEPS, SSPS, and ATPS). Significant differences in expression between parents are more favorable for QTL mapping.

**Figure 3:**
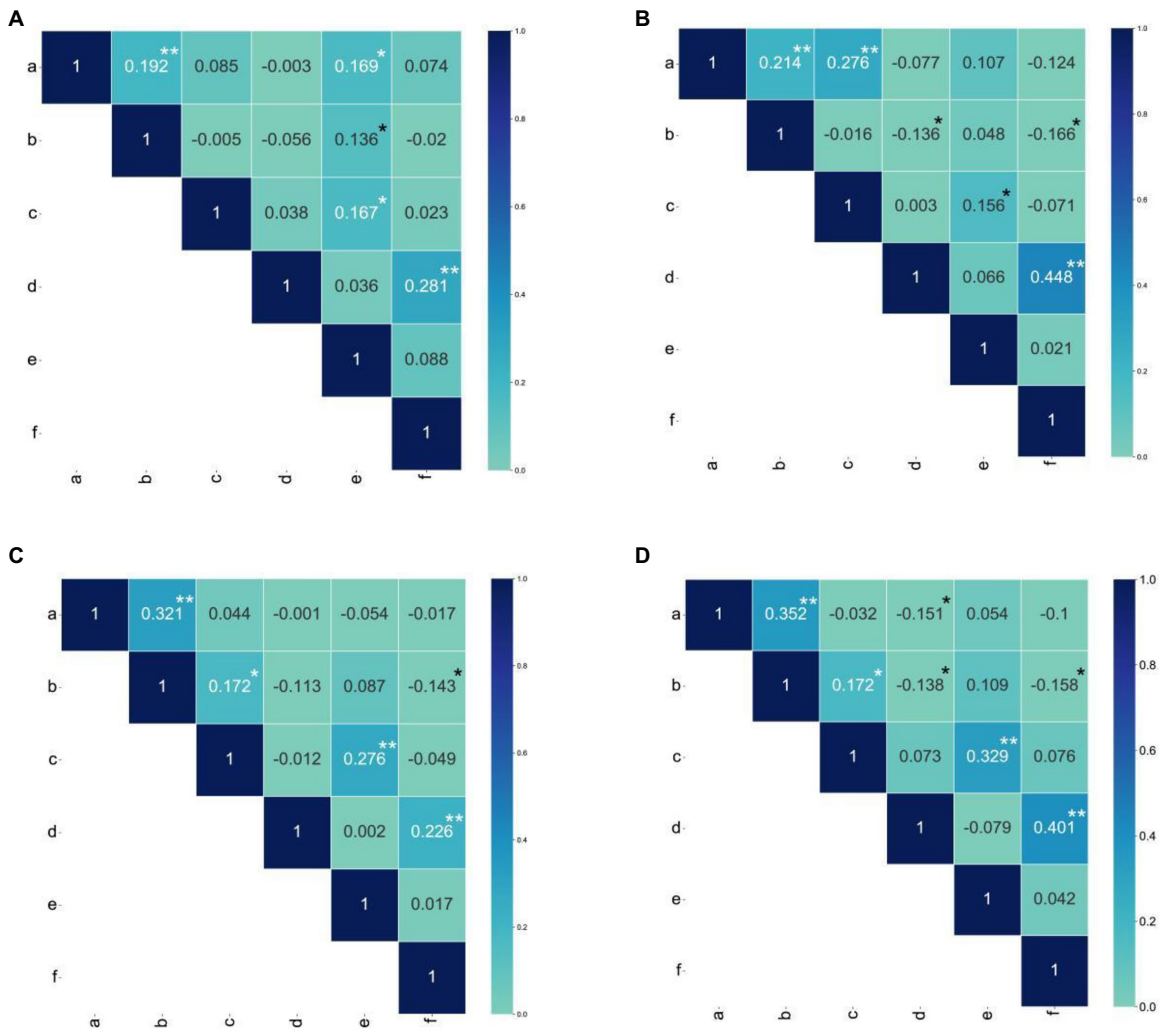
Pearson correlation coefficients among all photoperiod sensitivity traits. **(A)** Pearson correlation coefficients for photoperiod sensitivity traits between Hainan and Changchun in 2018. **(B)** Pearson correlation coefficients for photoperiod sensitivity traits between Hainan and Gongzhuling in 2018. **(C)** Pearson correlation coefficients for photoperiod sensitivity traits = between Hainan and Gongzhuling in 2019. **(D)** Pearson correlation coefficient for photoperiod sensitivity traits between Hainan and Changchunin, 2019. The a, b, c, d, e, and f in the figure represent PHPS, EHPS, LNPS, LEPS, SSPS, and ATPS, respectively. The asterisks (^*^ or ^**^) represent the significant differences at *p* < 0.05 or *p* < 0.01, respectively.

The skew and kurtosis of the PHPS, EHPS, LNPS, LEPS, SSPS, and ATPS phenotypic data were both less than 1 and were normally distributed (Figure 1; Supplementary Figure S1; Supplementary Tables S1, S2). The range of the photoperiod sensitivity data for the RIL populations was relatively large, and the coefficient of variation was small (Supplementary Tables S1). This result suggested that the phenotypic extreme values within the population were significantly different, and the median values were evenly distributed, which was in accordance with the expectations of the parental phenotypic analysis. Through phenotypic correlation analysis, there were significant correlations for PHPS and EHPS, LNPS and SSPS, and LEPS and ATPS in all four environments (Figure 3).

**Figure 4:**
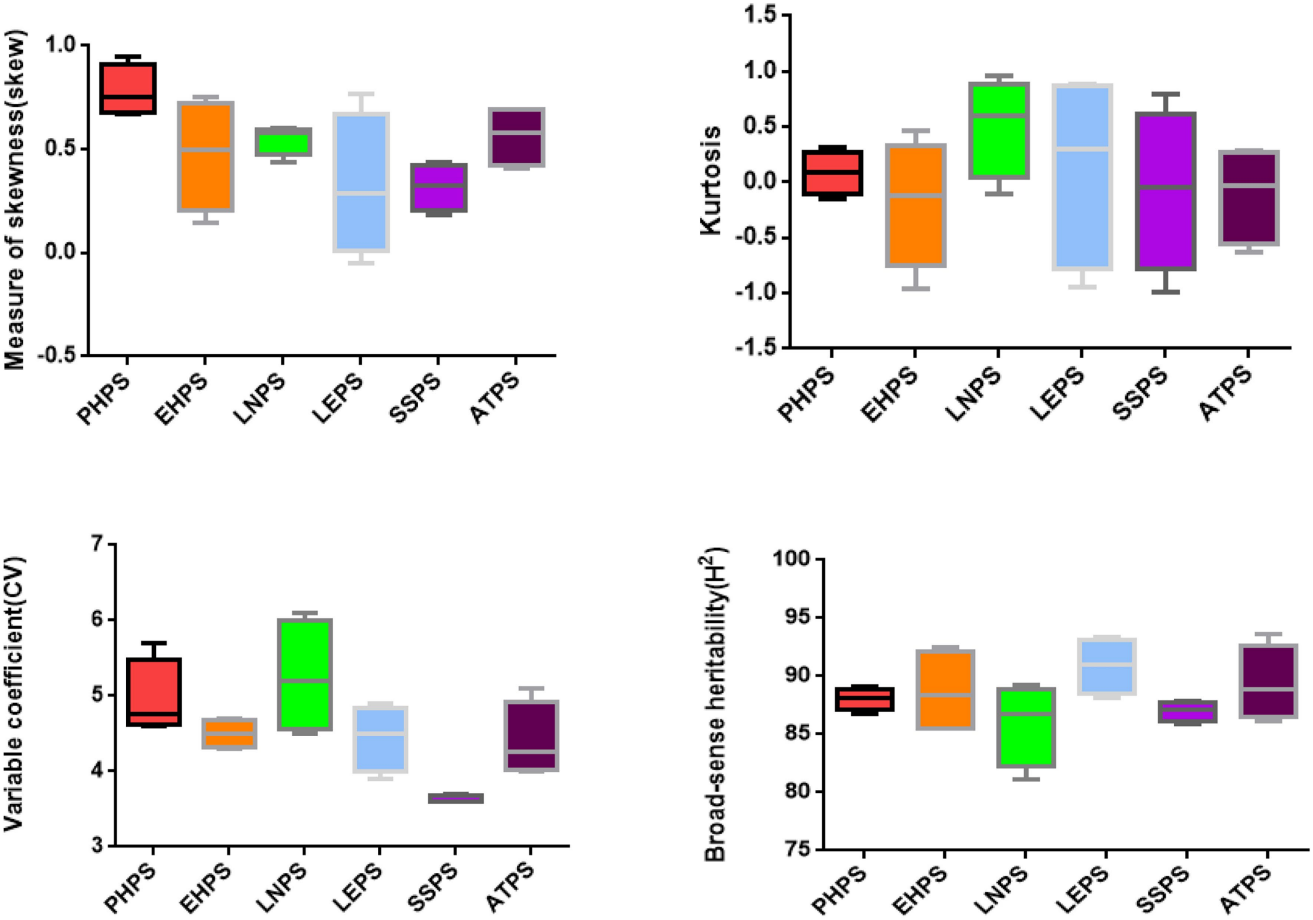
Descriptive statistics and broad-sense heritability for PHPS, EHPS, LNPS, LEPS, SSPS, and ATPS (boxplot).

These three groups of phenotypic correlations were less affected by the environment and were stable in different environments. However, the correlation of EHLS with ATLS, LNLS, and LELS phenotypes was influenced by the environment. The phenotypic correlation between EHLS and ATLS appeared in other environments except for 2020-HN-C (photoperiod sensitivity between Hainan and Changchun in 2020), while the phenotypic correlation between EHLS and LNLS and LELS appeared in only two environments (Figure 3). The heritability (H2) estimates in the individual environment ranged from 86.8 to 89.1% for PELS, from 85.5 to 92.5% for EHLS, from 81.1 to 89.2% for LNLS, from 89.8 to 93.4% for LELS, from 85.9 to 87.9% for SSLS, and from 86.1 to 93.6% for ATLS (Figure 4; Supplementary Table S1). Our data indicate that the photoperiod sensitivity of the RIL population exhibits significant natural variability and rich genetic diversity.

### Construction of a Genetic Linkage Map

A total of 199Gb raw data were obtained based on SLAF-seq, including 246,254 SLAF tags with a length of 414–464 bp. A total of 1,878,203,472 reads were obtained from parents and progeny, with an average Q30 of 95.39% and an average GC content of 45.98%, GC distribution was normal. The average sequencing depth of S122 and Q102 was 22-fold, and the average sequencing depth of the RIL population was 16.43-fold. A total of 19,624,498 single nucleotide polymorphism (SNP) markers was detected, of which 7,749,049 could be successfully typed, and there were 2,567,547 aa×bb types that could be used to construct genetic maps.

**Figure 5:**
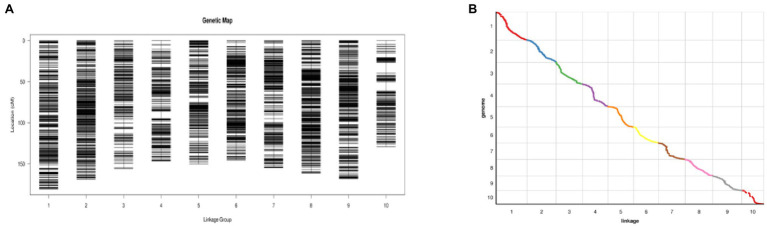
**(A)** High-density genetic map of the RIL population using SNP markers. The markers are indicated by black bars. The x-axis represents 10 linkage groups, and the y-axis represents genetic distance (Fei et al., 2022). **(B)** Genetic map and genome collinearity map. Both the x- and y-axes represent linkage groups (Fei et al., 2022).

Identifying 5,130 high-quality SNPs among 2,567,547 successfully typed SNPs was performed to construct a genetic map (Figure 5A). The total length of the genetic map was 1,560.80 cm, and the average distance between adjacent markers was 0.3 cm (Figure 5A). The greatest number of SNP markers was found on Chromosome 1, with 701 markers and a total length of 180.51 cm, and the average distance between markers was the shortest, 0.26 cm. Chromosome 10 was the shortest at 129.24 cm. The Spearman coefficient was used to measure the collinearity between the genetic map and the physical map. The closer the Spearman coefficient is to 1, the stronger the collinearity. The Spearman coefficients of 10 chromosomes were all higher than 0.99, with excellent collinearity (Figure 5B).

### QTL Mapping Using a High-Density Genetic Map

**Figure 6:**
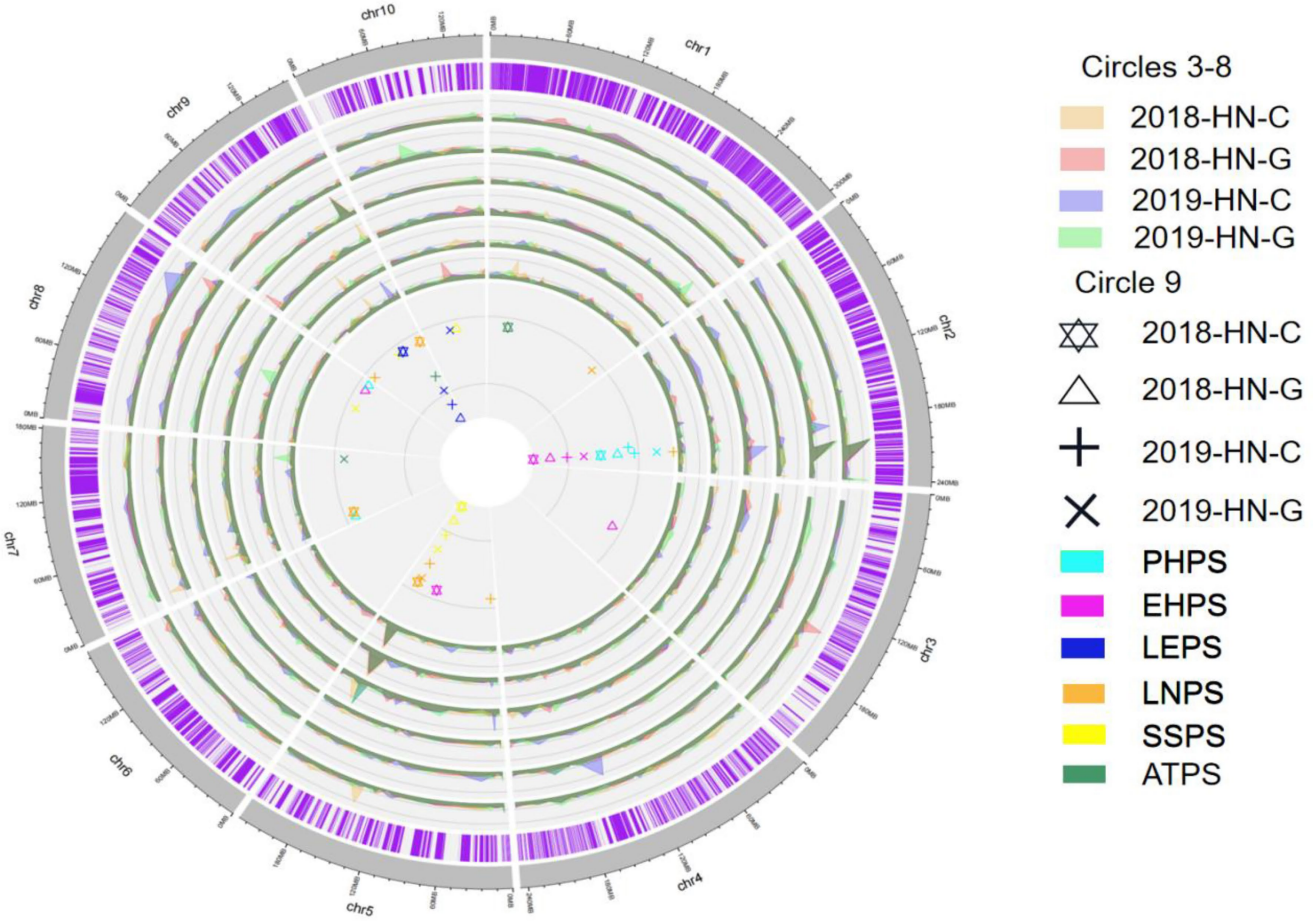
Photoperiod sensitivity-related QTL detected for the RIL population in the four environments. Circles 1–2 are chromosomes; Circles 3–8 are LOD values; Circle 9 indicates the QTL site.

Through QTL mapping, a total of 39 QTL related to six photoperiod sensitivity traits (PHPS, EHPS, LNPS, LEPS, SSPS, ATPS) in four environments were mapped for the RIL population (Supplementary Table S3; Figure 6). The LOD values ranged from 2.5233 to 21.707, and the phenotypic contribution rates ranged from 3.892 to 35.989% (Supplementary Figure S2).

For PHPS, a total of 7 QTL were identified on chromosome 2, 3, 5, and 8, and the phenotypic contribution rates ranged from 4.129 to 28.448% (Supplementary Table S3; Figure 6; Supplementary Figure S2). There is a consistent QTL region that was simultaneously located by four environments on chromosome 2, located in marker 19,204,093–1,914,755, with a phenotypic contribution rate of more than 20%, and its physical location is between 201,395,587 and 203,225,980 bp (Supplementary Table S3; Figure 6; Supplementary Figure S2). Among the eight QTL related to EHPS, there was also a consistent QTL on chromosome 2 at the same location as the consensus QTL for PHPS. In addition, there were two consistent QTL loci, located on chromosome 5 and chromosome 9, respectively. The consistent QTL loci on chromosome 9 were associated with LEPS and ATPS, and were co-localized by five QTL (qLEPS1-1, qLEPS2-1, qLEPS3-1, qLEPS4-1, qATPS3-1). The phenotypic contribution ranged from 22.327 to 35.239%, from marker 7,131,564 to marker 7,161,191, with a physical location at 132221790–141270207 bp (Supplementary Table S3; Figure 6; Supplementary Figure S2).

Additional consistent QTL were located on chromosome 5, which was simultaneously mapped by qLNPS3-3, qLNPS4-2, qSSPS1-1, qSSPS2-1, qSSPS3-1, and qSSPS4-2 (Supplementary Table S2; Figure 5; Supplementary Figure S2). The phenotypic contribution ranged from 16.5 to 33.5%, and the physical positions ranged from 188,722,771 bp to 190,587,817 bp. The additive effects of these three consistent QTL intervals were all negative, according to the B73 RefGen_v4 Gene model, with a total of 359 genes. The consistent QTL located on chromosomes 2 (201395587–203,225,980 bp), 5 (188722771–190,587,817 bp), and 9 (132221790–141,270,207 bp) contained 59, 41, and 259 genes, respectively (Supplementary Table S2; Figure 5; Supplementary Figure S2).

### Transcriptome Profiling

The change from the five-leaf stage to the six-leaf stage of maize denotes the change from vegetative growth to reproductive growth, which is very sensitive to photoperiod. To identify candidate genes for consistent QTL regions, we analyzed the transcriptome data for the leaves and SAMs of HZ4 and NIL H496 at the 5- and 6-leaf stages under LD light. At the 5-leaf stage, the newborn 5th leaf (L5) and SAM (S5) were governed by 3,562 and 5,646 differentially expressed genes, respectively (Supplementary Figure S5E). There were 1,477 upregulated genes and 2085 downregulated genes in leaves (Supplementary Figure S5A), and there were 2,768 upregulated expressed genes and 2,878 downregulated expressed genes in SAMs (Supplementary Figure S5C). The differentially expressed genes in leaves at the 6-leaf stage increased by 19% compared with the 5-leaf stage, and there were 4,422 genes in the newborn 6th leaf (L6), including 2,444 upregulated genes and 1978 downregulated genes (Supplementary Figures S5B,E). The differentially expressed genes in SAMs (S6) decreased by 48.3% compared with the 5-leaf stage, and there were only 2,914 genes, including 1,350 upregulated genes and 1,564 downregulated genes (Supplementary Figures S5C,E).

**Figure 7:**
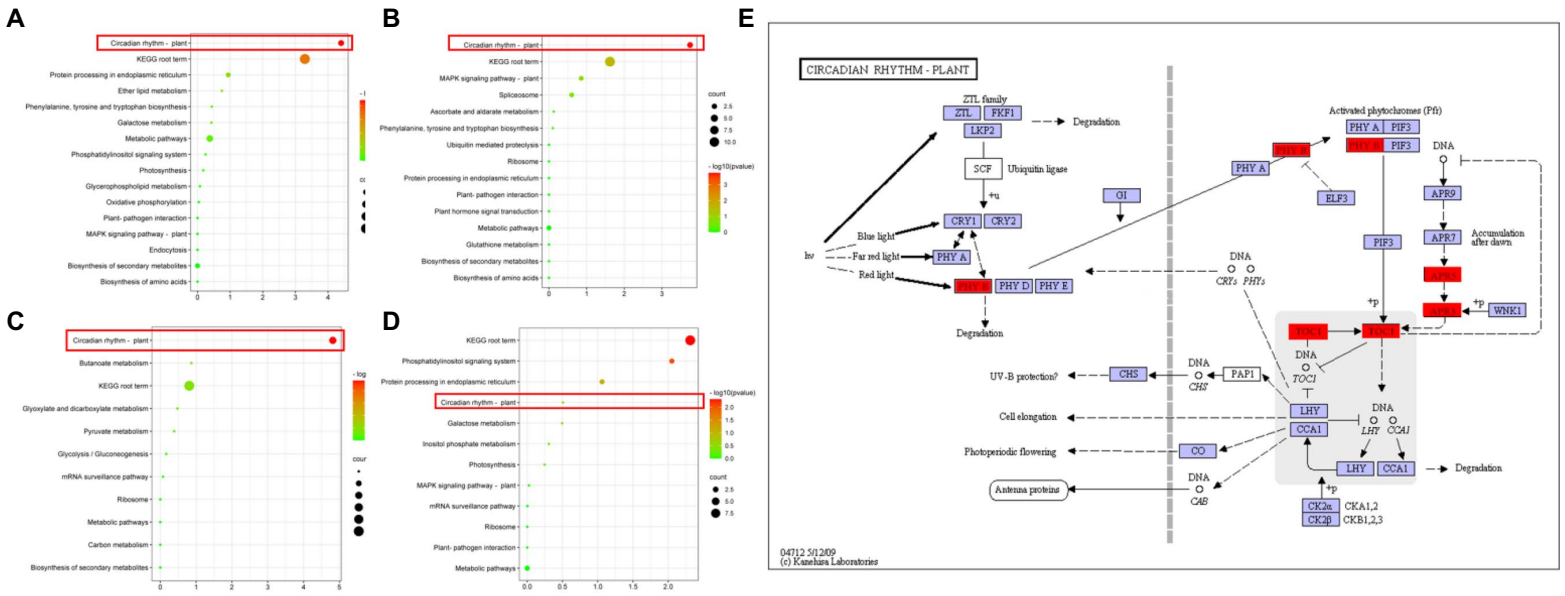
KEGG enrichment of combined consistent QTL and transcriptome analysis of intersecting genes. **(A)** KEGG enrichment of genes that intersect with the transcriptome of the consistent QTL region and the 5-leaf stage leaves. **(B)** KEGG enrichment that intersects with the transcriptome of the consistent QTL region and the 6-leaf stage leaves. **(C)** KEGG enrichment of genes that intersect with the transcriptome of the consistent QTL region and the 5-leaf stage SAMs. **(D)** KEGG enrichment of genes that intersect with the transcriptome of the consistent QTL region and the 6-leaf stage SAMs. **(E)** Plant circadian rhythm pathway. The positions marked in red are candidate genes (Kanehisa and Goto, 2000; Kanehisa, 2019; Kanehisa et al., 2021).

In the joint analysis of QTL mapping and transcriptome, as shown in the Venn diagram (Supplementary Figure S5E), there were 31, 35, 50, and 25 genes in the intersections between candidate genes of the consistent QTL region and differentially expressed genes of L5, L6, S5, and S6, respectively (Supplementary Figure S5E). The clustered heat map of the expression levels of these four groups of genes showed that the expression levels were significantly different between HZ4 and H496 (Supplementary Figure S3). These genes are candidate genes within the photoperiod-sensitivity-concordant QTL region and also differentially expressed genes in response to photoperiod changes. Although no significant photoperiod-related GO functional annotations were found among these genes (Supplementary Figure S4), four genes (Zm00001d006212, Zm00001d017241, Zm00001d047761, and Zm00001d047632) were annotated to the KEGG (Figure 7) plant circadian rhythm pathway (ko04712).

The plant circadian pathway is an endogenous regulatory pathway used by plants to respond to changes in the environmental photoperiod, and is also known as the photoperiod regulation pathway. Therefore, we hypothesized that four genes (Zm00001d006212, Zm00001d017241, Zm00001d 047761, and Zm00001d047632) are the key genes control the photoperiod sensitivity of maize, and subsequently analyzed their expression patterns.

### Candidate Gene Expression Pattern Analysis

The expression pattern of a gene can reflect the degree of association of a gene with a phenotype under a specific stress. The highest expression in leaves was that of Zm00001d047761, Zm00001d006212, and Zm00001d017241, and the highest expression in embryos under LD light was that of Zm00001d047632 (Supplementary Figure S7A). Therefore, we selected the leaves of Q102 and S122, and analyzed the expression patterns of Zm00001d006212, Zm00001d017241, Zm00001d047761, and Zm00001d047632 under LD and SD conditions. The qPCR results showed that Zm00001d006212, Zm00001d017241, Zm00001d047761, and Zm00001d047632 exhibited little difference in the expression peaks between S122 and Q102 under SD conditions, but the expression peaks appeared at different times of the day (Supplementary Figure S7C).

The peak expression levels of Zm00001d047761, Zm00001d006212, and Zm00001d017241 appeared at 10 o’clock, 8 o’clock, and 0 o’clock, respectively. Under LD light, except for Zm00001d047632, the expression differences of other genes between S122 and Q102 were significantly greater at the peak than those under SD light (Supplementary Figure S7B). This result suggested that Zm00001d047761, Zm00001d006212, and Zm00001d017241 are likely to be the key genes affecting the photoperiod sensitivity of maize under different light conditions. Therefore, we chose to verify the functionality of Zm00001d047761, Zm00001d006212, and Zm00001d017241.

### Phylogenetic and Conserved Domain Analysis

As a subfamily of the CCT family, the PRR family contains CCT conserved domains in all genes. In addition, all genes contain REC protein receptors, which may be their specific domains that distinguish them from other CCT family genes (Supplementary Figure S6). Zm00001d047761 (*ZmPRR73*) is highly homologous to *OsAPRR3* (XP006649884.1) in rice and *AtPRR7* (AT5G02810) in *Arabidopsis thaliana*, with homology rates of 80 and 74%, respectively. The homology rate of Zm00001d006212 (*ZmPRR95*) is 58% with Arabidopsis PRR5 (*AT5G24470*), and the homology rate with rice *APRR9* (XP01511684.1) is 66%. There is also satisfactory homology between Zm00001d017241 (*ZmTOC1b*) and *TOC1* (AT5G61380) of Arabidopsis. The more closely related the species, the more conserved the homologous genes. The conservation of homologous genes among species determines the functional similarity. These results can provide important guidance and basis for the functional verification of Zm00001d047761, Zm00001d006212, and Zm00001d017241.

### Sequence Comparison Analysis of Parental Target Gene and Promoter Region

Through comparing the sequences encoded by the target genes between the parents, we found that the sequence homology rate of the object genes (*ZmTOC1b, ZmPRR73, and ZmPRR95*) between the parents was 100% (Supplementary Figure S13). We selected the 2000 bp sequence before the coding region of the object gene as the promoter region of the object gene for comparison and analysis. The comparison results showed that there were difference in the promoter region of the object gene between the parents (Supplementary Figure S14). We also analyzed cis-acting elements in the promoter region of the object gene. *ZmPRR95* promoter region of between the parents difference are located on the cis-acting element circadian (cis-acting regulatory element involved in circadian control) and CAAT-box (common cis-acting element in promoter and enhancer regions), respectively (Supplementary Figure S14A; Supplementary Table S9). *ZmPRR73* also has sequence differences in the same two cis-elements (Supplementary Figure S14B; Supplementary Table S10). There are two sequence differences in the promoter region of *ZmTOC1b* between the parents, which are located on the two cis-acting elements of CAAAT-box and GATA-motif, respectively (Supplementary Figure S14C; Supplementary Table S10).

### Expression Vector Validation and Molecular Detection of Transgenic Progeny

Plant expression vectors (pCambia3301-ZmTOC1b-3His, pCambia3301-ZmPRR73-3His, and pCambia3301-ZmPRR95-3His) and prokaryotic expression vectors (PET-22b-ZmTOC1b-6His, PET-22b-ZmPRR73-6His, and PET-22b-ZmPRR95-6His) were verified by enzymatic digestion. The CRISPR/Cas9 knockout vectors (pCRISPR/Cas9-g^ZmTOC1b-1^-g^ZmTOC1b-2^, pCRISPR/Cas9-g^ZmPRR73-1^ -g^ZmPRR73-2^, and pCRISPR/Cas9-g^ZmPRR95-1^-g^ZmPRR95-2^) were PCR-tested with universal primers and detected by Sanger sequencing (Supplementary Figures S11A,C,E,G,I,K). All CRISPR/Cas9 editing vectors are single-gene dual-target vectors.

The plant expression vectors and the CRISPR/Cas9 knockout vectors were transferred into Dan 988 s transgenic recipient material by genetic transformation (Supplementary Figure S9), and the T0 generation was obtained and analyzed by PCR (Supplementary Figure S8). The following plants were obtained: 5 positive plants of ZmTOC1b-OE were detected by PCR; 2 positive plants of ZmPRR73-OE were detected by PCR; 3 positive plants of ZmPRR95-OE were detected by PCR; 2 positive plants of ZmPRR95-KO were detected by PCR; 1 positive plant of ZmPRR73-KO was detected by PCR; and 1 positive plants of ZmTOC1b-KO were detected by PCR (Supplementary Figures S8B,C).

The results of His-tag western hybridization of the T2 generation of overexpression-positive plants showed that the target gene had been integrated into the genome of the recipient material and could be normally expressed (Supplementary Figure S10). Further analysis of the T2 generation mutant plants showed that g^ZmTOC1b-1^ had 1 bp replaced and 1 bp deleted. The g^ZmTOC1b-2^ target had 1 bp replaced. Both targets of g^ZmPRR73-1^ and g^ZmPRR73-2^ had 1-bp deletions. The g^ZmPRR95-1^ target had 1 bp replaced, and the g^ZmPRR95-2^ target had two bases deleted (Supplementary Figure S11).

### Modification of Vegetative Traits in T2 Maize Transgenic Plants

**Figure 8:**
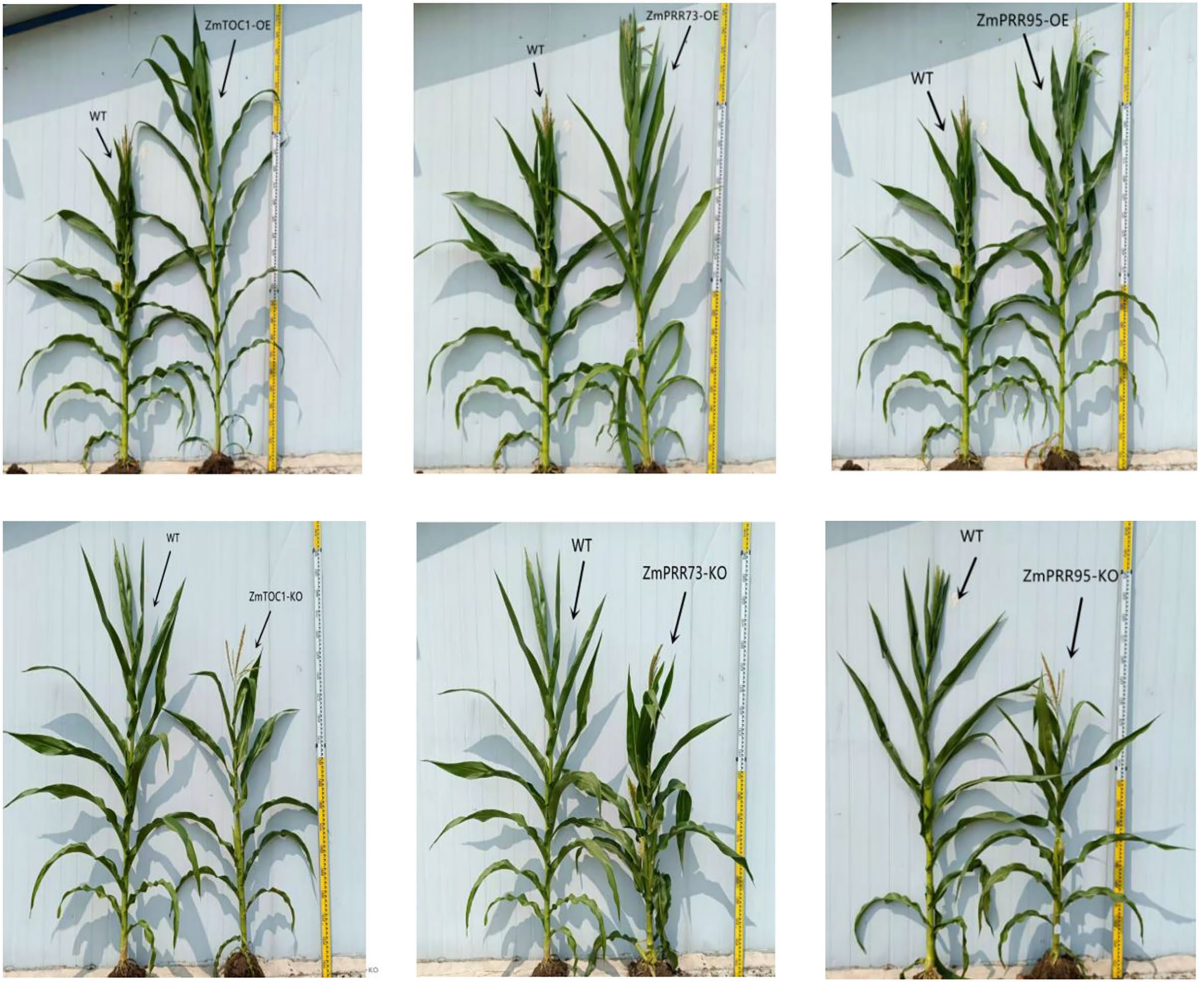
Phenotypic differences between positive plants and WT.

The phenotype of transgenic plants was significantly different from that of WT. The mean plant heights of ZmTOC1b-OE, ZmPRR73-OE, and ZmPRR95-OE were 267 cm, 258 cm, and 272 cm, respectively, which were 22.7, 18.6, and 21.3% higher than WT, respectively. In contrast, the mean plant height of ZmTOC1b-KO, ZmPRR73-KO, and ZmPRR95-KO was 20, 22.8, and 25.7% shorter than that of WT, respectively. The mean ear height of WT was 82 cm, while the mean ear heights of ZmTOC1b-OE, ZmPRR73-OE, and ZmPRR95-OE were 127, 119, and 124, respectively, which were significantly higher than those of the control group. The mean ear height of plants with knockouts of the target gene was significantly lower than that of WT (Figure 8).

The total number of leaves of the overexpression plants was 3–6 more than that of the WT, and 3–4 less than that of the WT for plants with knockouts of the target gene. For the number of leaves under the panicle, there were 3–5 more than WT for the overexpression plants, and 1–2 less than WT for the knockout plants. The mean silking and anthesis stages for ZmTOC1b-OE, ZmPRR73-OE, and ZmPRR95-OE were 5–7 days and 5–8 days later than WT, respectively, while the ASI was not much different from WT, and remained within 3 days. Infertility was caused by incompatibility between males and females. The plants with knockouts of the target gene were 9–14 days and 4–6 days earlier than WT, respectively. The ASI (anthesis stage and silking stage intervalof ZmPRR73-KO was 5 days, and some individuals appeared sterile; Supplementary Figures S11, S12).

**Figure 9:**
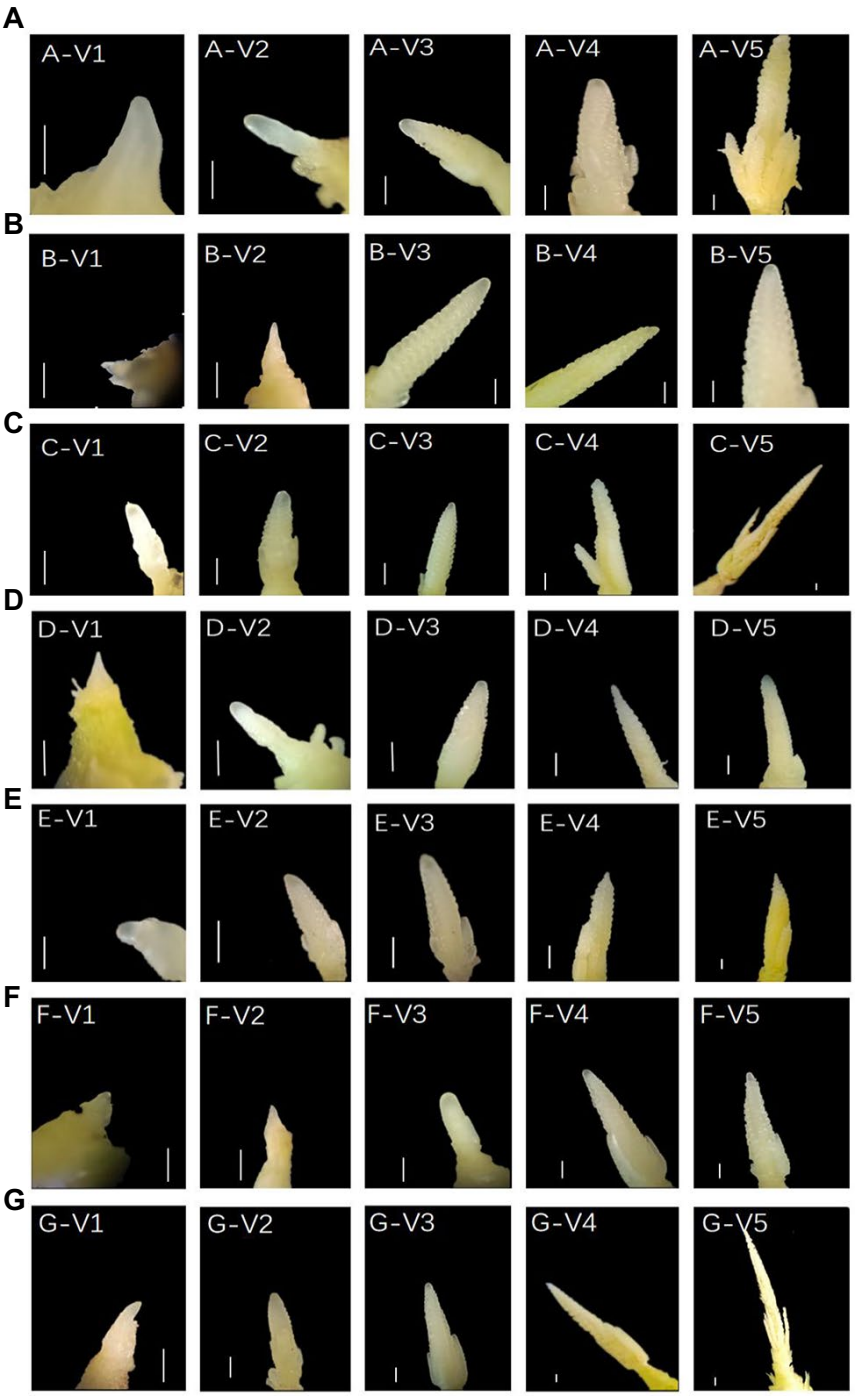
Anatomical diagram of transgenic-positive plants and WT shoot apical meristems (SAMs). **(A–G)** represent WT, ZmTOC1-OE, ZmTOC1-KO, ZmPRR37-OE, ZmPRR37-KO, ZmPRR95-OE, and ZmPRR95-KO, respectively. V1, V2, V3, V4, and V5 represent days 23, 27, 30, 33, and 36 post-emergence, respectively. Scale bars (V1,V2,V3, V4,V5) = 500 μm.

To capture the changes in meristems during and after floral transition, we dissected the SAMs of T2 generation positive and control plants (Figure 9). WT plants were from the Dan988a line, with SAMs transitioning from vegetative growth to reproductive growth between the V2 and V3 stage, followed by branch meristem initiation at V3-V4. By the V4-V5 stage, the meristem developed and formed to tassel. Plants of ZmTOC1b-KO, ZmPRR73-KO, and ZmPRR95-KO developed very rapidly. Although we separately knocked out three different genes, their SAM developmental processes were strikingly similar. The floral transition occurred at the V1-V2 stages, one stage faster than that of the WT plants. By the V4-V5 stages, the immature tassel was fully formed and committed to maturation. Despite their earlier development, the tassels did not exhibit many branches. Compared with WT, the immature tassels of ZmTOC1b-KO, ZmPRR73-KO, and ZmPRR95-KO were longer and slender.

The growth rate of overexpression plants was significantly slower than that of WT, and the development rates of positive plants with different genes were slightly different. The slowest development was observed for plants of ZmTOC1b-OE, and no obvious differentiation was found in the V5 meristem. The growth rate of ZmPRR73-OE plants was three stages later than that of WT. At the V4-V5 stage, the SAMs changed from engaging in vegetative growth to engaging in reproductive growth, and the meristem began to differentiate into collaterals, and gradually formed immature tassels at the V5 stage. The developmental speed of ZmPRR95-OE plants was two stages later than that of WT, and the floral transition occurred between the V3 and V4 stage. Development retardation during the V4-V5 stages occurred, and the immature tassel did not continue to differentiate and develop. Overall, ZmTOC1-OE, ZmPRR73-OE, and ZmPRR95-OE delayed SAM development and differentiation into tassels, whereas knocking them out accelerated tassel differentiation and maturation.

**Figure 10:**
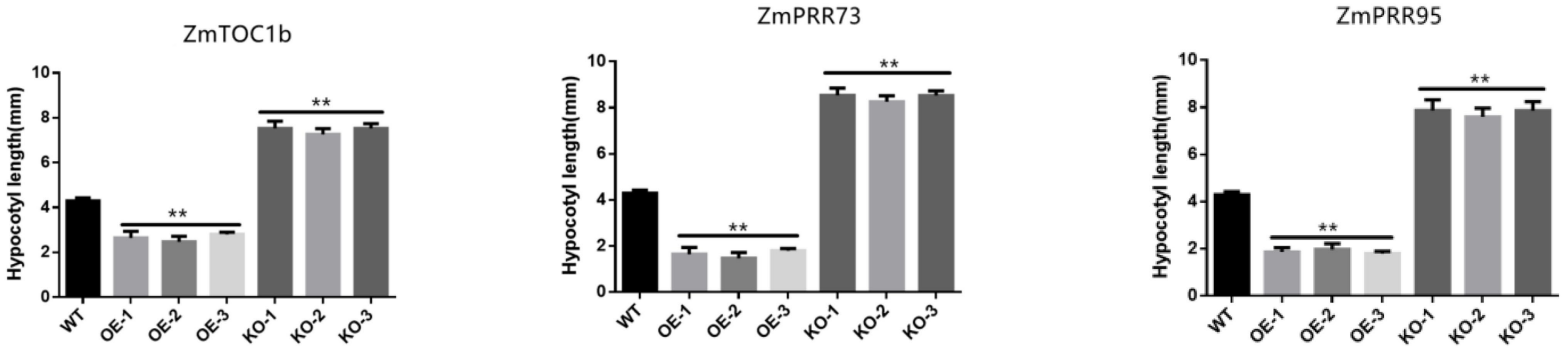
Comparison of maize hypocotyl lengths of transgenic-positive plants. The asterisks (^*^ or ^**^) represent the significant differences at *p* < 0.05 or *p* < 0.01, respectively.

**Figure 11:**
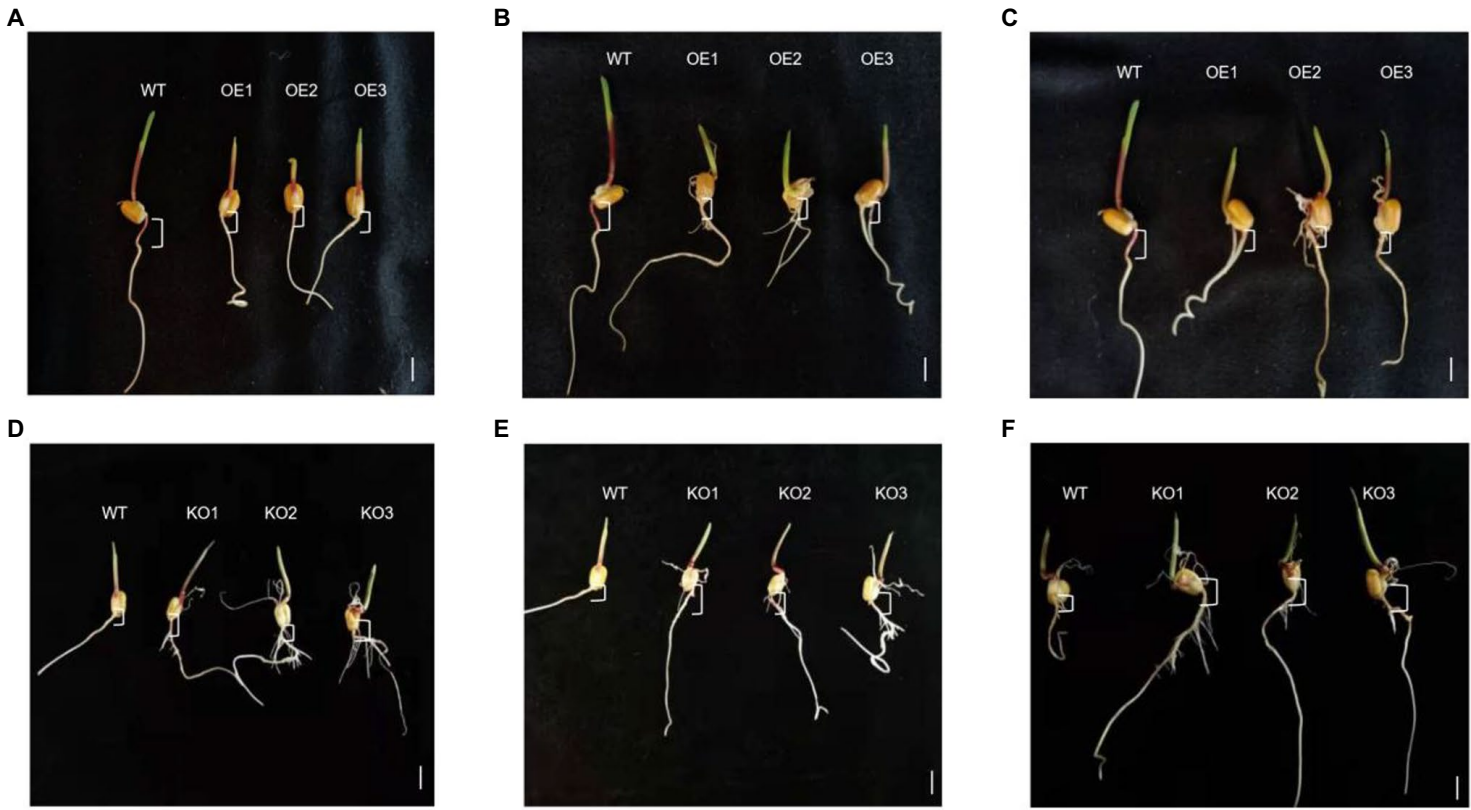
Hypocotyls of transgenic positive plants and WT under LD. **(A)** represents the comparison of ZmTOC1b overexpressing positive shoots with WT hypocotyls. **(B)** represents the comparison of ZmPRR73 overexpressing positive shoots with WT hypocotyls. **(C)** represents the comparison of ZmPRR95 overexpressing positive shoots with WT hypocotyls. The positive seedlings knocked out of ZmTOC1b in **(D)** generation were compared with WT hypocotyls. The positive seedlings knocked out of ZmPRR73 in **(E)** generation were compared with WT hypocotyls. The positive seedlings knocked out of ZmPEE95 in **(F)** generation were compared with WT hypocotyls. Scale bars **(A–C)** = 5 mm, **(D–F)** = 1 cm.

Hypocotyl elongation is regulated by photoperiod, and maize hypocotyls show sensitivity to photoperiod under LD light. Compared with the WT, the hypocotyl lengths of ZmTOC1b-OE, ZmPRR73-OE, and ZmPRR95-OE were 52.3, 65.9, and 38.5% shorter, respectively, under LD light. The hypocotyl lengths of ZmTOC1b-KO, ZmPRR73-KO, and ZmPRR95-KO were 48.8, 50.6, and 38.9% longer than WT, respectively (Figures 10, 11).

### Expression Pattern Analysis of Objective Genes

**Figure 12:**
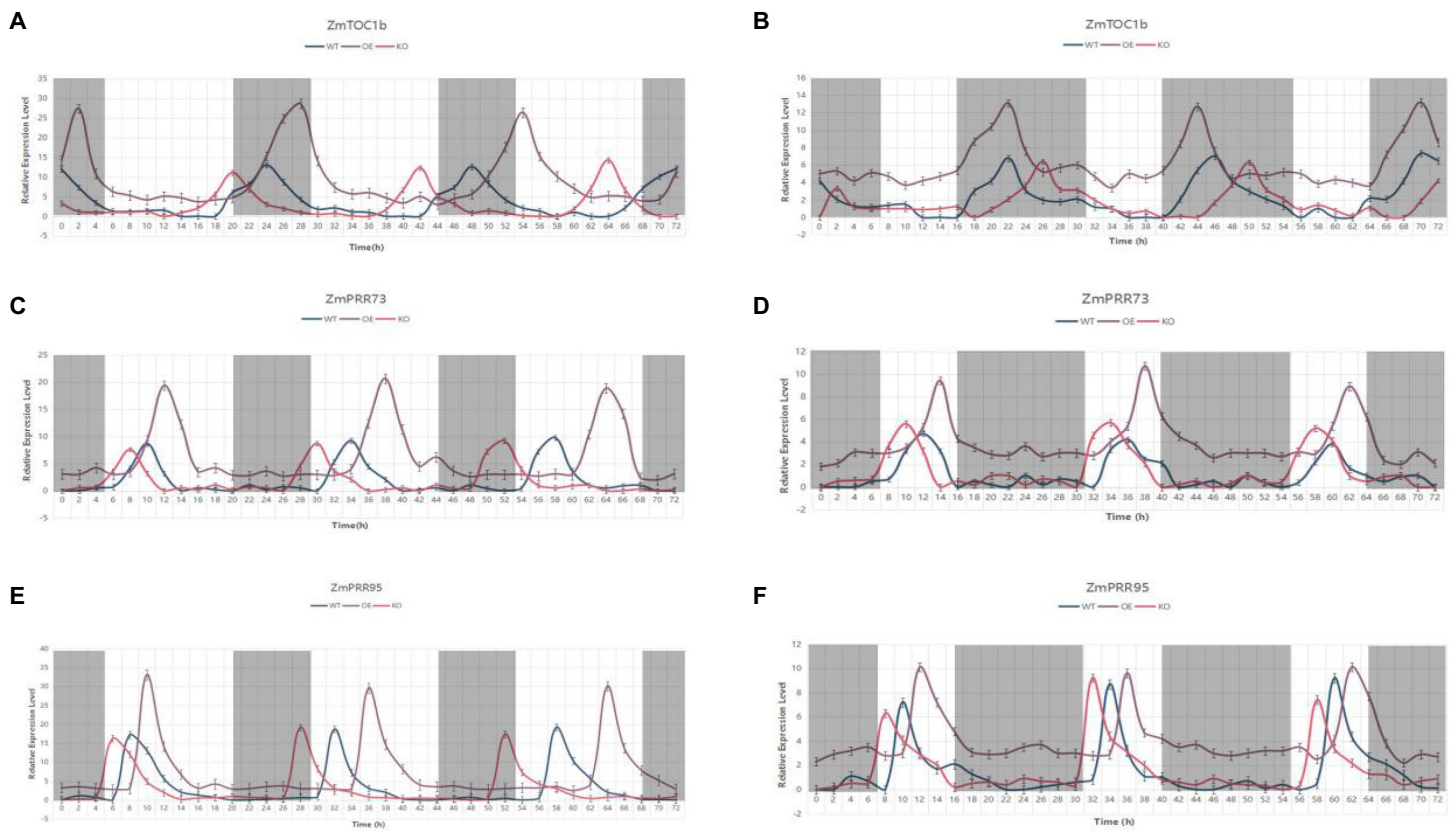
The expression pattern analysis of the objective gene in transgenic positive plants and the WT under long-day and short-day light for 72 h. **(A)** Expression pattern analysis of ZmTOC1-positive plants and the WT under LD light. **(B)** Expression pattern analysis of ZmTOC1-positive plants and the WT under SD light. **(C)** Expression pattern analysis of ZmPRR73-positive plants and the WT under LD light. **(D)** Expression pattern analysis of ZmPRR73-positive plants and the WT under SD light. **(E)** Expression pattern analysis of ZmPRR95-positive plants and the WT under LD light. **(F)** Expression pattern analysis of ZmPRR95-positive plants and the WT under SD light. WTs are plotted in blue, objective gene overexpression-positive plants are plotted in violet, and objective gene knockout-positive plants are plotted in red.

The KEGG annotation results showed that *ZmTOC1b*, *ZmPRR73*, and *ZmPRR95* are all on the plant circadian rhythm pathway. To verify whether they have circadian rhythm oscillations, they were subjected to expression pattern analysis. We used WT, overexpression plants, and target gene knockout plants as materials under LD and SD conditions, sampling every 2 h for 72 h. *ZmTOC1b*, *ZmPRR73*, and *ZmPRR95* exhibited stable rhythmic expression under LD and SD, but there were differences in period, phase position, and amplitude (Figure 12).

The peak expression of *ZmTOC1* under SD light in the WT was at 10 p.m., and the relative expression amplitude was 3.52. Under LD light, the relative expression amplitude was 6.43. In addition, under LD light, the expression level of ZmTOC1b-OE was significantly higher than that of WT, and the period increased by 2 h compared with WT, and therefore, the phase also changed. On the contrary, the expression period of ZmTOC1-KO was decreased by 2 h compared with the WT. Under SD light, the expression level of ZmTOC1b-OE was still significantly higher than that of WT, but with a similar amplitude. Although the expression period was the same as that of WT and ZmTOC1b-KO, the phase was still different. Unlike ZmTOC1b, ZmPRR73 is expressed during the day, and its peak expression generally appears at approximately 10 am under LD light. Under SD light, the expression phase of ZmPRR73 was delayed by 2 h. Under LD light, the expression period of ZmPRR73-OE was prolonged by 2 h, and the phase also irregularly changed.

The expression amplitude of ZmPRR73-OE was 47.1% higher than that of WT and ZmPRR73-KO. The expression amplitude of ZmPRR73-KO was basically the same as that of WT, but the period was 2 h shorter than that of WT, and the phase was also shifted. However, the peak expression of ZmPRR73-OE was still higher than that of WT and ZmPRR73-KO under SD light, and the amplitude and period were basically consistent. The expression level of ZmPRR95 generally peaked 4 h after sunrise. The expression period of ZmPRR95-OE was 26 h under LD conditions, which delayed the phase, and expression amplitude was significantly higher than that of WT and ZmPRR95-KO. The expression pattern under SD light was similar to that of ZmPRR73 except for the phase difference.

### Analysis of Expression Patterns of Genes Related to Photoperiod Regulation in Transgenic Plants

**Figure 13:**
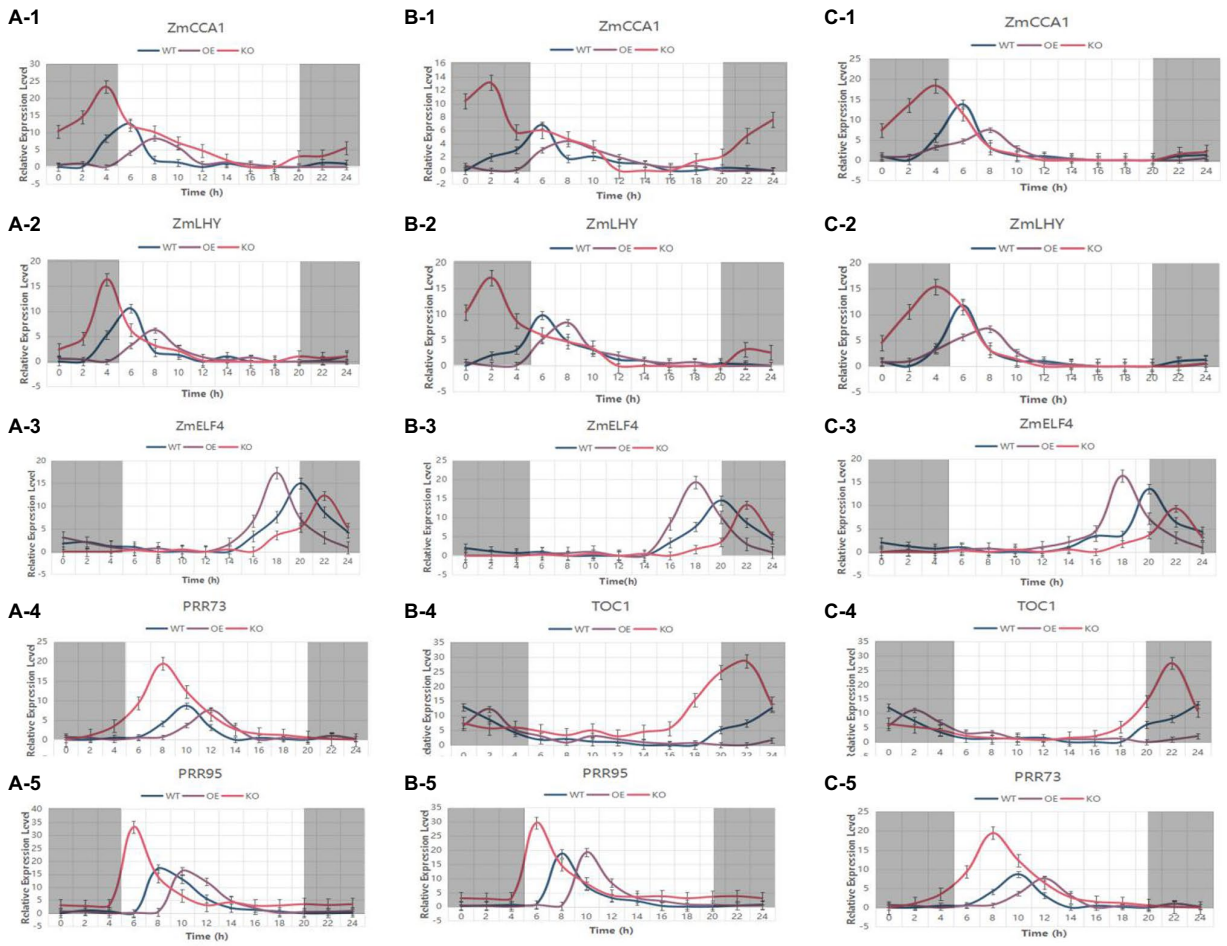
The expression patterns of circadian rhythm genes in transgenic-positive plants and the WT were analyzed under LD light for 24 h. **(A–C)** represent ZmTOC1, ZmPRR73, and ZmPRR95 transgenic-positive plants, respectively. WTs are plotted in blue, objective gene overexpression-positive plants are plotted in violet, and objective gene knockout-positive plants are plotted in red.

In order to deeply study how the target gene is regulated in the circadian rhythm pathway, genes with known or predicted functions were selected in the circadian rhythm pathway. Under LD light, the leaves of the transgenic T2-generation-positive plants and the WT at the 5-leaf stage were used as materials, and samples were obtained every 2 h for 24 h (Figure 13). The expression patterns of *ZmCCA1* and *ZmLHY* were similar, with peak expression appearing at 4 p.m. in the WT. The peak expression was delayed by 2 h in ZmTOC1-OE, ZmPRR73-OE, and ZmPRR95-OE plants, and the peak expression was decreased as compared with the WT. In ZmTOC1-KO, ZmPRR73-KO, and ZmPRR95-KO plants, the expression levels of *ZmCCA1* and *ZmLHY* were significantly higher than those in WT, and the phases were brought forward, which was most prominent in ZmPRR73-KO. *ZmELF4* was expressed at night. In the WT, the peak expression appeared at 8 p.m. In ZmTOC1-OE, ZmPRR73-OE, and ZmPRR95-OE plants, the expression phase was 2 h earlier than that in WT, and the expression was significantly increased. In contrast, in ZmTOC1-KO, ZmPRR73-KO, and ZmPRR95-KO plants, the expression phase was delayed, and the expression level was significantly lower than that in WT.

The expression pattern of *ZmTOC1* in ZmPRR73-OE and ZmPRR95-OE plants was also significantly affected. The expression level was significantly increased compared with that in WT, and the expression phase was delayed by 2 h and 4 h, respectively. In ZmPRR73-KO and ZmPRR95-KO plants, *ZmTOC1* exhibited a decreased expression peak and phase brought forward compared with WT plants. The expression patterns of *ZmPRR73* and *ZmPRR95* in transgenic plants were similar to that in *ZmTOC1*.

We also found that the expression of these genes appeared to be continuous throughout the day. In the morning, *ZmPRR95* first reached peak expression at 8:00 am, *ZmPRR73* reached its peak at 10:00 am, *CCA1* and *LHY* simultaneously peaked at 2:00 p.m., and these four genes were expressed during the day. After sunset, *ZmELF4* expression peaked at 8:00 p.m., followed by *ZmTOC1* expression at 12:00 am, and they were all expressed at night. Overall, the expression patterns of these genes were correlated with each other, and in knockout or overexpression plants, the expression underwent a chain reaction, with the peak expression levels and phases regularly changing.

## Discussion

Maize is an important food and industrial raw material, and the demands for greater maize yields continue to increase year by year (Upadyayula et al., 2006). The influence of heterosis on increasing yield is gradually weakening, and narrow germplasm resources limit the utilization of heterosis. The hybridization of germplasm resources between temperate and tropical environments is an important method to improve heterosis. Therefore, many studies have been carried out, but they are limited to the flowering period of maize (Jung and Muller, 2009; Higgins et al., 2010; Zhang et al., 2011). However, photoperiod sensitivity is the main factor that determines the adaptation of tropical varieties in temperate zones. Passivating the photoperiod sensitivity of tropical germplasm resources is the key to solving this problem.

Photoperiod sensitivity is an abstract concept that is difficult to measure using a single phenotype. Therefore, we introduced a photoperiod sensitivity calculation formula for the first time (RD (%) = [(L-S)/S] × 100), and using phenotypes of different latitudes to comprehensively measure the photoperiod sensitivity of the population, this phenotypic data can be used for QTL mapping. A total of 39 QTL were mapped (Supplementary Table S2). Among the QTL related to PHPS and EHPS, we found consistent QTL (cQTL1) located on chromosome 2, from 201,395,587 bp to 203,325,980 bp. Mu et al. (2010) constructed a F2:3 family population using Zheng 58 and Ya 8,701 as parents, identified a consistent QTL related to photoperiod sensitivity in both environments, and also noted that the physical position coincided with cQTL1.

In the analysis of phenotypic correlation, there was a stable and significant correlation of PHPS and EHPS in the four environments, and the generalized heritability was higher than 85%. This indicates that the genetic loci controlling the two traits were less affected by the environment, and not only exert pleiotropic effects, but also may be linked to each other (Figure 3; Supplementary Table S1). A consistent QTL (cQTL3) related to LEPS and ATPS was identified that stably exists in multiple environments in the Bin9.06 region of chromosome 9. Bin9.06 is a hot spot region for QTL, and there are 12 QTL- and 2 meta-QTL-related photoperiod-sensitive genes in this region (Xu et al., 2012). Wang et al. (2010) used simple sequence repeat (SSR) molecular markers to identify consistent QTL (umc1495-umc1732) in multiple environments, and the physical position overlapped with cQTL3. We also identified new consistent QTL (cQTL2) related to SSPS and LNPS, located in the region 188,722,771–190,587,817 of chromosome 5. There are no indel differences in the alleles found among the 358 genes in the cQTL1, cQTL2, and cQTL3 regions, and three objective genes that positively responded to photoperiod changes were identified through combined transcriptome analysis and expression pattern analysis. In the sequence comparison of the target gene and the promoter region between the parents, we found that there was no difference in the target gene sequence between the parents, but the promoter region showed obvious differences and were located in important cis-acting elements. Thus, we concluded that the objective gene affects the photoperiod sensitivity of maize through expression patterns, rather than structural differences between alleles (Supplementary Figure S5).

Numerous studies have shown that circadian rhythms play an important role in regulating the photoperiod sensitivity of crops (Ellis et al., 1992a). Circadian rhythm is an endogenous regulatory system in plants that assists them in adapting to the environment by sensing changes in the external photoperiod so that they can adequately respond to seasonal changes (Greenham and McClung, 2015). In the KEGG annotation, we found that all objective genes were annotated in the circadian rhythm regulatory pathway, and all were in the feedback inhibition loop of the core oscillator (Figure 7). In order to further study the effect of the objective gene on the photoperiod sensitivity of maize, we overexpressed and knocked out *ZmTOC1b*, *ZmPRR73*, and *ZmPRR95*, and observed the effect on the plant receptors. ZmTOC1b-OE, ZmPRR73-OE, and ZmPRR95-OE all showed sensitivity to photoperiod under LD conditions, and overexpressed plants became taller, delayed flowering, and increased their leaf number compared to WT, while the opposite was observed for ZmTOC1b-KO, ZmPRR 73-KO, and ZmPRR95-KO (Supplementary Figure S10).

An expression pattern analysis of the transgenic-positive plants for 72 h showed that the expression patterns of the three objective genes were similar and rhythmically expressed, but the expression times were different. The expression peaks of ZmPRR73 and ZmPRR95 were at 8 am and 10 am, respectively, and the expression of ZmTOC1b was inhibited (Figure 12). The expression of ZmPRR73 and ZmPRR95 was also inhibited. The overexpression and knockout of the objective gene affected the phase, amplitude, and period of rhythmic expression (Figure 12). Under LD light, the expression level of the objective gene in the overexpressed plants increased and continued to be expressed, the period was prolonged by 2 h, and the phase was also delayed. In plants with knockout of the objective gene, the expression level of the target gene was similar to that of the WT, but the expression period was shortened and the phase was earlier. This difference was attenuated under SD conditions (Figure 12). Therefore, we concluded that the objective gene plays a negative regulatory role in photoperiod sensitivity, and overexpression of the objective gene will result in increased sensitivity to photoperiod in the recipient plants.

Interestingly, the overexpression and knockout of the objective gene affected the expression patterns of other genes, but did not disrupt the circadian rhythm, which may have occurred because the highly conserved domains of the PRR family gene are functionally redundant, resulting in the maintenance of the normal circadian rhythm in the absence of function of any of their genes (Figure 9). We also found that the three object genes exerted basically the same effect on photoperiod sensitivity. This suggested that the feedback inhibition loops where the three objective genes are located may be related to each other, and they are alternately expressed at different times of the day to maintain the endogenous circadian rhythm oscillation (Figures 9, 12). This is similar to the results obtained from research in Arabidopsis and rice (Zhao et al., 2012; Boden et al., 2014; Atamian and Harmer, 2016; Ford et al., 2016; Sakuraba et al., 2016).

This was also confirmed in the analysis of other circadian gene expression patterns. The analysis of the expression patterns of *ZmCCA1, ZmLHY, ZmELF4,* and two other objective genes, also showed a shift in the expression phase in plants overexpressing either objective gene, or in plants with either objective gene knocked out (Figure 13). From the perspective of expression relationships, the expression cycle and phase of *ZmCCA1* and *ZmLHY* are completely consistent, and they form a complex that works together in a feedback inhibition loop. Overexpression of *ZmTOC1* suppressed the expression of *ZmCCA1/ZmLHY* and promoted the expression of *ZmELF4*. This occurred because *ZmTOC1* forms a central feedback loop with *ZmCCA1/ZmLHY*, while *ZmELF4* forms a late feedback loop with *ZmLHY/ZmCCA1*, and *ZmCCA1/ZmLHY* is inhibited by *ZmTOC1* at night, thereby weakening the inhibitory effect of Zm*CCA1/ZmLHY* on *ZmELF4*.

The peak expression of *ZmCCA1/ZmLHY* was at 6:00 am, and it continued to inhibit the expression of *ZmPRR73* and *ZmPRR95*. At 8:00 am, the expression of *ZmPRR95* peaked, which inhibited the expression of *ZmCCA1/ZmLHY* and *ZmPRR73*. The expression of *ZmPRR73* continuously increased between 8:00 am and 10:00 am, and reached a peak at 10:00 am *ZmCCA1/ZmLHY* forms an early feedback loop with *ZmPRR73* and *ZmPRR95*, and interacts with central and late feedback loops to jointly regulate the endogenous circadian rhythm of plants, thus affecting the differentiation of SAMs to enable tasseling and the development and maturation of tassels (Figures 7, 10, 13). The effect of circadian rhythm on photoperiod sensitivity is achieved by changing its oscillatory rhythm in response to external photoperiod changes, thereby regulating the entire developmental process from vegetative to reproductive growth (Figures 8–10; Supplementary Figures S9, S10).

## Conclusion

We used high-density genetic maps to map 39 QTLs related to photoperiod sensitivity, and combined with transcriptome and expression pattern analyses, *ZmTOC1b, ZmPRR93,* and *ZmPRR73* were finally identified. These are key genes in the circadian rhythm pathway that are rhythmically expressed in maize and negatively regulate photoperiod sensitivity. When they are knocked out using CRISPR/Cas9 technology, photoperiod sensitivity was blunted. When stimulated by exogenous photoperiod changes, they can modulate the endogenous circadian rhythm of maize by changing the period, phase, and amplitude of their own expression, thereby affecting maize growth and development, including hypocotyl elongation, shoot apical division and differentiation of living tissue, and differentiation and development of tassels.

The accumulation of these growth and development processes is ultimately reflected by changes in plant architecture and flowering stage. This study lays a foundation for further exploration of the regulatory mechanism of photoperiod sensitivity, and provides new ideas and methods for improving the adaptability of tropical varieties in temperate zones.

## Data Availability Statement

The datasets presented in this study can be found in online repositories. The names of the repository/repositories and accession number(s) can be found at: National Center for Biotechnology Information (NCBI) BioProject database under accession number PRJNA316482.

## Author Contributions

JF and SG: conceptualization. JF: methodology. JL: software. PW, JQ, and SL: validation. JF and MG: investigation. SG and YM: resources. QJ: data curation. JF: writing—original draft preparation and writing—review and editing. SG: project administration. All authors contributed to the article and approved the submitted version.

## Funding

This work was supported by Jilin Province Science and Technology Development Plan Project (20200402023NC) and Major Science and Technology Special Project of Jilin Province Science and Technology Development Plan (20210302003NC).

## Conflict of Interest

The authors declare that the research was conducted in the absence of any commercial or financial relationships that could be construed as a potential conflict of interest.

## Publisher’s Note

All claims expressed in this article are solely those of the authors and do not necessarily represent those of their affiliated organizations, or those of the publisher, the editors and the reviewers. Any product that may be evaluated in this article, or claim that may be made by its manufacturer, is not guaranteed or endorsed by the publisher.

## Footnotes

^1^ https://venyao.xyz/shinyCircos/

^2^ https://www.megasoftware.net/dload_win_gui

^3^ http://pfam.xfam.org/

^4^ https://www.lynnon.com/

^5^ http://bioinformatics.psb.ugent.be/webtools/plantcare/html/

^6^ http://www.premierbiosoft.com/
