# Supplementary material for: Fine Mapping and Functional Research of Key Genes for Photoperiod Sensitivity in Maize
Source: Front Plant Sci. 2022 Jul 12;13:890780. doi: 10.3389/fpls.2022.890780 (PMC9315444; doi:10.3389/fpls.2022.890780)
Supplement: Supplementary file 1 [file Table_1.DOCX]

| **Traits** | **Environment** | **Mean±SD** | **Range** | **CV** | **Skew** | **Kurtosis** | **H^2^** |
| --- | --- | --- | --- | --- | --- | --- | --- |
| PHPS | 2018-HN-C | 87.62±4.21 | -23.44-253.89 | 4.8% | 0.704 | 0.053 | 86.8 |
|  | 2018-HN-G | 77.83±4.42 | -19.38-254.81 | 5.7% | 0.947 | 0.312 | 87.9 |
|  | 2019-HN-C | 95.27±4.52 | -11-290 | 4.7% | 0.795 | 0.124 | 88.4 |
|  | 2019-HN-G | 102.13±4.69 | -16.44-287.5 | 4.6% | 0.670 | -0.153 | 89.1 |
| EHPS | 2018-HN-C | 114.27±5.06 | -18.95-319 | 4.4% | 0.624 | -0.116 | 85.5 |
|  | 2018-HN-G | 117.37±5.1 | -39-376.74 | 4.3% | 0.37 | -0.138 | 85.6 |
|  | 2019-HN-C | 140.65±6.67 | -25-493.75 | 4.7% | 0.755 | 0.469 | 91.1 |
|  | 2019-HN-G | 157.68±7.3 | -33.18-384.12 | 4.6% | 0.145 | -0.955 | 92.5 |
| LNPS | 2018-HN-C | 48.1±2.3 | -18.18-177.69 | 4.7% | 0.585 | 0.687 | 81.1 |
|  | 2018-HN-G | 49.1±2.8 | -100-214.29 | 5.7% | 0.438 | 0.958 | 85.7 |
|  | 2019-HN-C | 66.7±3.03 | -8.33-187.27 | 4.5% | 0.6 | -0.11 | 87.7 |
|  | 2019-HN-G | 52.45±3.24 | -100-187.5 | 6.1% | 0.581 | 0.505 | 89.2 |
| LEPS | 2018-HN-C | 74.93±3.2 | -13.3-216.67 | 4.3% | 0.209 | -0.249 | 89.8 |
|  | 2018-HN-G | 72.89±2.9 | -8.77-150 | 3.9% | -0.053 | -0.95 | 88.1 |
|  | 2019-HN-C | 82.34±4.05 | 28.57-300 | 4.9% | 0.373 | 0.85 | 93.4 |
|  | 2019-HN-G | 79.17±3.7 | -42.86-300 | 4.7% | 0.768 | 0.885 | 92.2 |
| SSPS | 2018-HN-C | 72.7±2.59 | 2.73-217.78 | 3.6% | 0.372 | -0.106 | 85.9 |
|  | 2018-HN-G | 76.2±2.82 | 1.15-218.87 | 3.7% | 0.438 | 0.028 | 87.9 |
|  | 2019-HN-C | 73.8±2.69 | 7.94-153.19 | 3.6% | 0.284 | -0.996 | 86.7 |
|  | 2019-HN-G | 77.7±2.79 | 2.17-184.56 | 3.6% | 0.179 | 0.801 | 87.5 |
| ATPS | 2018-HN-C | 70.6±2.9 | 0-220.75 | 4.1% | 0.687 | 0.284 | 86.1 |
|  | 2018-HN-G | 88.87±4.6 | -38.89-322.81 | 5.1% | 0.69 | 0.255 | 93.6 |
|  | 2019-HN-C | 81.29±3.57 | -13.36-238.78 | 4.4% | 0.474 | -0.328 | 89.9 |
|  | 2019-HN-G | 80.26±3.25 | 1.43-217.12 | 4% | 0.407 | -0.631 | 87.9 |

**TABLE S1. Descriptive statistics and broad-sense heritability for PHPS, EHPS, LNPS, LEPS, SSPS, and ATPS.**
