# Supplementary material for: Fine Mapping and Functional Research of Key Genes for Photoperiod Sensitivity in Maize
Source: Front Plant Sci. 2022 Jul 12;13:890780. doi: 10.3389/fpls.2022.890780 (PMC9315444; doi:10.3389/fpls.2022.890780)
Supplement: Supplementary file 3 [file Table_3.DOCX]

| **Trait** | **Environment** | **QTL** | **Chr** | **Marker interval** | **Position(cM)** | **Position(bp)** | **LOD** | **Add** | **R^2^** |
| --- | --- | --- | --- | --- | --- | --- | --- | --- | --- |
| PHPS | 2018-HN-C | qPHPS1-1 | 2 | Marker1920493-Marker1914755 | 40.5-41.5 | 201395587-203325980 | 15.0118 | -32.1005 | 28.4485 |
|  |  | qPHPS1-2 | 5 | Marker4383266-Marker4383295 | 73.5-74.5 | 143401296-143420171 | 2.5351 | -12.6993 | 4.1791 |
|  | 2018-HN-G | qPHPS2-1 | 2 | Marker1920493-Marker1914755 | 40.5-41.5 | 201395587-203325980 | 17.677 | -34.5201 | 28.6205 |
|  |  | qPHPS2-2 | 3 | Marker2675532-Marker2669948 | 97.5-98.5 | 141234617-142704737 | 3.7173 | -14.2127 | 4.966 |
|  |  | qPHPS2-3 | 8 | Marker6462164-Marker6459695 | 33.5-34.5 | 153646227-154319284 | 3.8013 | -14.4006 | 5.071 |
|  | 2019-HN-C | qPHPS3-1 | 2 | Marker1920493-Marker1914755 | 40.5-41.5 | 201395587-203325980 | 15.0231 | -34.9922 | 28.363 |
|  | 2019-HN-G | qPHPS4-1 | 2 | Marker1920493-Marker1914755 | 40.5-41.5 | 201395587-203325980 | 10.8594 | -31.525 | 21.3602 |
| EHPS | 2018-HN-C | qEHPS1-1 | 2 | Marker1920493-Marker1914755 | 40.5-41.5 | 201395587-203325980 | 17.0242 | -40.0194 | 28.6025 |
|  | 2018-HN-G | qEHPS2-1 | 7 | Marker5135740-Marker5131843 | 129.5-130.5 | 12891159-13512822 | 3.8601 | 17.4129 | 5.5218 |
|  |  | qEHPS2-2 | 8 | Marker6523101-Marker6521132 | 22.5-24.5 | 169041444-169474420 | 2.7903 | -14.7965 | 3.9842 |
|  |  | qEHPS2-3 | 2 | Marker1920493-Marker1914755 | 40.5-41.5 | 201395587-203325980 | 15.9187 | -40.8312 | 30.0191 |
|  | 2019-HN-C | qEHPS3-1 | 2 | Marker1920493-Marker1914755 | 40.5-41.5 | 201395587-203325980 | 16.5433 | -54.4167 | 30.2043 |
|  |  | qEHPS3-2 | 2 | Marker1881772-Marker1876129 | 60.5-61.5 | 185242342-187239163 | 3.1788 | -20.754 | 4.3205 |
|  | 2019-HN-G | qEHPS4-1 | 2 | Marker1920493-Marker1914755 | 40.5-41.5 | 201395587-203325980 | 21.7076 | -58.7392 | 35.9896 |
|  |  | qEHPS4-2 | 10 | Marker7468732-Marker7472381 | 131.5-132 | 63895698-64558739 | 3.7725 | 36.6516 | 5.9516 |
| LEPS | 2018-HN-C | qLEPS1-1 | 9 | Marker7158988-Marker7161191 | 52.5-53.5 | 140844080-141270207 | 14.8821 | -24.687 | 27.7613 |
|  | 2018-HN-G | qLEPS2-1 | 9 | Marker7158988-Marker7161191 | 52.5-53.5 | 140844080-141270207 | 20.6634 | -25.3356 | 35.2391 |
|  | 2019-HN-C | qLEPS3-1 | 9 | Marker7158988-Marker7161191 | 52.5-53.5 | 140844080-141270207 | 12.1038 | -27.5803 | 22.6274 |
|  | 2019-HN-G | qLEPS4-1 | 9 | Marker7158988-Marker7161191 | 52.5-53.5 | 140844080-141270207 | 13.6551 | -27.2003 | 26.4415 |
| LNPS | 2018-HN-C | qLNPS1-1 | 5 | Marker4548099-Marker4549324 | 89.5-91.5 | 193672017-194091953 | 3.8605 | -9.0507 | 8.5713 |
|  |  | qLNPS1-2 | 9 | Marker7190346-Marker7190708 | 25.5-26.5 | 151600783-151805770 | 3.3671 | 8.3842 | 7.4737 |
|  |  | qLNPS2-1 | 7 | Marker5166589-Marker5164943 | 119.5-120.5 | 25770966-24367176 | 3.7173 | 10.3033 | 7.6902 |
|  | 2018-HN-G | qLNPS2-2 | 9 | Marker6612972-Marker6610091 | 153.5-154.5 | 8210080-8955362 | 3.2805 | 9.6654 | 6.7823 |
|  | 2019-HN-C | qLNPS3-1 | 2 | Marker1921631-Marker1922082 | 34.5-36.5 | 203854425-204245758 | 2.8859 | -9.8528 | 5.5318 |
|  |  | qLNPS3-2 | 5 | Marker3864734-Marker3870275 | 30.5-33.5 | 9463443-11298666 | 2.6697 | -9.4228 | 5.1225 |
|  |  | qLNPS3-3 | 5 | Marker4532484-Marker4537561 | 87.5-88.5 | 188722771-190587817 | 8.3267 | -16.966 | 16.5078 |
|  | 2019-HN-G | qLNPS4-1 | 1 | Marker1074273-Marker1079104 | 32.5-33.5 | 282002566-283967061 | 2.5233 | 9.4936 | 3.8922 |
|  |  | qLNPS4-2 | 5 | Marker4532484-Marker4537561 | 87.5-88.5 | 188722771-190587817 | 13.015 | -22.937 | 22.4526 |
| SSPS | 2018-HN-C | qSSPS1-1 | 5 | Marker4532484-Marker4537561 | 87.5-88.5 | 188722771-190587817 | 19.0266 | -21.2836 | 33.503 |
|  |  | qSSPS1-2 | 9 | Marker6972184-Marker6958696 | 91.5-92.5 | 89144963-91650109 | 4.0579 | -8.9146 | 5.9615 |
|  | 2018-HN-G | qSSPS2-1 | 5 | Marker4532484-Marker4537561 | 87.5-88.5 | 188722771-190587817 | 13.6096 | -20.9071 | 26.4007 |
|  | 2019-HN-C | qSSPS3-1 | 5 | Marker4529807-Marker4532484 | 87.5-88.5 | 188722771-190587817 | 14.2607 | -19.6733 | 26.9509 |
|  | 2019-HN-G | qSSPS4-1 | 8 | Marker6254344-Marker6249625 | 81.5-83.5 | 102914995-104153792 | 3.0105 | -8.2676 | 4.8202 |
|  |  | qSSPS4-2 | 5 | Marker4532484-Marker4537561 | 87.5-88.5 | 188722771-190587817 | 12.9233 | -20.2882 | 25.528 |
| ATPS | 2018-HN-C | qATPS1-1 | 1 | Marker159457-Marker158927 | 150.5-152.5 | 48637598-48743156 | 3.3362 | -8.3084 | 4.039 |
|  |  | qATPS1-2 | 9 | Marker7020064-Marker7020057 | 75.5-78.5 | 102973592-102973975 | 3.0986 | 7.9541 | 3.7618 |
|  | 2018-HN-G | qATPS2-1 | 10 | Marker7537633-Marker7537622 | 57.5-58.5 | 79576457-79576781 | 4.068 | -11.9126 | 3.4762 |
|  | 2019-HN-C | qATPS3-1 | 9 | Marker7158988-Marker7161191 | 52.5-53.5 | 140844080-141270207 | 11.3426 | -24.3058 | 22.3275 |
|  | 2019-HN-G | qATPS4-1 | 7 | Marker5711731-Marker5704763 | 54.5-63.5 | 159359710-161573292 | 3.2161 | 7.3341 | 1.4639 |

**TABLE S3: Quantitative trait loci (QTL) PHPS,** **EHPS, LNPS, LEPS, SSPS and ATPS detected in different environments.**
