# Supplementary material for: Fine Mapping and Functional Research of Key Genes for Photoperiod Sensitivity in Maize
Source: Front Plant Sci. 2022 Jul 12;13:890780. doi: 10.3389/fpls.2022.890780 (PMC9315444; doi:10.3389/fpls.2022.890780)
Supplement: Supplementary file 9 [file Table_9.DOCX]

| Elements | Number | Sequence | Function |
| --- | --- | --- | --- |
| A-box | 1 | CCGTCC | cis-acting regulatory element |
| ABRE | 7 | ACGTG/CACGTG | cis-acting element involved in the abscisic acid responsiveness |
| AuxRR-core | 1 | GGTCCAT | cis-acting regulatory element involved in auxin responsiveness |
| GA-motif | 1 | ATAGATAA | part of a light responsive element |
| I-box | 1 | TAGATAACC | part of a light responsive element |
| MBS | 2 | CAACTG | MYB binding site involved in drought-inducibility |
| MRE | 1 | AACCTAA | MYB binding site involved in light responsiveness |
| ATC-motif | 1 | AGTAATCT | part of a conserved DNA module involved in light responsiveness |
| G-Box | 4 | CACGTT/CACGTG | cis-acting regulatory element involved in light responsiveness |
| CAAT-box | 11 | CAAAT/CAAT | common cis-acting element in promoter and enhancer regions |
| CGTCA-motif | 10 | CGTCA/CACGTT | cis-acting regulatory element involved in the MeJA-responsiveness |
| GC-motif | 2 | CCCCCG | cis-acting element involved in low-temperature responsiveness |
| O2-site | 1 | GATGA(C/T)(A/G)TG(A/G) | cis-acting regulatory element involved in zein metabolism regulation |
| P-box | 2 | CCTTTTG | gibberellin-responsive element |
| Sp1 | 2 | GGGCGG | light responsive element |
| TATA-box | 15 | TATAAATA/TATA/ATATAT | core promoter element around -30 of transcription start |
| TCA-element | 1 | TCAGAAGAGG | cis-acting element involved in salicylic acid responsiveness |
| TGA-element | 1 | AACGAC | auxin-responsive element |
| TGACG-motif | 5 | TGACG | cis-acting regulatory element involved in the MeJA-responsiveness |
| circadian | 1 | CAAAGATATC | cis-acting regulatory element involved in circadian control |

Table S10. Cis-acting element prediction in the *Zm*PRR73 promoter
