# Supplementary material for: Fine Mapping and Functional Research of Key Genes for Photoperiod Sensitivity in Maize
Source: Front Plant Sci. 2022 Jul 12;13:890780. doi: 10.3389/fpls.2022.890780 (PMC9315444; doi:10.3389/fpls.2022.890780)
Supplement: Supplementary file 11 [file Table_11.DOCX]

**Table S7.**

**TABLE S7: List of all primers involved.** ‘qRT-PCR’ assay is primer for real-time PCR analysis. ‘Cloning’ is a primer used to clone a gene of objective from the genome. ‘Build pCambia3301-ZmPRR95-3His Vector’ is a primer carrying homology arms for constructing plant expression vectors. ‘Build PET-22b-ZmPRR95 Vector’ is a primer carrying homology arms for constructing prokaryotic expression vectors. ‘Oligo’ is a primer for synthesizing targets and ligating CRISPR/Cas9 vectors.

| **Name of Genes** | **Primer Sequences** | **Use** |
| --- | --- | --- |
| PRR95 S  PRR95AS | TCGTCCTCCTATCAGAGTTGTA  GACTGTGGGTAGAATGGAGATG | qRT-PCR assay |
| PRR73 S  PRR73 AS | CCTCAGTGCTCTCTCTTTCTTC  CATTCCCTATCCCTAACACATCC | qRT-PCR assay |
| TOCb1 S  TOCb1 AS | GTGCTCTCAGAACCTAGTGATG  GGTGCCCGTTTCTTGATTTG | qRT-PCR assay |
| PHYB S  PHYB AS | AGACCCTTAGCTCCCTGATAA  GCCTCCTCATCAACACTCAA | qRT-PCR assay |
| CCA1 S  CCA1 AS | CACAAGGTCTTCTCCCTTTCTT  CTGGGCAATCTCTGTCTTTCT | qRT-PCR assay |
| LHY S  LHY AS | CTGGTGAGGAAACGGTGATAAA  TGCCATGCTCTGCCATAAA | qRT-PCR assay |
| ELF S  ELF AS | GGAGACGGATGCTTGGTTT  AGGGTGGTGCTATTGGTATTG | qRT-PCR assay |
| ActinII1S  ActinII1AS | GCTGTTCTTTCACTTTATGCAAG  CGCTCGGCTGAGGTGGTGAAGGA | Reference gene |
| TOCb1 S  TOCb1 AS | GAATCCGACGCTTCGGTT  TTTCACACAAGAGGACCTCTCC | Clone |
| PRR73 S  PRR73 AS | TTCGCAGTTGGATTCAGCATA  AGATCTGTCGACGAAGATGCG | Clone |
| PRR95 S  PRR95AS | TTCCAACTTCCAAGTGGCGAC  TCCAGCTCAGTAACTGGACCCT | Clone |
| PRR95 S  PRR95AS | actcttgaccatggtaGTAGTAGTAAACTTCCAAGTGGCGAC  ggggaaattcgagctggGTAGTAGTAGCTCAGTAACTGGACCCT | Build pCambia3301-ZmPRR95-3His Vector |
| PRR73 S  PRR73 AS | actcttgaccatggtaGTAGTAGTACAGTTGGATTCAGCATA  ggggaaattcgagctggGTAGTAGTACTGTCGACGAAGATGCG | Build pCambia3301-ZmPRR73-3His Vector |
| TOCb1 S  TOCb1 AS | actcttgaccatggtaGTAGTAGTACCGACGCTTCGGTT  ggggaaattcgagctggGTAGTAGTAACACAAGAGGACCTCTCC | Build pCambia3301-ZmTOC1b-3His Vector |
| PRR95 S  PRR95AS | gtggtggtggtggtgctcgagTTCCAACTTCCAAGTGGCGAC  tcgagctccgtcgacaagcttTCCAGCTCAGTAACTGGACCCT | Build PET-22b-ZmPRR95 Vector |
| PRR73 S  PRR73 AS | gtggtggtggtggtgctcgagTTCGCAGTTGGATTCAGCATA  tcgagctccgtcgacaagcttAGATCTGTCGACGAAGATGCG | Build PET-22b-ZmPRR73 Vector |
| TOCb1 S  TOCb1 AS | gtggtggtggtggtgctcgagGAATCCGACGCTTCGGTT  tcgagctccgtcgacaagcttTTTCACACAAGAGGACCTCTCC | Build PET-22b-ZmTOC1b Vector |
| PRR95-g1- up  PRR95-g1-low | TGTGTGGCGTCCAGCTTAGTAAATGC AAACGCATTTACTAAGCTGGACGCCA | Oligo |
| PRR73-g1- up  PRR73-g1- low | TGTGTGGCCTCAGAACTGTTATCTTG AAACCAAGATAACAGTTCTGAGGCCA | Oligo |
| TOCb1-g1-up  TOCb1-g1-low | TGTGTGGGTTTGGGTATGGCGGGTGG AAACCCACCCGCCATACCCAAACCCA | Oligo |
| PRR95-g2-up  PRR95-g2-low | TGTGTGGATGGTACAACTCTGATAGG AAACCCTATCAGAGTTGTACCATCCA | Oligo |
| PRR73-g2- up  PRR73-g2- low | TGTGTGGATGCTGCTCCAATAAAGCA AAACTGCTTTATTGGAGCAGCATCCA | Oligo |
| TOCb1-g2-up  TOCb1 -g2-low | TGTGTGGCTCCGGTCCACGAACTGCT AAACAGCAGTTCGTGGACCGGAGCCA | Oligo |
