# Supplementary material for: Fine Mapping and Functional Research of Key Genes for Photoperiod Sensitivity in Maize
Source: Front Plant Sci. 2022 Jul 12;13:890780. doi: 10.3389/fpls.2022.890780 (PMC9315444; doi:10.3389/fpls.2022.890780)
Supplement: Supplementary file 12 [file Data_Sheet_1.PDF]

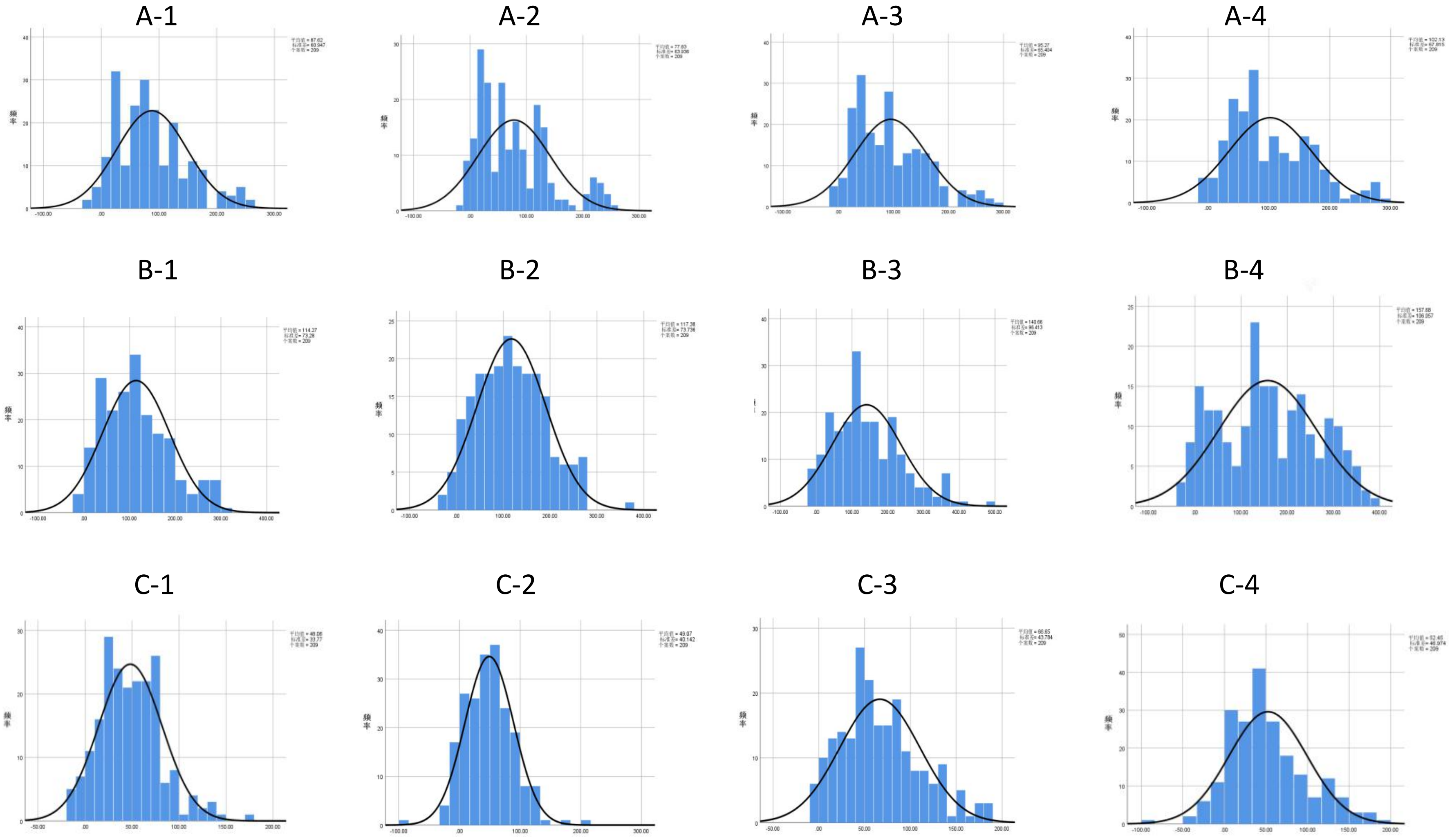
A-1
A-2
A-3
A-4
B-1
B-2
B-3
B-4
C-1
C-2
C-3
C-4
频率

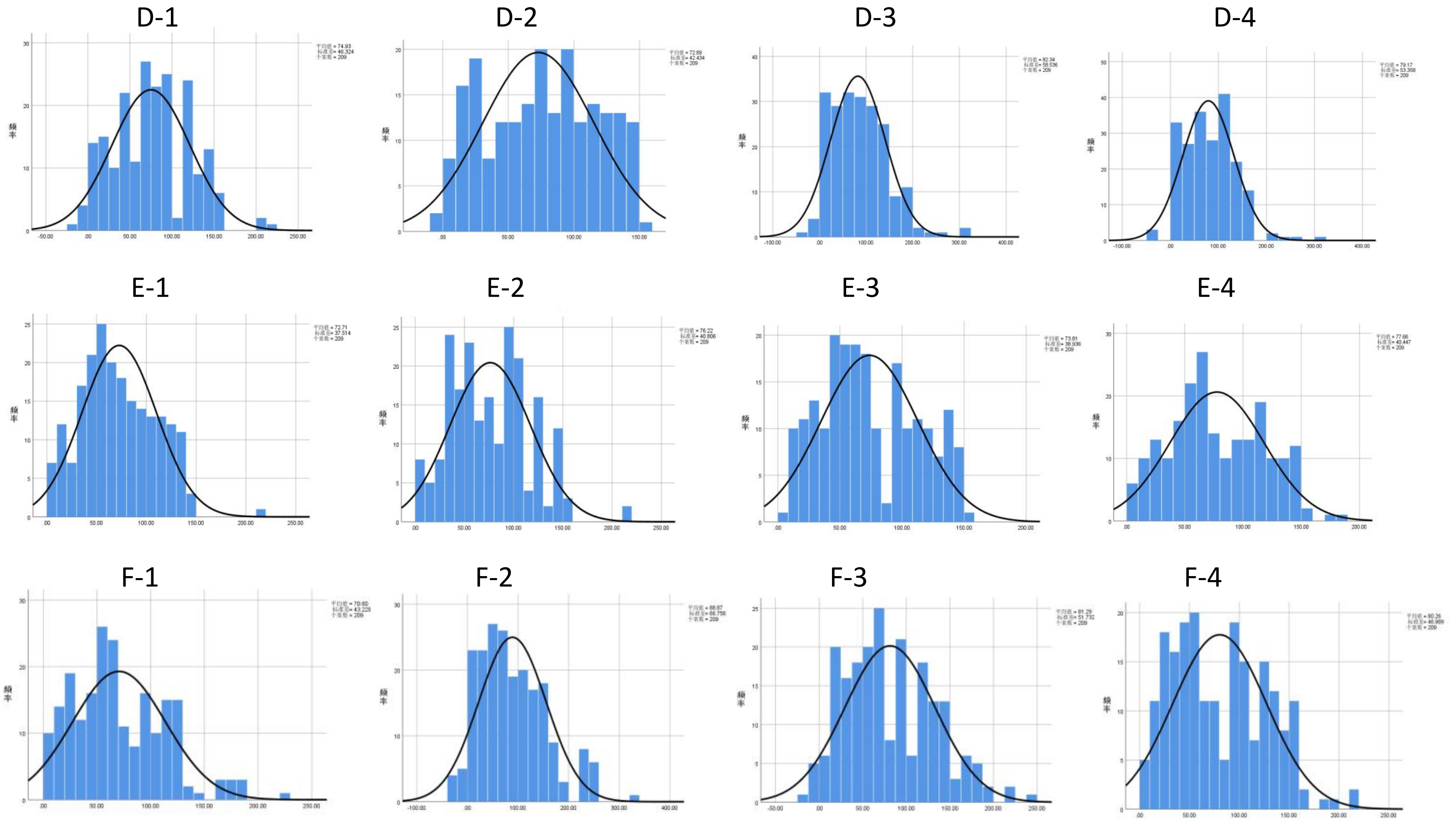

FIGURE S1: Frequency distribution of photoperiod sensitivity of RIL population. The A, B, C, D, E, F represent the six photoperiod-sensitive phenotypes of PHPS, EHPS, LNPS, LEPS, SSPS and ATPS, respectively. The -1, -2, -3 and -4 represent the photoperiod sensitivities between Hainan and Changchun in 2018, photoperiod sensitivities between Hainan and Gongzhuling in 2018, photoperiod sensitivities between Hainan and Changchun in 2019 and photoperiod sensitivities between Hainan and Gongzhuling in 2019, respectively.

A

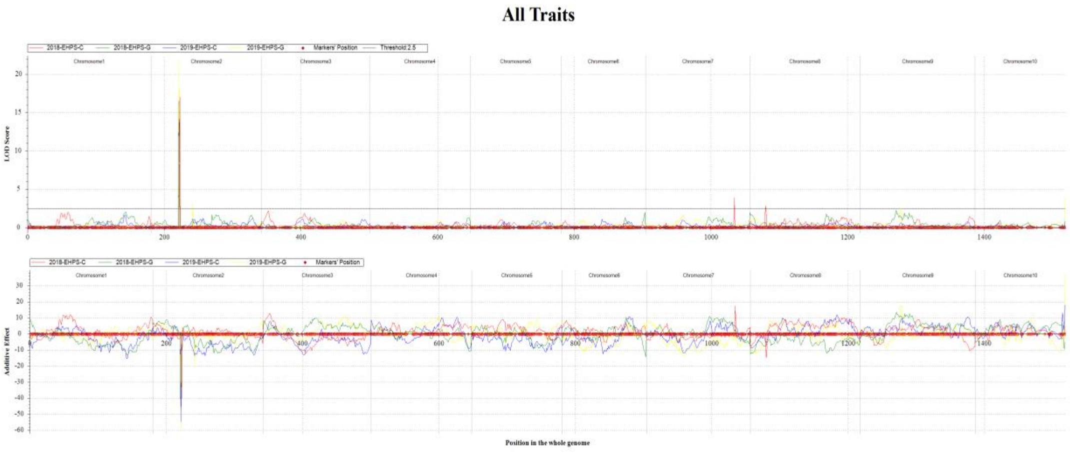

All Traits
2018-EHPS-C
2018-EHPS-G
2019-EHPS-C
2019-EHPS-G
Markers' Position
Threshold:2.5
LOD Score
Additive Effect
Position in the whole genome

B

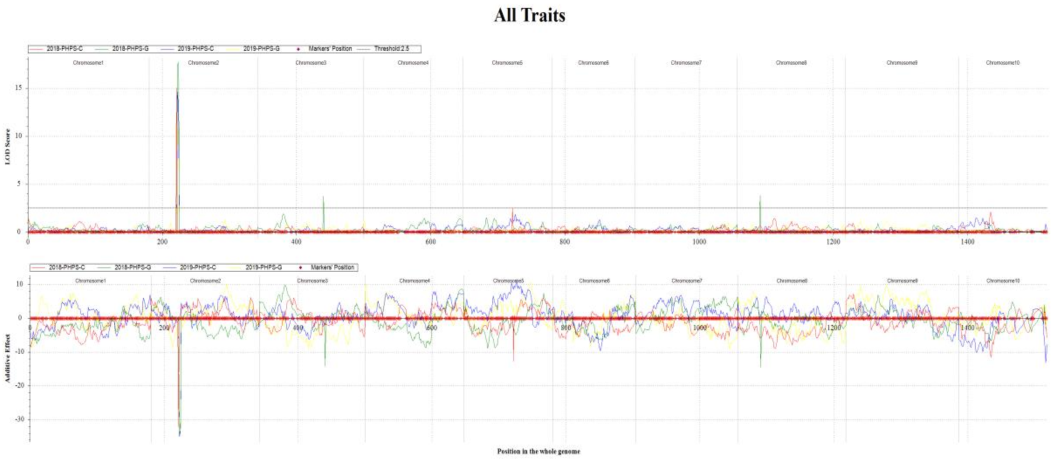

All Traits
2018-PHPS-C
2018-PHPS-G
2019-PHPS-C
2019-PHPS-G
Markers' Position
Threshold:2.5
LOD Score
Additive Effect
Position in the whole genome

C

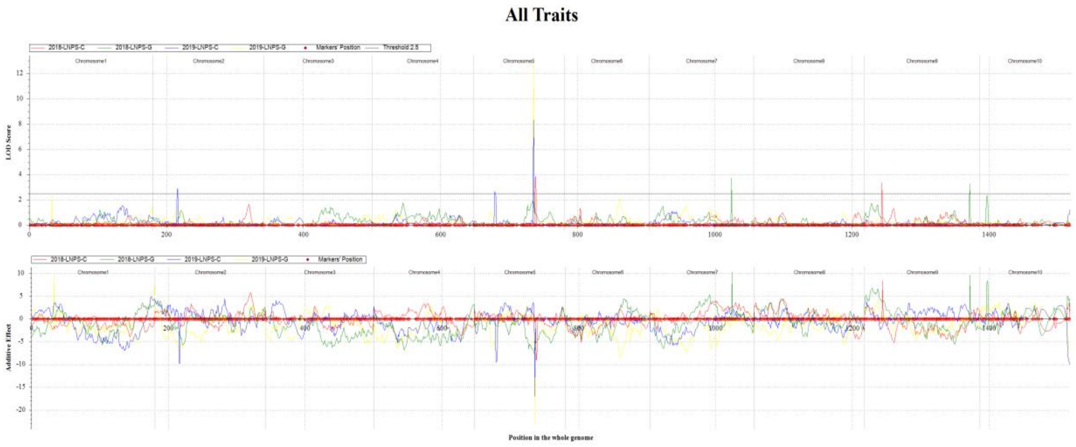

All Traits
2018-LNPS-C
2018-LNPS-G
2019-LNPS-C
2019-LNPS-G
Markers' Position
Threshold:2.5
LOD Score
Additive Effect
Position in the whole genome

D

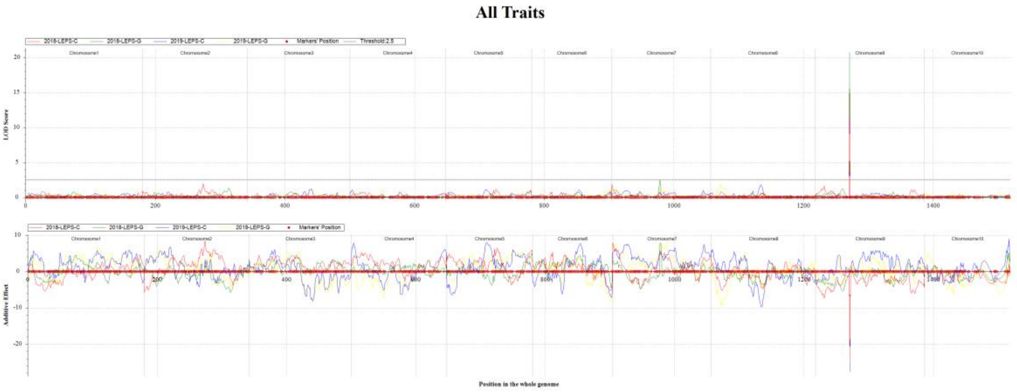

E

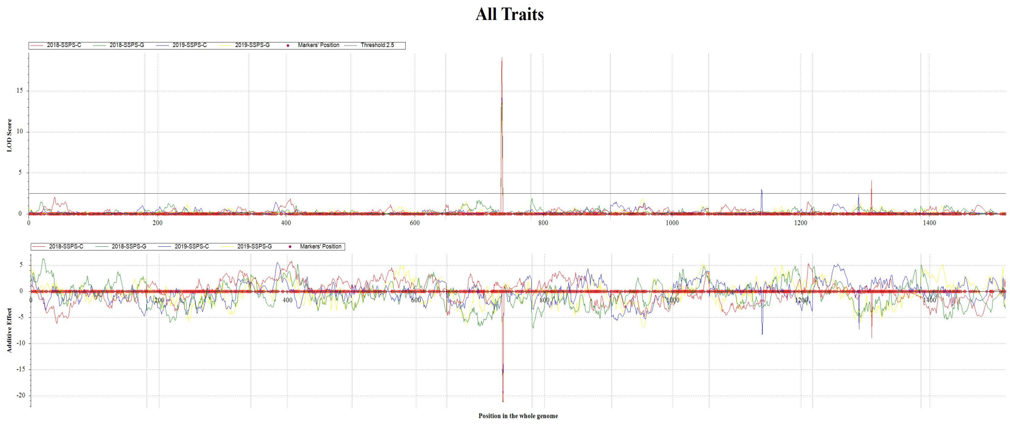

F

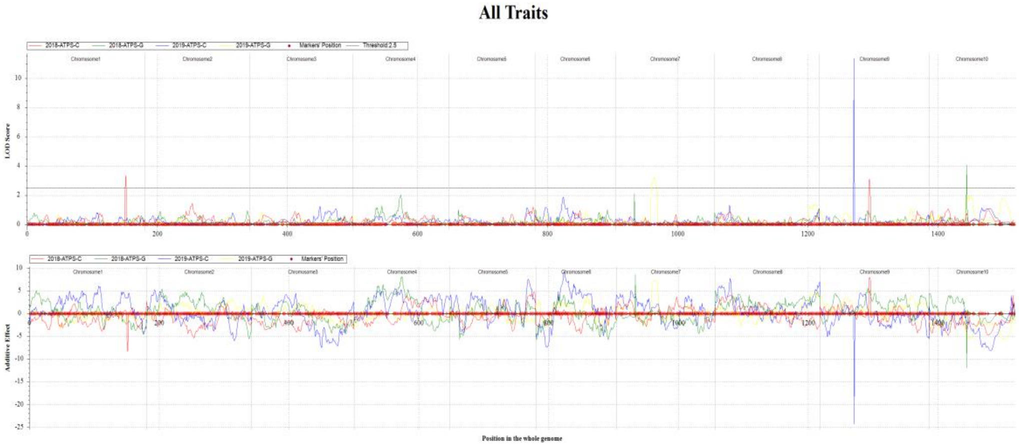

Figure S2: **Quantitative trait loci (*QTL*) for photoperiod sensitivity traits mapped in the RIL population.** The x-axis shows the genetic position along the chromosomes. A vertical bar separates adjacent chromosomes. The y-axis represents the logarithm of the odds (LOD) score of each scanning position. The dotted line represents the LOD significance threshold. Green, red, blue, yellow and dark green represent, 2018 -Hainan-Changchun, 2018 -Hainan-Gongzhuling, 2019 -Hainan-Changchun, and 2019 -Hainan-Gongzhuling, respectively. A, B, C, D, E, and F represent the four photoperiod sensitivity traits of PHPS, EHPS, LNPS, LEPS, SSPS, ATPS ,respectively.

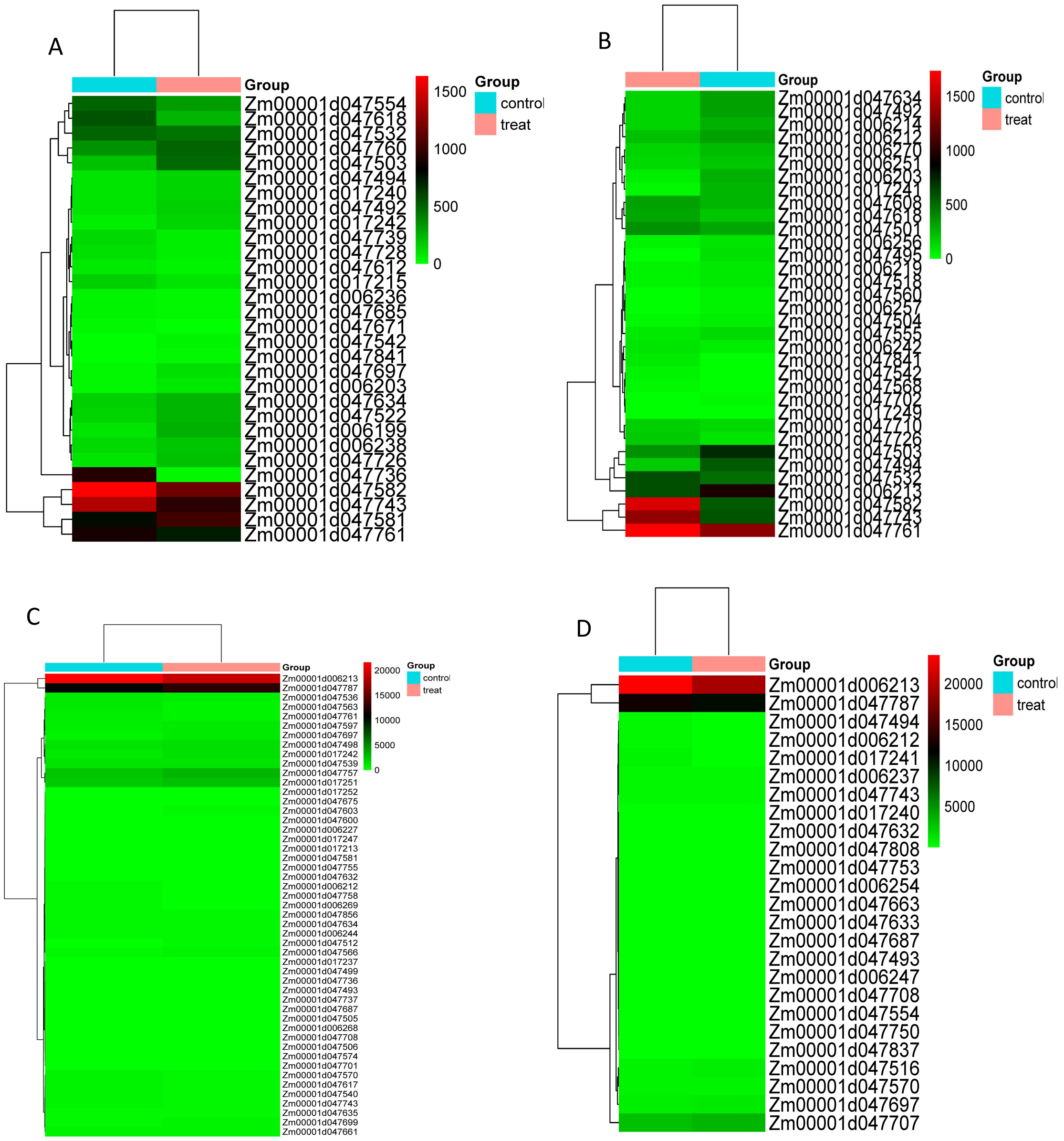

FIGURE S3 Expression heat map of Combined consistent QTL and transcriptome analysis of intersecting genes. (A)Expression heat map of genes that intersect with the transcriptome of the consistent QTL region and the 5-leaf stage leaf. (B) Expression heat map that intersect with the transcriptome of the consistent QTL region and the 6-leaf stage leaf. (C) Expression heat map of genes that intersect with the transcriptome of the consistent QTL region and the 5-leaf stage SAM. (D) Expression heat map of genes that intersect with the transcriptome of the consistent QTL region and the 6-leaf stage SAM.

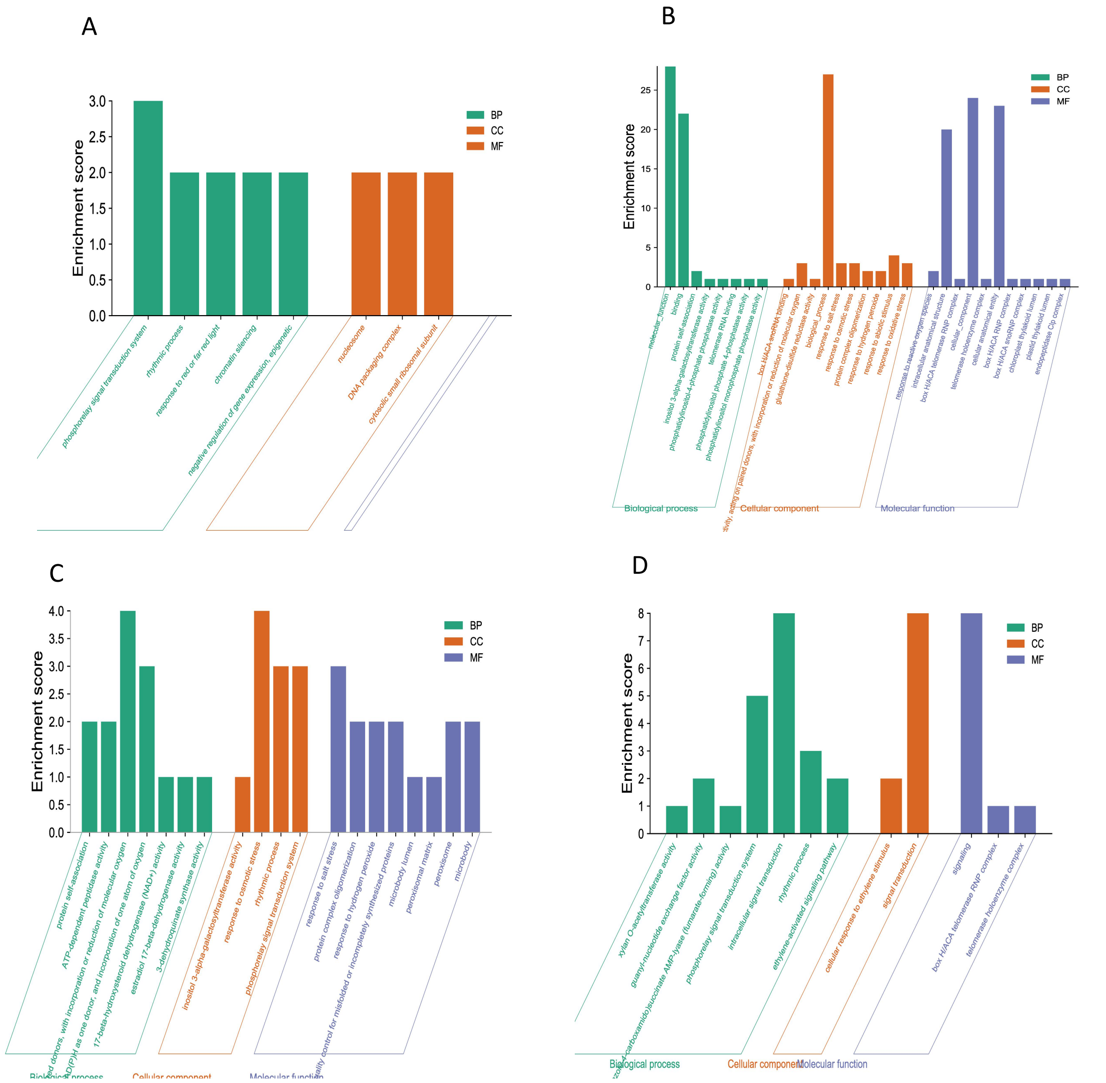

FIGURE S4: GO enrichment of Combined consistent QTL and transcriptome analysis of intersecting genes. (A) GO annotation of genes that intersect with the transcriptome of the consistent QTL region and the 5-leaf stage leaf. (B) GO annotation of genes that intersect with the transcriptome of the consistent QTL region and the 6-leaf stage leaf.(C) GO annotation of genes that intersect with the transcriptome of the consistent QTL region and the 5-leaf stage SAM . (D) GO annotation of genes that intersect with the transcriptome of the consistent QTL region and the 6-leaf stage SAM.

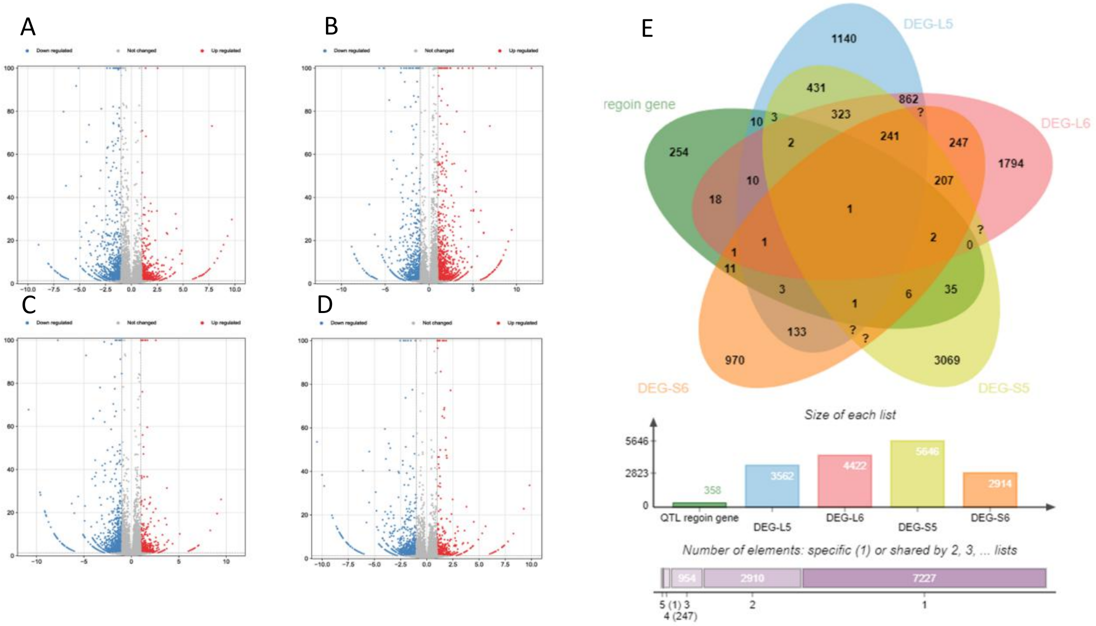

FIGURE S5. Differentially expressed gene volcano plot and joint analysis Venn diagram. (A) Volcano plot of differentially expressed genes in leaves at the 5-leaf stage. (B) Volcano plot of differentially expressed genes in leaves at the 6-leaf stage. (C) Volcano plot of differentially expressed genes in SAMs at the 5-leaf stage. (D) Volcano plot of differentially expressed genes in SAMs at the 6-leaf stage. In the figure, blue represents downregulated genes, and red represents upregulated genes. (E) Venn diagram of consensus QTL region and transcriptome combined analysis. Different colors represent different gene sets, with intersections also shown.

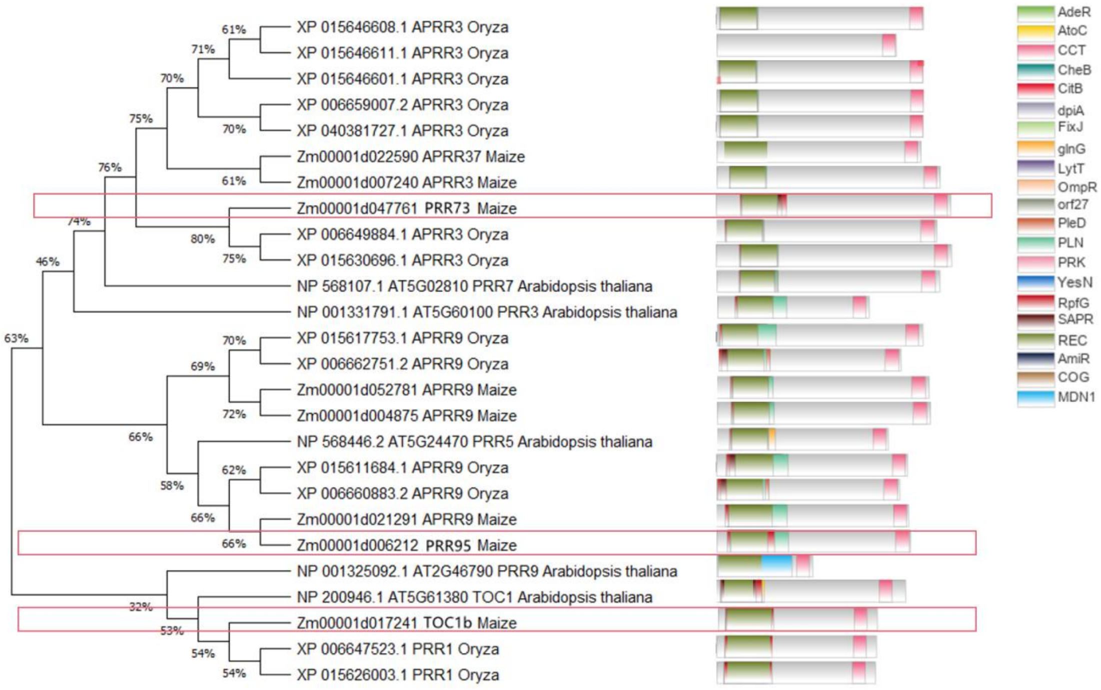

FIGURE S6. Phylogenetic analysis of PRR family proteins in maize, Arabidopsis, and rice. The objective gene is in the red box. Conserved domains of the protein are colored with annotated names.

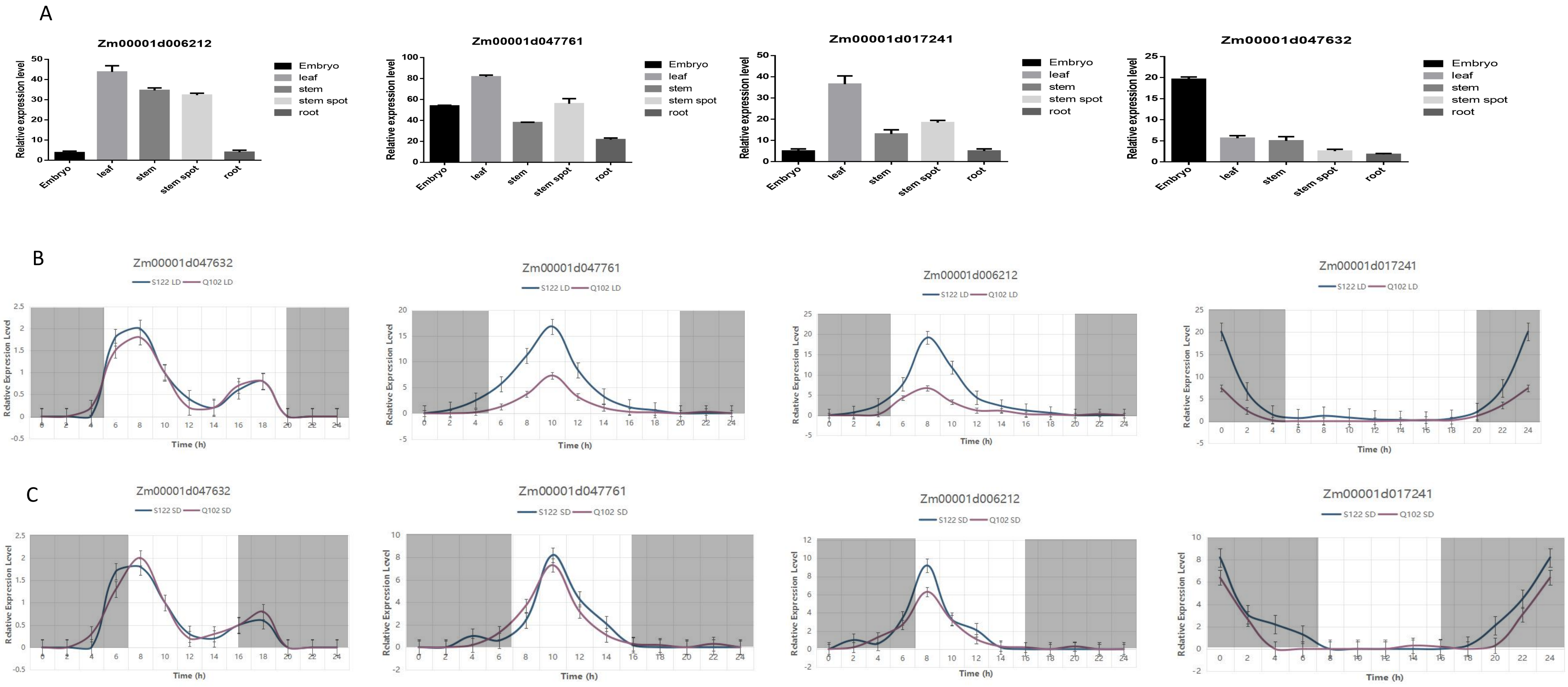

FIGURE S7: Candidate gene expression pattern analysis. (A) Expression levels of candidate genes in different tissue sites. (B) Analysis of expression patterns of candidate genes under LD for 24h consecutive. (C) Analysis of expression patterns of candidate genes under SD for 24h consecutive.

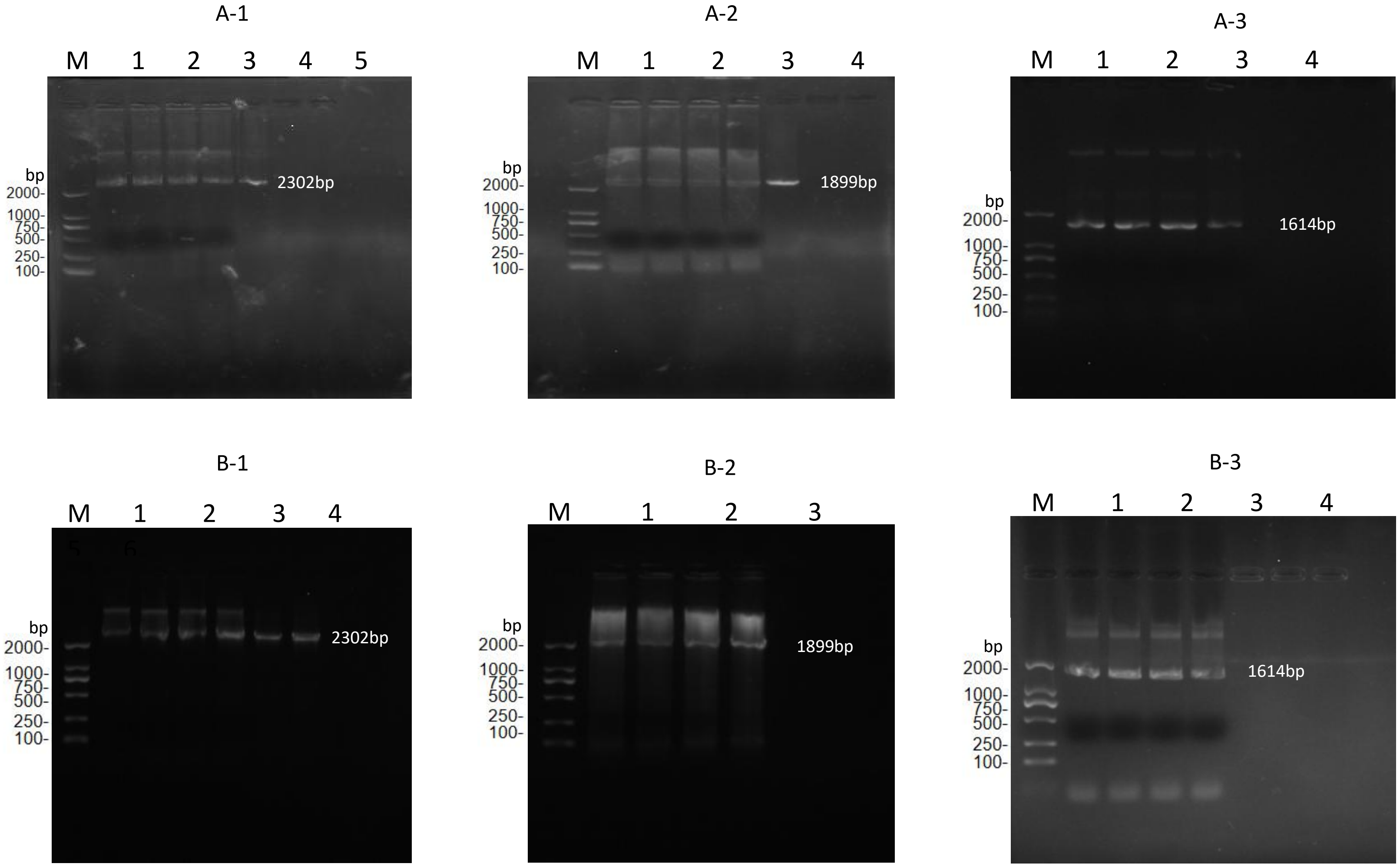
A-1
M 1 2 3 4 5
bp
2000-
1000-
750-
500-
250-
100-
2302bp
A-2
M 1 2 3 4
bp
2000-
1000-
750-
500-
250-
100-
1899bp
A-3
M 1 2 3 4
bp
2000-
1000-
750-
500-
250-
100-
1614bp
B-1
M 1 2 3 4
bp
2000-
1000-
750-
500-
250-
100-
2302bp
B-2
M 1 2 3
bp
2000-
1000-
750-
500-
250-
100-
1899bp
B-3
M 1 2 3 4
bp
2000-
1000-
750-
500-
250-
100-
1614bp

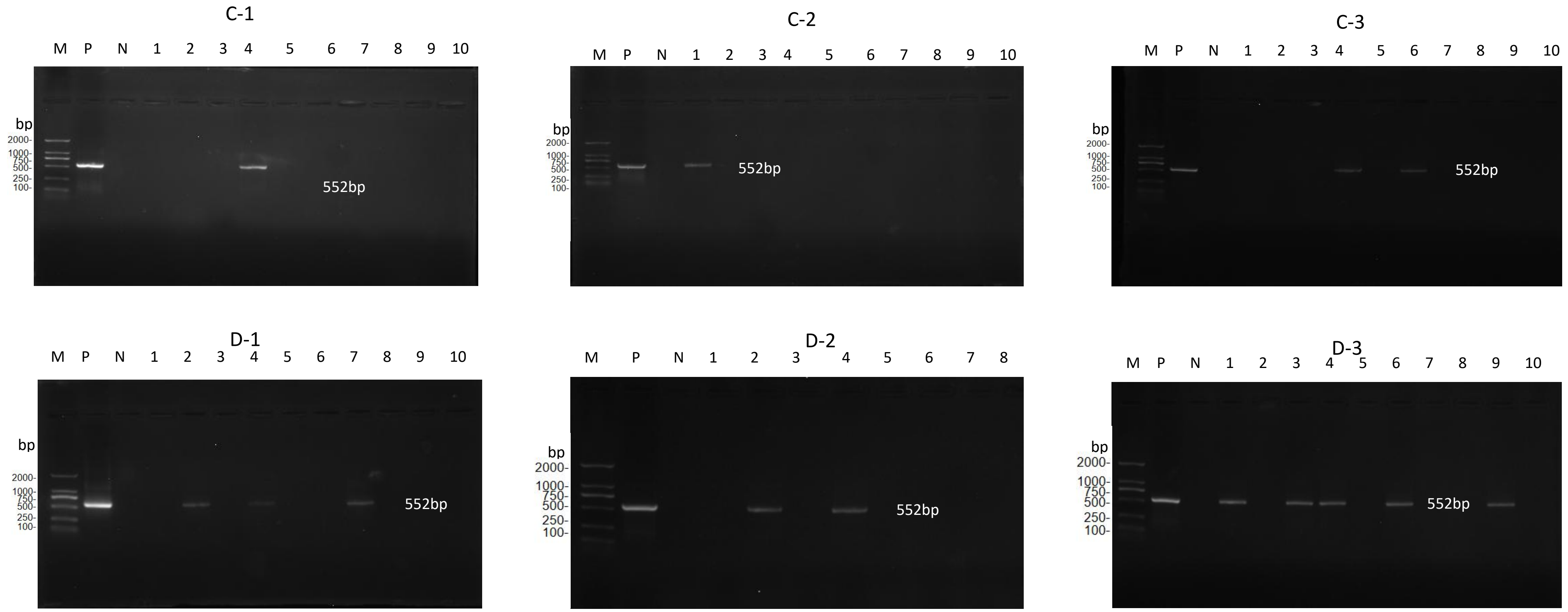
C-1
M P N 1 2 3 4 5 6 7 8 9 10
bp
2000-
1000-
750-
500-
250-
100-
552bp
C-2
M P N 1 2 3 4 5 6 7 8 9 10
bp
2000-
1000-
750-
500-
250-
100-
552bp
C-3
M P N 1 2 3 4 5 6 7 8 9 10
bp
2000-
1000-
750-
500-
250-
100-
552bp
D-1
M P N 1 2 3 4 5 6 7 8 9 10
bp
2000-
1000-
750-
500-
250-
100-
552bp
D-2
M P N 1 2 3 4 5 6 7 8
bp
2000-
1000-
750-
500-
250-
100-
552bp
D-3
M P N 1 2 3 4 5 6 7 8 9 10
bp
2000-
1000-
750-
500-
250-
100-
552bp

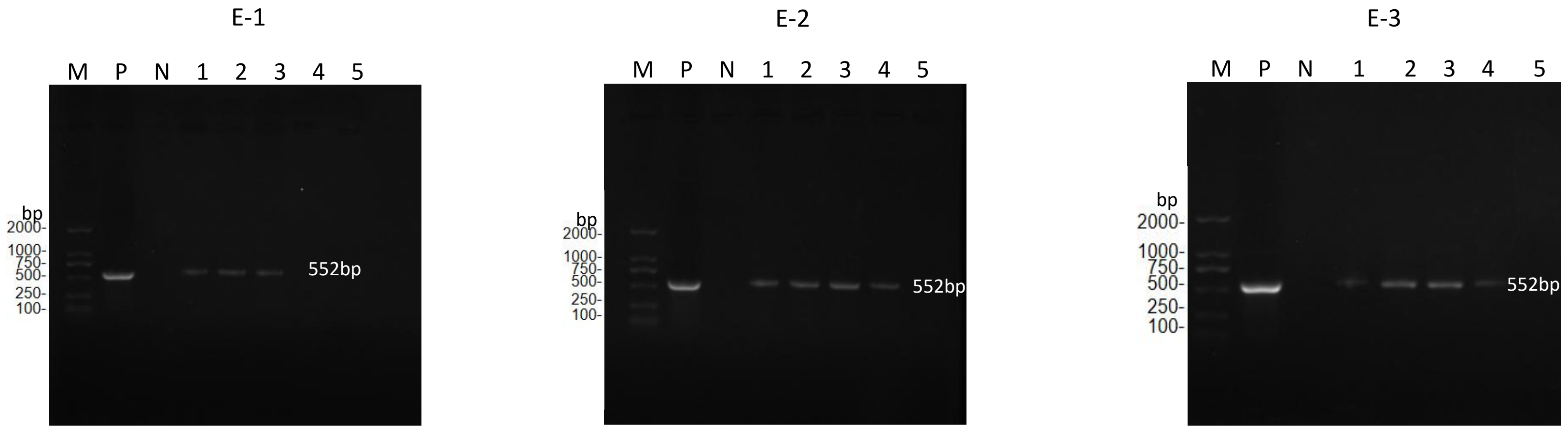

FIGURE S8 : Vector construction and positive plant detection agarose electrophoresis. A, B, C, D and E represent the objective gene pCambia3301 vector double-enzyme digestion electrophoresis image, objective gene PET-22b double-enzyme digestion electrophoresis image, T0 generation overexpressing the objective gene positive plants bar screening marker detection, T0 generation knockout objective gene positive Plant bar selection marker detection and T2 generation overexpression objective gene positive plants selection marker detection, respectively. 1, 2 and 3 represent ZmPRR95, ZmPRR73, ZmPRRTOC1, respectively. M:marker. P: the PCR product using the vector as the substrate was used as a positive control. N: the PCR product using WT as the substrate was used as a negative control.

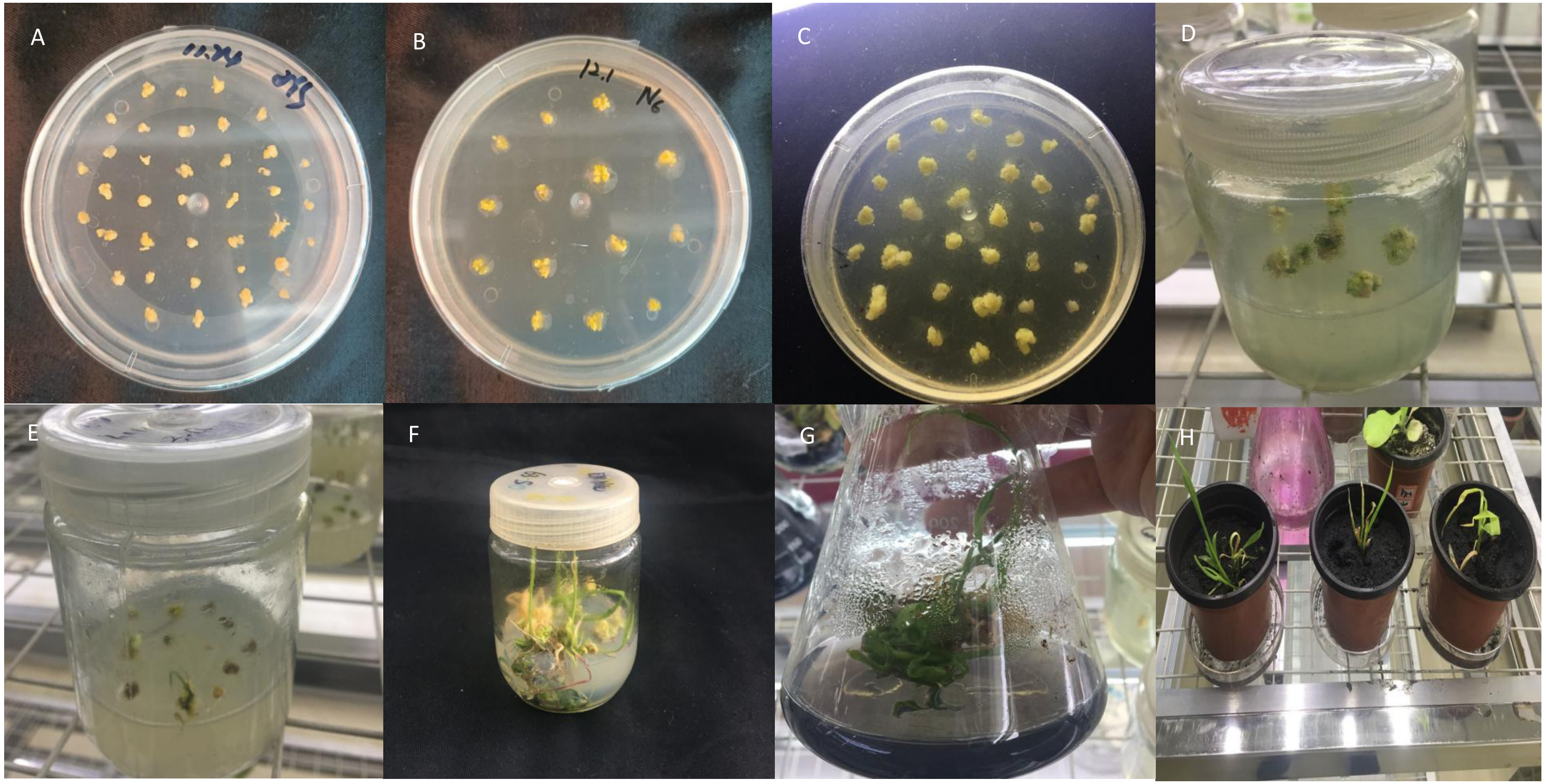

FIGURE S9: Maize genetic transformation.(A) Dedifferentiation of young maize embryos into callus. (B) Maize callus subculture.(C) Co-cultivation of Agrobacterium after infection of callus.(D) Positive screening for herbicide resistance. (E,F) callus differentiation culture.(G) rooting culture.（H）Tissue culture positive seedling transplanting.

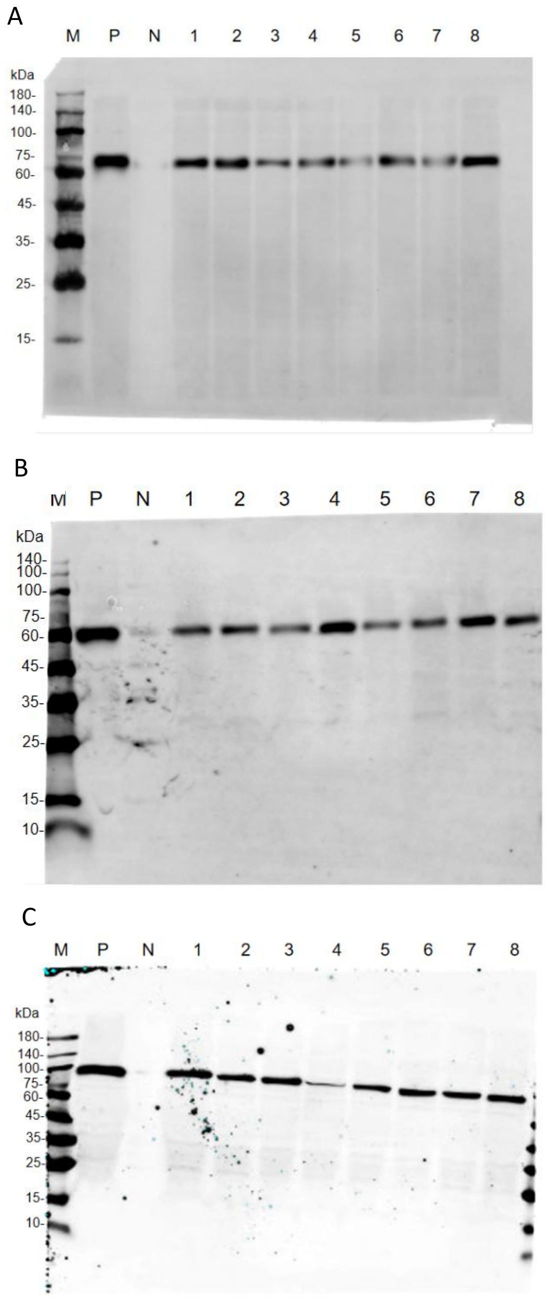

FIGURE S10: Objective gene overexpression T2 generation positive plants were detected by westren-blot. (A) ZmPRR95 overexpression T2 generation positive plants were detected by western-blot. (B) ZmTOC1b overexpression T2 generation positive plants were detected by western-blot. (C) ZmPRR73 overexpression T2 generation positive plants were detected by western-blot. M stands for PM2510 protein marker, P is the prokaryotic expression protein of the objective gene as a positive control,N is the negative control of total plant protein extracted from WT. 1-8 are the total protein of T2 generation transgenic positive plants.

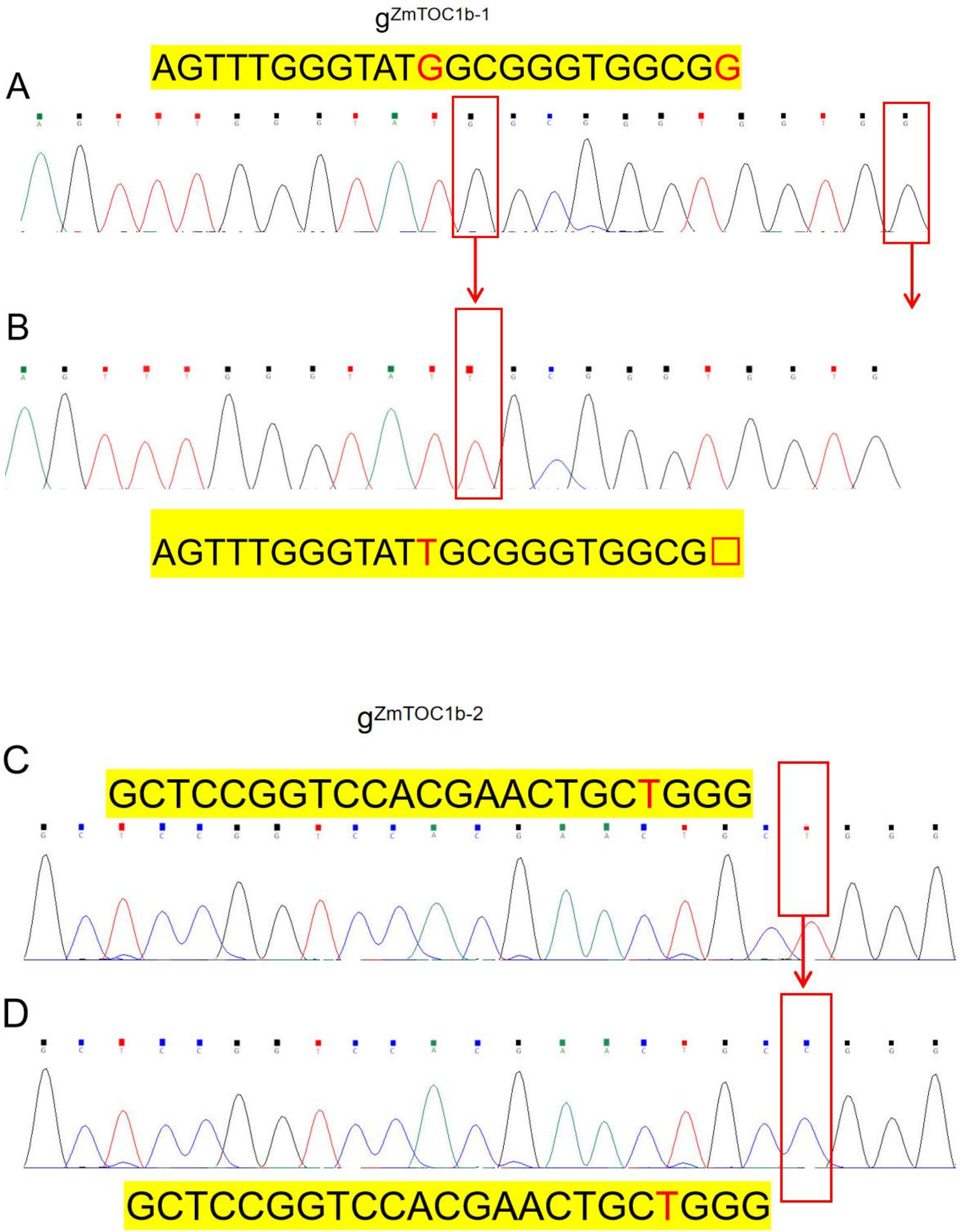
$g^{ZmTOC1b-1}$
A
AGTTTGGGTATGGCGGGTGGCGG
B
AGTTTGGGTATTGCGGGTGGCG
$g^{ZmTOC1b-2}$
C
GCTCCGGTCCACGAACTGCTGGG
D
GCTCCGGTCCACGAACTGCTGGG

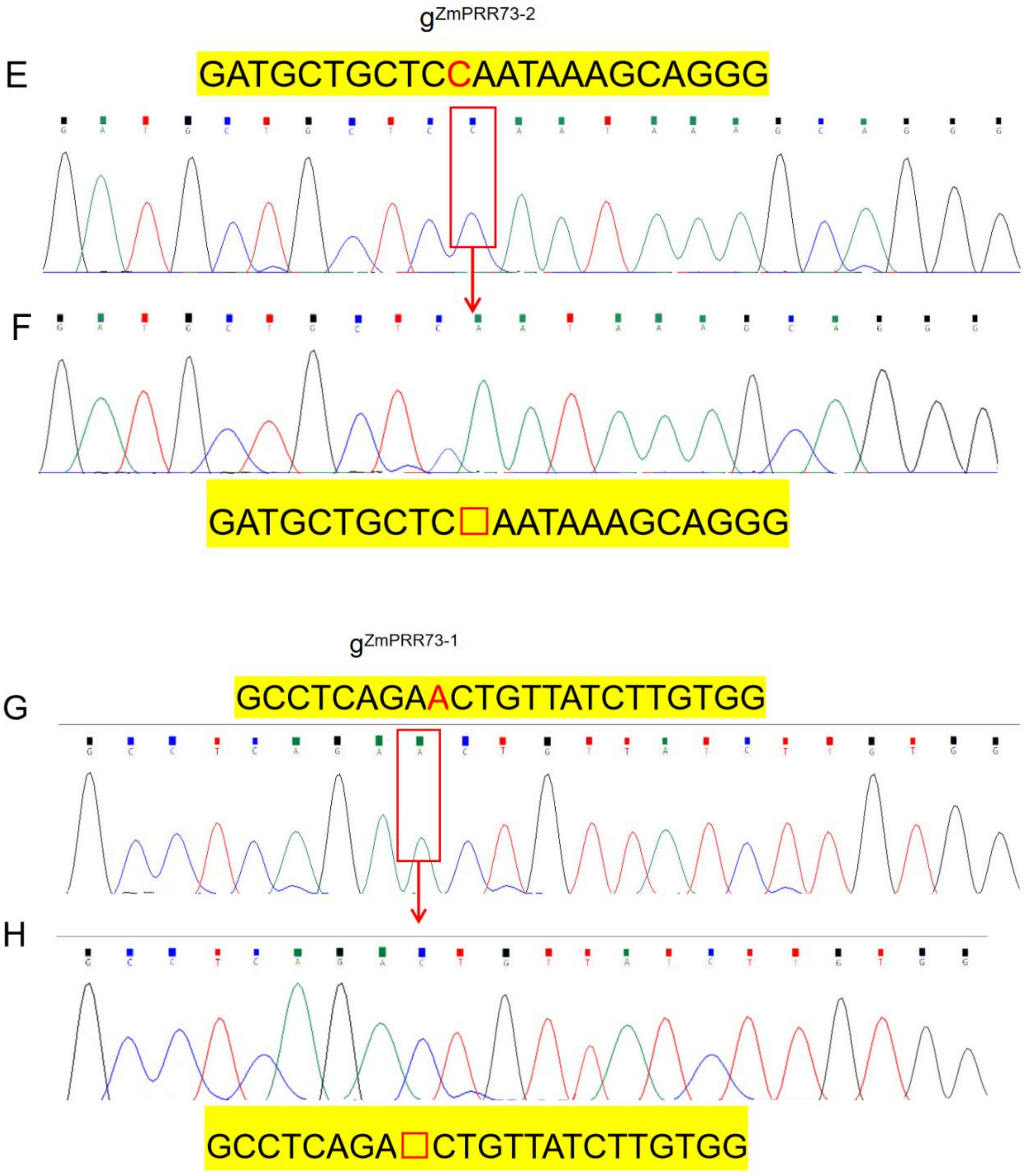
$g^{ZmPRR73-2}$
E
GATGCTGCTCCAATAAAGCAGGG
F
GATGCTGCTC□AATAAAGCAGGG
$g^{ZmPRR73-1}$
G
GCCTCAGAACTGTTATCTTGTGG
H
GCCTCAGA□CTGTTATCTTGTGG

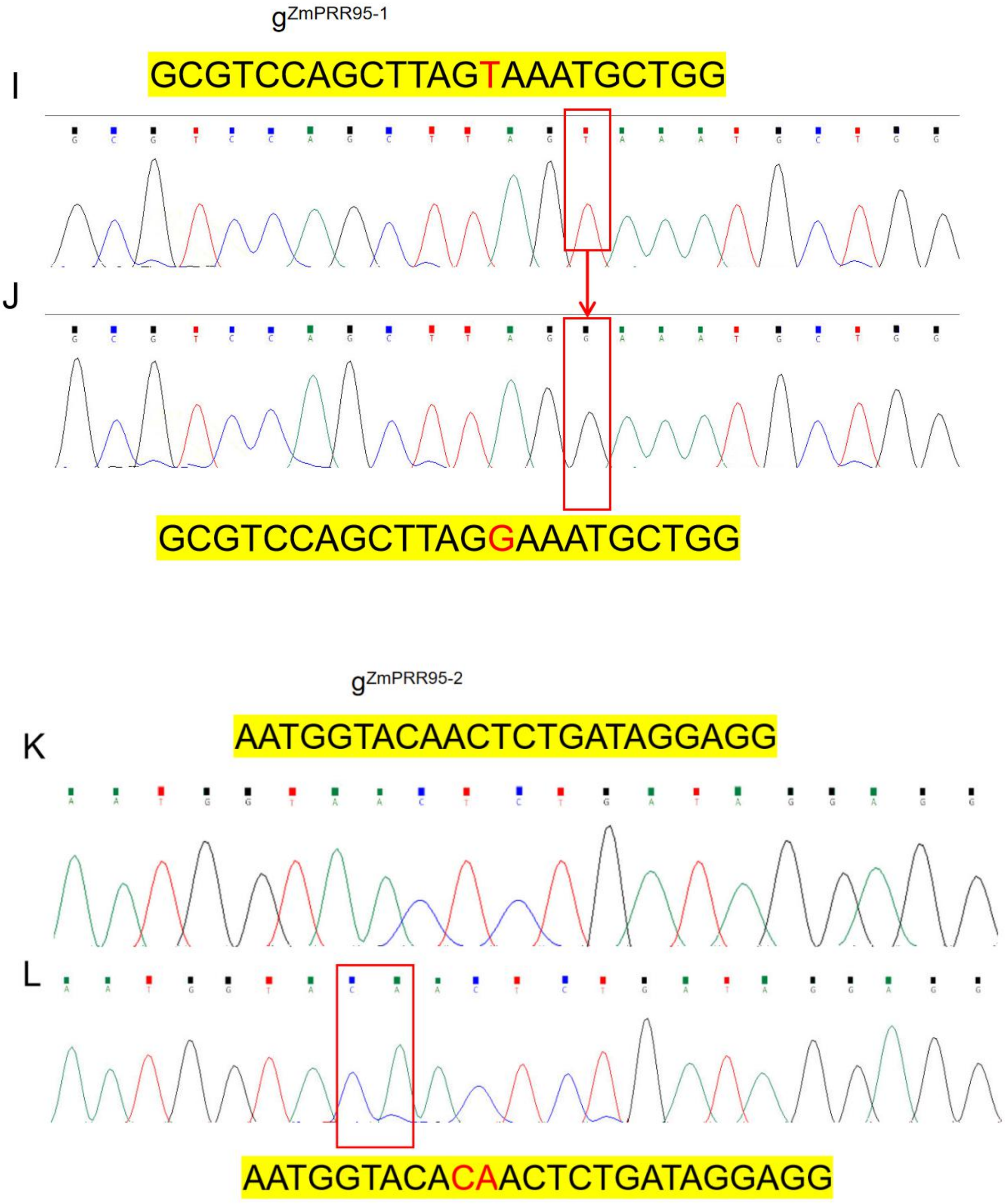

FIGURE S11:Analyses of sequencing chromatogram data of the target mutant sites of objective genes. A and C are wild-type sequencing chromatogram data of two targets of ZmTOC1b. B and D are mutant-type sequencing chromatogram data of two targets of ZmTOC1b. E and G are wild-type sequencing chromatogram data of two targets of ZmPRR73. F and H are mutant-type sequencing chromatogram data of two targets of ZmPRR73. I and K are wild-type sequencing chromatogram data of two targets of ZmPRR95. J and L are mutant-type sequencing chromatogram data of two targets of ZmPRR95. The red box represents the mutation position, and the red arrow represents the mutation direction. The red part of the target sequence marked is the mutated base, and the red box is the missing base.

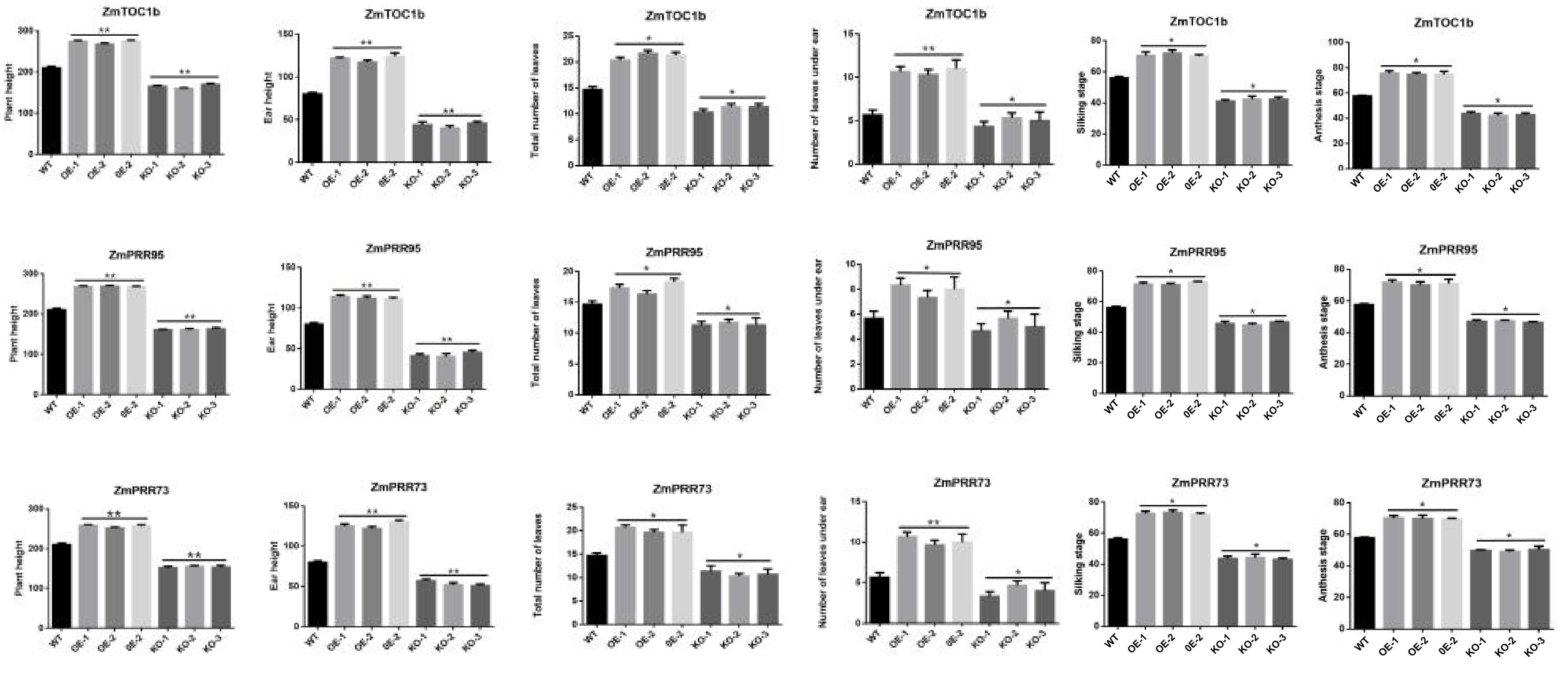

FIGURE S12. Comparison of maize PH, EH, LN, LE, SS, and AT of transgenic-positive plants. The asterisks (*or **) indicate the significant differences at $p < 0.05$ or $p < 0.01$, respectively

A

```
PRR95-Q102.seq   ACAAACTGCCTTTTCTTCCGGCTTCCAACTTCCAAGTGGC   40
PRR95-S122.seq   ACAAACTGCCTTTTCTTCCGGCTTCCAACTTCCAAGTGGC   40
Consensus        acaaactgccttttcttccggcttccaacttccaagtggc

PRR95-Q102.seq   GACTGCTGCCGACTGCCGGGTGCGAGAAGAGGGAGAGATC   80
PRR95-S122.seq   GACTGCTGCCGACTGCCGGGTGCGAGAAGAGGGAGAGATC   80
Consensus        gactgctgccgactgccgggtgcgagaagagggagagatc

PRR95-Q102.seq   AGAGTATCAGACAAGCTGTTGTCCGAATCCACTCCGGCGA   120
PRR95-S122.seq   AGAGTATCAGACAAGCTGTTGTCCGAATCCACTCCGGCGA   120
Consensus        agagtatcagacaagctgttgtccgaatccactccggcga

PRR95-Q102.seq   GGACGAGTGCTGAGGTCGAAATGGGGAGAGGAGCGGATGA   160
PRR95-S122.seq   GGACGAGTGCTGAGGTCGAAATGGGGAGAGGAGCGGATGA   160
Consensus        ggacgagtgctgaggtcgaaatggggagaggagcggatga

PRR95-Q102.seq   GGTGGTGAAGGTCGTGGATTCGGAGGACGGCGAGGGGGAG   200
PRR95-S122.seq   GGTGGTGAAGGTCGTGGATTCGGAGGACGGCGAGGGGGAG   200
Consensus        ggtggtgaaggtcgtggattcggaggacggcgagggggag

PRR95-Q102.seq   GAGGAAGAGGCAGAGGCCGCGGCGGCGGAGGGGTCGAGCA   240
PRR95-S122.seq   GAGGAAGAGGCAGAGGCCGCGGCGGCGGAGGGGTCGAGCA   240
Consensus        gaggaagaggcagaggccgcggcggcggaggggtcgagca

PRR95-Q102.seq   AGGAGACGCCGATGCTGCCAAGGATGCCGGTGCGCGTGCT   280
PRR95-S122.seq   AGGAGACGCCGATGCTGCCAAGGATGCCGGTGCGCGTGCT   280
Consensus        aggagacgccgatgctgccaaggatgccggtgcgcgtgct

PRR95-Q102.seq   ACTCGCCGAGGGCGACGACTCCACGCGCCACGTCATCTCC   320
PRR95-S122.seq   ACTCGCCGAGGGCGACGACTCCACGCGCCACGTCATCTCC   320
Consensus        actcgccgagggcgacgactccacgcgccacgtcatctcc

PRR95-Q102.seq   GCGCTGCTACGCAAGTGCGGCTACCGAGTTGCTGCAGCCT   360
PRR95-S122.seq   GCGCTGCTACGCAAGTGCGGCTACCGAGTTGCTGCAGCCT   360
Consensus        gcgctgctacgcaagtgcggctaccgagttgctgcagcct

PRR95-Q102.seq   CTGATGGTGTGAAGGCGTGGGACATATTAAAGGAAAAATC   400
PRR95-S122.seq   CTGATGGTGTGAAGGCGTGGGACATATTAAAGGAAAAATC   400
Consensus        ctgatggtgtgaaggcgtgggacatattaaaggaaaaatc

PRR95-Q102.seq   TTTCAACATAGACCTTGTTTTAACTGAAGTTGAACTGCCT   440
PRR95-S122.seq   TTTCAACATAGACCTTGTTTTAACTGAAGTTGAACTGCCT   440
Consensus        tttcaacatagaccttgttttaactgaagttgaactgcct

PRR95-Q102.seq   TTGATGTCTGGGTTCCTCTTGTTATCCACGATCATGGAGC   480
PRR95-S122.seq   TTGATGTCTGGGTTCCTCTTGTTATCCACGATCATGGAGC   480
Consensus        ttgatgtctgggttcctcttgttatccacgatcatggagc

PRR95-Q102.seq   ATGATGCATCCAAGAACATCCCTGTTATAATGATGTCTTC   520
PRR95-S122.seq   ATGATGCATCCAAGAACATCCCTGTTATAATGATGTCTTC   520
Consensus        atgatgcatccaagaacatccctgttataatgatgtcttc

PRR95-Q102.seq   ACACGACTCTGTAAGCATGGTTTTCAAATGCATGCTAAAG   560
PRR95-S122.seq   ACACGACTCTGTAAGCATGGTTTTCAAATGCATGCTAAAG   560
Consensus        acacgactctgtaagcatggttttcaaatgcatgctaaag

PRR95-Q102.seq   GGTGCATCAGATTTCCTTGTTAAGCCGCTAAGAAAGAACG   600
PRR95-S122.seq   GGTGCATCAGATTTCCTTGTTAAGCCGCTAAGAAAGAACG   600
Consensus        ggtgcatcagatttccttgttaagccgctaagaaagaacg

PRR95-Q102.seq   AATTAAGGAACTTGTGGCAGCACGTTTGGAGAAAACAACT   640
PRR95-S122.seq   AATTAAGGAACTTGTGGCAGCACGTTTGGAGAAAACAACT   640
Consensus        aattaaggaacttgtggcagcacgtttggagaaaacaact

PRR95-Q102.seq   GGCAAACGGTGGGCCCGATGTGCAGCACATACAAGAAGAG   680
PRR95-S122.seq   GGCAAACGGTGGGCCCGATGTGCAGCACATACAAGAAGAG   680
Consensus        ggcaaacggtgggcccgatgtgcagcacatacaagaagag

PRR95-Q102.seq   AATCTTGCAGAAAGAATGGAACAGAAGACTGGCGTGACAA   720
PRR95-S122.seq   AATCTTGCAGAAAGAATGGAACAGAAGACTGGCGTGACAA   720
Consensus        aatcttgcagaaagaatggaacagaagactggcgtgacaa

PRR95-Q102.seq   AAGATGATAATTTGAACAGCGATGGTCCCTGTAAAAATAG   760
PRR95-S122.seq   AAGATGATAATTTGAACAGCGATGGTCCCTGTAAAAATAG   760
Consensus        aagatgataatttgaacagcgatggtccctgtaaaaatag

PRR95-Q102.seq   AGAATGCAGTGAACAAGAAAGTGATGCTCAAAGTTCTTGC   800
PRR95-S122.seq   AGAATGCAGTGAACAAGAAAGTGATGCTCAAAGTTCTTGC   800
Consensus        agaatgcagtgaacaagaaagtgatgctcaaagttcttgc

PRR95-Q102.seq   ACAAGGTCAGAGTTGGAGGCTGAAAGTAAGCAAACTAACA   840
PRR95-S122.seq   ACAAGGTCAGAGTTGGAGGCTGAAAGTAAGCAAACTAACA   840
Consensus        acaaggtcagagttggaggctgaaagtaagcaaactaaca

PRR95-Q102.seq   ACATTTTGGAGTATATGCAATCAACTGAAAGGCATTTGTC   880
PRR95-S122.seq   ACATTTTGGAGTATATGCAATCAACTGAAAGGCATTTGTC   880
Consensus        acattttggagtatatgcaatcaactgaaaggcatttgtc

PRR95-Q102.seq   TATTCGTAGCCACAAGGATTTAGAGCTAAATGGAGAGACC   920
PRR95-S122.seq   TATTCGTAGCCACAAGGATTTAGAGCTAAATGGAGAGACC   920
Consensus        tattcgtagccacaaggatttagagctaaatggagagacc

PRR95-Q102.seq   AAAACACGAACTAAGGGTAATAACTTGATTCCAACAAGAG   960
PRR95-S122.seq   AAAACACGAACTAAGGGTAATAACTTGATTCCAACAAGAG   960
Consensus        aaaacacgaactaagggtaataacttgattccaacaagag

PRR95-Q102.seq   AAGACGATTTATTGCCAAAGAAAAGAACATGTTTGAATGA   1000
PRR95-S122.seq   AAGACGATTTATTGCCAAAGAAAAGAACATGTTTGAATGA   1000
Consensus        aagacgatttattgccaaagaaaagaacatgtttgaatga

PRR95-Q102.seq   CAATAATTCTGAGAGAACTTCCAGAGATATGGAGCTAGTC   1040
PRR95-S122.seq   CAATAATTCTGAGAGAACTTCCAGAGATATGGAGCTAGTC   1040
Consensus        caataattctgagagaacttccagagatatggagctagtc

PRR95-Q102.seq   CACATTATGGACAATCAGCAGAAGCATGACACACAGAGGG   1080
PRR95-S122.seq   CACATTATGGACAATCAGCAGAAGCATGACACACAGAGGG   1080
Consensus        cacattatggacaatcagcagaagcatgacacacagaggg

PRR95-Q102.seq   ATGTGGATACTATGAGAACAACATCCAGGAGAAATGATGA   1120
PRR95-S122.seq   ATGTGGATACTATGAGAACAACATCCAGGAGAAATGATGA   1120
Consensus        atgtggatactatgagaacaacatccaggagaaatgatga

PRR95-Q102.seq   GAAGAACTCCATCCCAGCACACCAGTTGGAACTTTCTCTT   1160
PRR95-S122.seq   GAAGAACTCCATCCCAGCACACCAGTTGGAACTTTCTCTT   1160
Consensus        gaagaactccatcccagcacaccagttggaactttctctt

PRR95-Q102.seq   AGAAGAACTGACTATGGAAAATTAGAGAACCACGAGAAAA   1200
PRR95-S122.seq   AGAAGAACTGACTATGGAAAATTAGAGAACCACGAGAAAA   1200
Consensus        agaagaactgactatggaaaattagagaaccacgagaaaa

PRR95-Q102.seq   ATGATAGAAGAACACTGAACCATTCAACTTCATCTGCATT   1240
PRR95-S122.seq   ATGATAGAAGAACACTGAACCATTCAACTTCATCTGCATT   1240
Consensus        atgatagaagaacactgaaccattcaacttcatctgcatt

PRR95-Q102.seq   TTACTTGTATAATTGCAGGACAGCGTCCAGCTTAGGAAAT   1280
PRR95-S122.seq   TTACTTGTATAATTGCAGGACAGCGTCCAGCTTAGGAAAT   1280
Consensus        ttacttgtataattgcaggacagcgtccagcttaggaaat

PRR95-Q102.seq   GCTGGTGATGGTCAATTATGCAGCACCTCAGAAACACTAG   1320
PRR95-S122.seq   GCTGGTGATGGTCAATTATGCAGCACCTCAGAAACACTAG   1320
Consensus        gctggtgatggtcaattatgcagcacctcagaaacactag

PRR95-Q102.seq   TGGATGTTGAAAATAGAAATGGAGATTTGGCAGCTCTGTC   1360
PRR95-S122.seq   TGGATGTTGAAAATAGAAATGGAGATTTGGCAGCTCTGTC   1360
Consensus        tggatgttgaaaatagaaatggagatttggcagctctgtc

PRR95-Q102.seq   TGAAGACACGACTGAAACAAATCGTCCTCCTATCAGAGTT   1400
PRR95-S122.seq   TGAAGACACGACTGAAACAAATCGTCCTCCTATCAGAGTT   1400
Consensus        tgaagacacgactgaaacaaatcgtcctcctatcagagtt

PRR95-Q102.seq   GTACCATTTCCTGTCCCTGTTCAATGCTTCACATTTGATG   1440
PRR95-S122.seq   GTACCATTTCCTGTCCCTGTTCAATGCTTCACATTTGATG   1440
Consensus        gtaccatttcctgtccctgttcaatgcttcacatttgatg

PRR95-Q102.seq   GGCAGCCGTTCTGGAATGGTACACCGGTGGCATCTCCATT   1480
PRR95-S122.seq   GGCAGCCGTTCTGGAATGGTACACCGGTGGCATCTCCATT   1480
Consensus        ggcagccgttctggaatggtacaccggtggcatctccatt

PRR95-Q102.seq   CTACCCACAGTCAGCTCCCCCCATTTGGAATAGCAAAACA   1520
PRR95-S122.seq   CTACCCACAGTCAGCTCCCCCCATTTGGAATAGCAAAACA   1520
Consensus        ctacccacagtcagctccccccatttggaatagcaaaaca

PRR95-Q102.seq   CCAACGTGGCAAGAATCAACTCCACAAGCAACTTCACTGC   1560
PRR95-S122.seq   CCAACGTGGCAAGAATCAACTCCACAAGCAACTTCACTGC   1560
Consensus        ccaacgtggcaagaatcaactccacaagcaacttcactgc

PRR95-Q102.seq   CACAAAAATCGCAACAGAAAGAACCAGTTGAAATGGATGC   1600
PRR95-S122.seq   CACAAAAATCGCAACAGAAAGAACCAGTTGAAATGGATGC   1600
Consensus        cacaaaaatcgcaacagaaagaaccagttgaaatggatgc

PRR95-Q102.seq   TAAACCAGTTGAAAATGCAGAGGAACAATTTGTCGTAGGT   1640
PRR95-S122.seq   TAAACCAGTTGAAAATGCAGAGGAACAATTTGTCGTAGGT   1640
Consensus        taaaccagttgaaaatgcagaggaacaatttgtcgtaggt

PRR95-Q102.seq   CCTCCCAGTGCCAGTGGGAAGCAGCTGCATGTTGAAATTC   1680
PRR95-S122.seq   CCTCCCAGTGCCAGTGGGAAGCAGCTGCATGTTGAAATTC   1680
Consensus        cctcccagtgccagtgggaagcagctgcatgttgaaattc

PRR95-Q102.seq   CTAAAGATGATCTACGGCATATTTCTCCTGTGACCGGTGA   1720
PRR95-S122.seq   CTAAAGATGATCTACGGCATATTTCTCCTGTGACCGGTGA   1720
Consensus        ctaaagatgatctacggcatatttctcctgtgaccggtga

PRR95-Q102.seq   AAGTGGAATCAGTACGGTGCTAGACAGCACCAGAAATACT   1760
PRR95-S122.seq   AAGTGGAATCAGTACGGTGCTAGACAGCACCAGAAATACT   1760
Consensus        aagtggaatcagtacggtgctagacagcaccagaaatact

PRR95-Q102.seq   CTGAGCAGCAGTGGCTGTGATAGCACTTCCAACCAGATCA   1800
PRR95-S122.seq   CTGAGCAGCAGTGGCTGTGATAGCACTTCCAACCAGATCA   1800
Consensus        ctgagcagcagtggctgtgatagcacttccaaccagatca

PRR95-Q102.seq   CCGCCCCTACTGAACCATCATCCAATGTATACAGGGGCGT   1840
PRR95-S122.seq   CCGCCCCTACTGAACCATCATCCAATGTATACAGGGGCGT   1840
Consensus        ccgcccctactgaaccatcatccaatgtatacaggggcgt

PRR95-Q102.seq   TCCTGAAACCGCAAGAGCCGAAGGTTTACGACACCTGAGC   1880
PRR95-S122.seq   TCCTGAAACCGCAAGAGCCGAAGGTTTACGACACCTGAGC   1880
Consensus        tcctgaaaccgcaagagccgaaggtttacgacacctgagc

PRR95-Q102.seq   CAGCGTGAGGCTGCACTGAACAAGTTCCGGCTAAAGAGGA   1920
PRR95-S122.seq   CAGCGTGAGGCTGCACTGAACAAGTTCCGGCTAAAGAGGA   1920
Consensus        cagcgtgaggctgcactgaacaagttccggctaaagagga

PRR95-Q102.seq   AGGACAGATGCTTTGAGAAGAAGGTTCGGTACCAGAGCCG   1960
PRR95-S122.seq   AGGACAGATGCTTTGAGAAGAAGGTTCGGTACCAGAGCCG   1960
Consensus        aggacagatgctttgagaagaaggttcggtaccagagccg

PRR95-Q102.seq   GAAATTACTCGCAGAGCAGCGTCCACGGGTCAAGGGCCAG   2000
PRR95-S122.seq   GAAATTACTCGCAGAGCAGCGTCCACGGGTCAAGGGCCAG   2000
Consensus        gaaattactcgcagagcagcgtccacgggtcaagggccag

PRR95-Q102.seq   TTTGTCCGTCAAGATCATAGCATCCAAGGAAGCTAGGGTC   2040
PRR95-S122.seq   TTTGTCCGTCAAGATCATAGCATCCAAGGAAGCTAGGGTC   2040
Consensus        tttgtccgtcaagatcatagcatccaaggaagctagggtc

PRR95-Q102.seq   CAGTTACTGAGCTGGAACTCTTTCCCATTATCTACAAAAA   2080
PRR95-S122.seq   CAGTTACTGAGCTGGAACTCTTTCCCATTATCTACAAAAA   2080
Consensus        cagttactgagctggaactctttcccattatctacaaaaa

PRR95-Q102.seq   AAAGTCACTGTTAGGCACAGGACCTGGCACGTTCTAGACG   2120
PRR95-S122.seq   AAAGTCACTGTTAGGCACAGGACCTGGCACGTTCTAGACG   2120
Consensus        aaagtcactgttaggcacaggacctggcacgttctagacg

PRR95-Q102.seq   GTTTGTCGGTAGACGTTAAAAAATACAGGCATACTTGTGT   2160
PRR95-S122.seq   GTTTGTCGGTAGACGTTAAAAAATACAGGCATACTTGTGT   2160
Consensus        gtttgtcggtagacgttaaaaaatacaggcatacttgtgt

PRR95-Q102.seq   CCTCCAGTAGCACGTGTGCTTTAAGCCTTGTATTATGTAA   2200
PRR95-S122.seq   CCTCCAGTAGCACGTGTGCTTTAAGCCTTGTATTATGTAA   2200
Consensus        cctccagtagcacgtgtgctttaagccttgtattatgtaa

PRR95-Q102.seq   CTAGCCAAGTTGTTTTTGATGTTTCGGATTAGATGTACAC   2240
PRR95-S122.seq   CTAGCCAAGTTGTTTTTGATGTTTCGGATTAGATGTACAC   2240
Consensus        ctagccaagttgtttttgatgtttcggattagatgtacac

PRR95-Q102.seq   TGTGGATGTATGGCGTGAAGGATGTCCACCAAAGTATGCT   2280
PRR95-S122.seq   TGTGGATGTATGGCGTGAAGGATGTCCACCAAAGTATGCT   2280
Consensus        tgtggatgtatggcgtgaaggatgtccaccaaagtatgct

PRR95-Q102.seq   CGTTTCTGTGTAGCTTGTTGCAATGAACATTGTTGGCCTG   2320
PRR95-S122.seq   CGTTTCTGTGTAGCTTGTTGCAATGAACATTGTTGGCCTG   2320
Consensus        cgtttctgtgtagcttgttgcaatgaacattgttggcctg

PRR95-Q102.seq   GTTTTAGTAATGCTCTCTTGCCTTGAG                2347
PRR95-S122.seq   GTTTTAGTAATGCTCTCTTGCCTTGAG                2347
Consensus        gttttagtaatgctctcttgccttgag
```

B

```
PRR73-Q102.seq   ACGGCGAGCGCTTCCCCTCCCCCCTGGTTATCTATCGCAT   40
PRR73-S122.seq   ACGGCGAGCGCTTCCCCTCCCCCCTGGTTATCTATCGCAT   40
Consensus        acggcgagcgcttcccctcccccctggttatctatcgcat

PRR73-Q102.seq   CCTCCGCGGCCCTCCTCCCTCCCACTCCTGTCCTCTCCTC   80
PRR73-S122.seq   CCTCCGCGGCCCTCCTCCCTCCCACTCCTGTCCTCTCCTC   80
Consensus        cctccgcggccctcctccctcccactcctgtcctctcctc

PRR73-Q102.seq   TCCTCTCCCCGCCATTTGGCTCCGAACCCTCACAACAGTG   120
PRR73-S122.seq   TCCTCTCCCCGCCATTTGGCTCCGAACCCTCACAACAGTG   120
Consensus        tcctctccccgccatttggctccgaaccctcacaacagtg

PRR73-Q102.seq   CATCGTCTCTCCTGTCCCCGCCATTTGGTTCGGAACTCTC   160
PRR73-S122.seq   CATCGTCTCTCCTGTCCCCGCCATTTGGTTCGGAACTCTC   160
Consensus        catcgtctctcctgtccccgccatttggttcggaactctc

PRR73-Q102.seq   GCGGCCTCAACTCCTCCTCGTCATCGTAACACCCACGCTT   200
PRR73-S122.seq   GCGGCCTCAACTCCTCCTCGTCATCGTAACACCCACGCTT   200
Consensus        gcggcctcaactcctcctcgtcatcgtaacacccacgctt

PRR73-Q102.seq   AGAGTTCGTGAGGGTCGCCCCCAGGGGATCTCGAGACGTC   240
PRR73-S122.seq   AGAGTTCGTGAGGGTCGCCCCCAGGGGATCTCGAGACGTC   240
Consensus        agagttcgtgagggtcgcccccaggggatctcgagacgtc

PRR73-Q102.seq   CGTTTCCAAGAGTGGACCGGCTCGCAGGCGCTGCTGTTAC   280
PRR73-S122.seq   CGTTTCCAAGAGTGGACCGGCTCGCAGGCGCTGCTGTTAC   280
Consensus        cgtttccaagagtggaccggctcgcaggcgctgctgttac

PRR73-Q102.seq   CTTGGGTGGAGCTCGGACCTGCGGATCAGTTGGATTCTCA   320
PRR73-S122.seq   CTTGGGTGGAGCTCGGACCTGCGGATCAGTTGGATTCTCA   320
Consensus        cttgggtggagctcggacctgcggatcagttggattctca

PRR73-Q102.seq   AGGGAGACGGTGGTTTCTGATTTCCTTTCTCTAAGCATAC   360
PRR73-S122.seq   AGGGAGACGGTGGTTTCTGATTTCCTTTCTCTAAGCATAC   360
Consensus        agggagacggtggtttctgatttcctttctctaagcatac

PRR73-Q102.seq   CAAATCCTTTGTCTCGAGTCGGGAGCAACTGGTTTTGGGC   400
PRR73-S122.seq   CAAATCCTTTGTCTCGAGTCGGGAGCAACTGGTTTTGGGC   400
Consensus        caaatcctttgtctcgagtcgggagcaactggttttgggc

PRR73-Q102.seq   GTCGCGGAGCACATGGCTTAAGAATAATTTCTTCCAGCCC   440
PRR73-S122.seq   GTCGCGGAGCACATGGCTTAAGAATAATTTCTTCCAGCCC   440
Consensus        gtcgcggagcacatggcttaagaataatttcttccagccc

PRR73-Q102.seq   GGCGACAAATTCGCAGTTGGATTCAGCATATGCATTTTTT   480
PRR73-S122.seq   GGCGACAAATTCGCAGTTGGATTCAGCATATGCATTTTTT   480
Consensus        ggcgacaaattcgcagttggattcagcatatgcatttttt

PRR73-Q102.seq   ATGCTGACATAGGATGTGCTCAACCATCCGTGGACTCCCT   520
PRR73-S122.seq   ATGCTGACATAGGATGTGCTCAACCATCCGTGGACTCCCT   520
Consensus        atgctgacataggatgtgctcaaccatccgtggactccct

PRR73-Q102.seq   CTAGTTGCACAAACCACCTCAGTGCTCTCTCTTTCTTCGA   560
PRR73-S122.seq   CTAGTTGCACAAACCACCTCAGTGCTCTCTCTTTCTTCGA   560
Consensus        ctagttgcacaaaccacctcagtgctctctctttcttcga

PRR73-Q102.seq   ATCGGGCTAATAATGGGCAGTGCTTGCCAAGCTGGCACAG   600
PRR73-S122.seq   ATCGGGCTAATAATGGGCAGTGCTTGCCAAGCTGGCACAG   600
Consensus        atcgggctaataatgggcagtgcttgccaagctggcacag

PRR73-Q102.seq   ACGGGCCTTCCCGCAAGGATGTGTTAGGGATAGGGAATGC   640
PRR73-S122.seq   ACGGGCCTTCCCGCAAGGATGTGTTAGGGATAGGGAATGC   640
Consensus        acgggccttcccgcaaggatgtgttagggatagggaatgc

PRR73-Q102.seq   CGCCTTAGAGAATGGCCACCATCAGGCTGAAGCTGACGCA   680
PRR73-S122.seq   CGCCTTAGAGAATGGCCACCATCAGGCTGAAGCTGACGCA   680
Consensus        cgccttagagaatggccaccatcaggctgaagctgacgca

PRR73-Q102.seq   GATGAATGGAGGGAAAAGGAAGAGGACTTGGCCAACAACG   720
PRR73-S122.seq   GATGAATGGAGGGAAAAGGAAGAGGACTTGGCCAACAACG   720
Consensus        gatgaatggagggaaaaggaagaggacttggccaacaacg

PRR73-Q102.seq   GGCACAGTGCGCCACCGCCAGGCATGCAGCAGGTGGATGA   760
PRR73-S122.seq   GGCACAGTGCGCCACCGCCAGGCATGCAGCAGGTGGATGA   760
Consensus        ggcacagtgcgccaccgccaggcatgcagcaggtggatga

PRR73-Q102.seq   GCATAAGGAGGAACAAAGACAAAGCATTCACTGGGAGAGG   800
PRR73-S122.seq   GCATAAGGAGGAACAAAGACAAAGCATTCACTGGGAGAGG   800
Consensus        gcataaggaggaacaaagacaaagcattcactgggagagg

PRR73-Q102.seq   TTCCTACCTGTGAAGACACTGAGAGTCTTGCTGGTGGAGA   840
PRR73-S122.seq   TTCCTACCTGTGAAGACACTGAGAGTCTTGCTGGTGGAGA   840
Consensus        ttcctacctgtgaagacactgagagtcttgctggtggaga

PRR73-Q102.seq   ATGATGACTCTACTCGTCAGGTGGTCAGTGCCCTGCTCCG   880
PRR73-S122.seq   ATGATGACTCTACTCGTCAGGTGGTCAGTGCCCTGCTCCG   880
Consensus        atgatgactctactcgtcaggtggtcagtgccctgctccg

PRR73-Q102.seq   TAAGTGCTGCTATGAAGTTATTCCTGCTGAAAATGGTTTG   920
PRR73-S122.seq   TAAGTGCTGCTATGAAGTTATTCCTGCTGAAAATGGTTTG   920
Consensus        taagtgctgctatgaagttattcctgctgaaaatggtttg

PRR73-Q102.seq   CATGCATGGCGATATCTTGAAGATCTGCAGAACAACATCG   960
PRR73-S122.seq   CATGCATGGCGATATCTTGAAGATCTGCAGAACAACATCG   960
Consensus        catgcatggcgatatcttgaagatctgcagaacaacatcg

PRR73-Q102.seq   ACCTTGTATTGACTGAGGTTTTCATGCCTTGTCTATCTGG   1000
PRR73-S122.seq   ACCTTGTATTGACTGAGGTTTTCATGCCTTGTCTATCTGG   1000
Consensus        accttgtattgactgaggttttcatgccttgtctatctgg

PRR73-Q102.seq   TATCGGTCTGCTTAGCAAAATCACAAGTCACAAAATTTGC   1040
PRR73-S122.seq   TATCGGTCTGCTTAGCAAAATCACAAGTCACAAAATTTGC   1040
Consensus        tatcggtctgcttagcaaaatcacaagtcacaaaatttgc

PRR73-Q102.seq   AAAGACATTCCTGTGATTATGATGTCTACGAATGATTCTA   1080
PRR73-S122.seq   AAAGACATTCCTGTGATTATGATGTCTACGAATGATTCTA   1080
Consensus        aaagacattcctgtgattatgatgtctacgaatgattcta

PRR73-Q102.seq   TGAGTATGGTGTTTAAGTGTTTGTCGAAGGGAGCAGTTGA   1120
PRR73-S122.seq   TGAGTATGGTGTTTAAGTGTTTGTCGAAGGGAGCAGTTGA   1120
Consensus        tgagtatggtgtttaagtgtttgtcgaagggagcagttga

PRR73-Q102.seq   TTTCTTGGTAAAACCACTACGTAAGAATGAGCTTAAGAAC   1160
PRR73-S122.seq   TTTCTTGGTAAAACCACTACGTAAGAATGAGCTTAAGAAC   1160
Consensus        tttcttggtaaaaccactacgtaagaatgagcttaagaac

PRR73-Q102.seq   CTTTGGCAGCATGTTTGGAGGCGATGCCACAGTTCCAGTG   1200
PRR73-S122.seq   CTTTGGCAGCATGTTTGGAGGCGATGCCACAGTTCCAGTG   1200
Consensus        ctttggcagcatgtttggaggcgatgccacagttccagtg

PRR73-Q102.seq   GAAGTGAAAGTGGCATCCAGACACAGAAGTGTGCCAAACT   1240
PRR73-S122.seq   GAAGTGAAAGTGGCATCCAGACACAGAAGTGTGCCAAACT   1240
Consensus        gaagtgaaagtggcatccagacacagaagtgtgccaaact

PRR73-Q102.seq   AAATACTGGCGACGAGTATGAGAACGGCAGTGACAGCAAT   1280
PRR73-S122.seq   AAATACTGGCGACGAGTATGAGAACGGCAGTGACAGCAAT   1280
Consensus        aaatactggcgacgagtatgagaacggcagtgacagcaat

PRR73-Q102.seq   CATGATGATGAAGAAAATGATGACGGCGACGATGACGACT   1320
PRR73-S122.seq   CATGATGATGAAGAAAATGATGACGGCGACGATGACGACT   1320
Consensus        catgatgatgaagaaaatgatgacggcgacgatgacgact

PRR73-Q102.seq   TCAGTGTTGGACTCAATGCTAGGGATGGAAGTGACAATGG   1360
PRR73-S122.seq   TCAGTGTTGGACTCAATGCTAGGGATGGAAGTGACAATGG   1360
Consensus        tcagtgttggactcaatgctagggatggaagtgacaatgg

PRR73-Q102.seq   CAGTGGTACTCAAAGCTCATGGACAAAGCGTGCTGTGGAG   1400
PRR73-S122.seq   CAGTGGTACTCAAAGCTCATGGACAAAGCGTGCTGTGGAG   1400
Consensus        cagtggtactcaaagctcatggacaaagcgtgctgtggag

PRR73-Q102.seq   ATTGACAGCCCACAACCTATATCTCCCGATCAACTAGTTG   1440
PRR73-S122.seq   ATTGACAGCCCACAACCTATATCTCCCGATCAACTAGTTG   1440
Consensus        attgacagcccacaacctatatctcccgatcaactagttg

PRR73-Q102.seq   ATCCACCTGATAGTACATGTGCACAAGTAATTCACCCTAG   1480
PRR73-S122.seq   ATCCACCTGATAGTACATGTGCACAAGTAATTCACCCTAG   1480
Consensus        atccacctgatagtacatgtgcacaagtaattcaccctag

PRR73-Q102.seq   ATCAGAGATATGCAGTAACAAGTGGTTACCGACAGCAAAC   1520
PRR73-S122.seq   ATCAGAGATATGCAGTAACAAGTGGTTACCGACAGCAAAC   1520
Consensus        atcagagatatgcagtaacaagtggttaccgacagcaaac

PRR73-Q102.seq   AAAAGGAATGTCAAGAAACAGAAGGAGAATAAAGATGAAT   1560
PRR73-S122.seq   AAAAGGAATGTCAAGAAACAGAAGGAGAATAAAGATGAAT   1560
Consensus        aaaaggaatgtcaagaaacagaaggagaataaagatgaat

PRR73-Q102.seq   CTATGGGAAGATACTTAGGAATAGGTGCTCCTAGGAACTC   1600
PRR73-S122.seq   CTATGGGAAGATACTTAGGAATAGGTGCTCCTAGGAACTC   1600
Consensus        ctatgggaagatacttaggaataggtgctcctaggaactc

PRR73-Q102.seq   AAGTGCAGAATATCAATCATCTCTCAATGATGTATCTGTT   1640
PRR73-S122.seq   AAGTGCAGAATATCAATCATCTCTCAATGATGTATCTGTT   1640
Consensus        aagtgcagaatatcaatcatctctcaatgatgtatctgtt

PRR73-Q102.seq   AATCCAATAGAAAAAGGACATGAGAATCACATGTCCAAAT   1680
PRR73-S122.seq   AATCCAATAGAAAAAGGACATGAGAATCACATGTCCAAAT   1680
Consensus        aatccaatagaaaaaggacatgagaatcacatgtccaaat

PRR73-Q102.seq   GCAAATCTAAAAAGGAAACAATGGCAGAAGATGATTGTAC   1720
PRR73-S122.seq   GCAAATCTAAAAAGGAAACAATGGCAGAAGATGATTGTAC   1720
Consensus        gcaaatctaaaaaggaaacaatggcagaagatgattgtac

PRR73-Q102.seq   AAACATGCCTAGTGCAACAAATGCTGAAACTGCTGATTTG   1760
PRR73-S122.seq   AAACATGCCTAGTGCAACAAATGCTGAAACTGCTGATTTG   1760
Consensus        aaacatgcctagtgcaacaaatgctgaaactgctgatttg

PRR73-Q102.seq   ATTAGCTCAATAGCCAGAAACACAGAAGGCCAACAAGCAG   1800
PRR73-S122.seq   ATTAGCTCAATAGCCAGAAACACAGAAGGCCAACAAGCAG   1800
Consensus        attagctcaatagccagaaacacagaaggccaacaagcag

PRR73-Q102.seq   TACAAGCCGTTGACGCACCAGATGGCCCTTCCAAAATGGC   1840
PRR73-S122.seq   TACAAGCCGTTGACGCACCAGATGGCCCTTCCAAAATGGC   1840
Consensus        tacaagccgttgacgcaccagatggcccttccaaaatggc

PRR73-Q102.seq   TAATGGAAATGATAAGAATCATGATTCTCATATCGAAGTG   1880
PRR73-S122.seq   TAATGGAAATGATAAGAATCATGATTCTCATATCGAAGTG   1880
Consensus        taatggaaatgataagaatcatgattctcatatcgaagtg

PRR73-Q102.seq   ACACCCCATGAGTTGGGTTTGAAGAGATCGAGAACAAATG   1920
PRR73-S122.seq   ACACCCCATGAGTTGGGTTTGAAGAGATCGAGAACAAATG   1920
Consensus        acaccccatgagttgggtttgaagagatcgagaacaaatg

PRR73-Q102.seq   GAGCTACAGCGGAAATCCATGATGAGCGAAATATTCTGAA   1960
PRR73-S122.seq   GAGCTACAGCGGAAATCCATGATGAGCGAAATATTCTGAA   1960
Consensus        gagctacagcggaaatccatgatgagcgaaatattctgaa

PRR73-Q102.seq   AAGATCAGATCAGTCAGCCTTCACCAGGTACCATACATCT   2000
PRR73-S122.seq   AAGATCAGATCAGTCAGCCTTCACCAGGTACCATACATCT   2000
Consensus        aagatcagatcagtcagccttcaccaggtaccatacatct

PRR73-Q102.seq   GTGGCTTCCAATCAAGGTGGAGCAAGATATGGGGAAAGCT   2040
PRR73-S122.seq   GTGGCTTCCAATCAAGGTGGAGCAAGATATGGGGAAAGCT   2040
Consensus        gtggcttccaatcaaggtggagcaagatatggggaaagct

PRR73-Q102.seq   CTTCACCACAAGATAACAGTTCTGAGGCCATGAAAACGGA   2080
PRR73-S122.seq   CTTCACCACAAGATAACAGTTCTGAGGCCATGAAAACGGA   2080
Consensus        cttcaccacaagataacagttctgaggccatgaaaacgga

PRR73-Q102.seq   CTCTACATGCAAGATGAAGTCAAATTCAGATGCTGCTCCA   2120
PRR73-S122.seq   CTCTACATGCAAGATGAAGTCAAATTCAGATGCTGCTCCA   2120
Consensus        ctctacatgcaagatgaagtcaaattcagatgctgctcca

PRR73-Q102.seq   ATAAAGCAGGGCTCCAATGGCAGTAGCAATAACGATGTGG   2160
PRR73-S122.seq   ATAAAGCAGGGCTCCAATGGCAGTAGCAATAACGATGTGG   2160
Consensus        ataaagcagggctccaatggcagtagcaataacgatgtgg

PRR73-Q102.seq   GATCCAGTACAAAGAATGTTGCTGCAAGGCCTTCGGGTGA   2200
PRR73-S122.seq   GATCCAGTACAAAGAATGTTGCTGCAAGGCCTTCGGGTGA   2200
Consensus        gatccagtacaaagaatgttgctgcaaggccttcgggtga

PRR73-Q102.seq   CAGGGAGAGAGTAGCGTCACCATTAGCCATCAAATCTACC   2240
PRR73-S122.seq   CAGGGAGAGAGTAGCGTCACCATTAGCCATCAAATCTACC   2240
Consensus        cagggagagagtagcgtcaccattagccatcaaatctacc

PRR73-Q102.seq   CAGCATGCCTCAGCATTTCATACTATACAGAATCAAACGT   2280
PRR73-S122.seq   CAGCATGCCTCAGCATTTCATACTATACAGAATCAAACGT   2280
Consensus        cagcatgcctcagcatttcatactatacagaatcaaacgt

PRR73-Q102.seq   CACCAGCTAATCTGATTGGGGAAGACAAAGCTGATGAAGG   2320
PRR73-S122.seq   CACCAGCTAATCTGATTGGGGAAGACAAAGCTGATGAAGG   2320
Consensus        caccagctaatctgattggggaagacaaagctgatgaagg

PRR73-Q102.seq   AATTTCCAATACAGTGAAAATGAGCCACCCAACAGAGGTT   2360
PRR73-S122.seq   AATTTCCAATACAGTGAAAATGAGCCACCCAACAGAGGTT   2360
Consensus        aatttccaatacagtgaaaatgagccacccaacagaggtt

PRR73-Q102.seq   CCACAAGGCTGCGTCCAGCATCATCATCATGTGCATTATT   2400
PRR73-S122.seq   CCACAAGGCTGCGTCCAGCATCATCATCATGTGCATTATT   2400
Consensus        ccacaaggctgcgtccagcatcatcatcatgtgcattatt
```

```
PRR73-Q102.seq    ACCTCCATGTTATGACACAGAAACAGCCATCAACAGACCG    2440
PRR73-S122.seq    ACCTCCATGTTATGACACAGAAACAGCCATCAACAGACCG    2440
Consensus         acctccatgttatgacacagaaacagccatcaacagaccg

PRR73-Q102.seq    TGGATCATCAGATGTTCACTGTGGTTCGTCAAATGTGTTT    2480
PRR73-S122.seq    TGGATCATCAGATGTTCACTGTGGTTCGTCAAATGTGTTT    2480
Consensus         tggatcatcagatgttcactgtggttcgtcaaatgtgttt

PRR73-Q102.seq    GATCCTCCTGTTGAAGGACATGCTGCAAACTACAGTGTGA    2520
PRR73-S122.seq    GATCCTCCTGTTGAAGGACATGCTGCAAACTACAGTGTGA    2520
Consensus         gatcctcctgttgaaggacatgctgcaaactacagtgtga

PRR73-Q102.seq    ATGGGGGTGTCTCAGTTGGTCATAATGGGTGCAATGGGCA    2560
PRR73-S122.seq    ATGGGGGTGTCTCAGTTGGTCATAATGGGTGCAATGGGCA    2560
Consensus         atgggggtgtctcagttggtcataatgggtgcaatgggca

PRR73-Q102.seq    GAATGGAAGTAGCGCTGTCCCCAATATTGCAAGACCAAAC    2600
PRR73-S122.seq    GAATGGAAGTAGCGCTGTCCCCAATATTGCAAGACCAAAC    2600
Consensus         gaatggaagtagcgctgtccccaatattgcaagaccaaac

PRR73-Q102.seq    ATAGAGAGTATTAATGGTACCATGAGCCAAAATATTGCCG    2640
PRR73-S122.seq    ATAGAGAGTATTAATGGTACCATGAGCCAAAATATTGCCG    2640
Consensus         atagagagtattaatggtaccatgagccaaaatattgccg

PRR73-Q102.seq    GAGGTGGCATTGTAAGTGGGAGTGGGAGTGGCAATGACAT    2680
PRR73-S122.seq    GAGGTGGCATTGTAAGTGGGAGTGGGAGTGGCAATGACAT    2680
Consensus         gaggtggcattgtaagtgggagtgggagtggcaatgacat

PRR73-Q102.seq    GTATCAGAATCGGTTCCTGCAACGAGAAGCTGCATTGAAC    2720
PRR73-S122.seq    GTATCAGAATCGGTTCCTGCAACGAGAAGCTGCATTGAAC    2720
Consensus         gtatcagaatcggttcctgcaacgagaagctgcattgaac

PRR73-Q102.seq    AAATTCAGACTGAAGCGGAAAGATCGGAACTTTGGTAAAA    2760
PRR73-S122.seq    AAATTCAGACTGAAGCGGAAAGATCGGAACTTTGGTAAAA    2760
Consensus         aaattcagactgaagcggaaagatcggaactttggtaaaa

PRR73-Q102.seq    AGGTTCGCTACCAAAGCAGGAAGAGGCTTGCTGAGCAGCG    2800
PRR73-S122.seq    AGGTTCGCTACCAAAGCAGGAAGAGGCTTGCTGAGCAGCG    2800
Consensus         aggttcgctaccaaagcaggaagaggcttgctgagcagcg

PRR73-Q102.seq    GCCACGGGTCCGAGGACAGTTTGTGCGACAATCTGAGCAA    2840
PRR73-S122.seq    GCCACGGGTCCGAGGACAGTTTGTGCGACAATCTGAGCAA    2840
Consensus         gccacgggtccgaggacagtttgtgcgacaatctgagcaa

PRR73-Q102.seq    GAAGATCAAACAGCGCAAGGTTCAGAAAGATGACACGTAA    2880
PRR73-S122.seq    GAAGATCAAACAGCGCAAGGTTCAGAAAGATGACACGTAA    2880
Consensus         gaagatcaaacagcgcaaggttcagaaagatgacacgtaa

PRR73-Q102.seq    TTTCTAATACAACGTATCATCTTCTTCGGCAGATCTGAGA    2920
PRR73-S122.seq    TTTCTAATACAACGTATCATCTTCTTCGGCAGATCTGAGA    2920
Consensus         tttctaatacaacgtatcatcttcttcggcagatctgaga

PRR73-Q102.seq    ATTAGGCGGTTCTTCCTATTGCGCATCTTCGTCGACAGAT    2960
PRR73-S122.seq    ATTAGGCGGTTCTTCCTATTGCGCATCTTCGTCGACAGAT    2960
Consensus         attaggcggttcttcctattgcgcatcttcgtcgacagat

PRR73-Q102.seq    CTGAGAATTAAGTGGTCCTTCCCATTGCTGATAGTAGCCC    3000
PRR73-S122.seq    CTGAGAATTAAGTGGTCCTTCCCATTGCTGATAGTAGCCC    3000
Consensus         ctgagaattaagtggtccttcccattgctgatagtagccc

PRR73-Q102.seq    ACTGATTCCCAACGTTAGAGTACACAATAAATAAGACTCT    3040
PRR73-S122.seq    ACTGATTCCCAACGTTAGAGTACACAATAAATAAGACTCT    3040
Consensus         actgattcccaacgttagagtacacaataaataagactct

PRR73-Q102.seq    AGTTGTCAATTTCTGTGGTTGACTGCAGATGATCACCCTA    3080
PRR73-S122.seq    AGTTGTCAATTTCTGTGGTTGACTGCAGATGATCACCCTA    3080
Consensus         agttgtcaatttctgtggttgactgcagatgatcacccta

PRR73-Q102.seq    GCGCTATGGAGTCAACAATGTAATATTTATATACTATGTT    3120
PRR73-S122.seq    GCGCTATGGAGTCAACAATGTAATATTTATATACTATGTT    3120
Consensus         gcgctatggagtcaacaatgtaatatttatatactatgtt

PRR73-Q102.seq    CGTGCCTTGGCGCCTTGGCGGTTTGCAATCTTCAGTTTCC    3160
PRR73-S122.seq    CGTGCCTTGGCGCCTTGGCGGTTTGCAATCTTCAGTTTCC    3160
Consensus         cgtgccttggcgccttggcggtttgcaatcttcagtttcc

PRR73-Q102.seq    TTTCTGAGAGATTCTTGGCCAAGTGCCTTGAGGAGGAGCA    3200
PRR73-S122.seq    TTTCTGAGAGATTCTTGGCCAAGTGCCTTGAGGAGGAGCA    3200
Consensus         tttctgagagattcttggccaagtgccttgaggaggagca

PRR73-Q102.seq    AACTAGCAGCGTCGCCATTGCTATATTAATAGCTGGAATC    3240
PRR73-S122.seq    AACTAGCAGCGTCGCCATTGCTATATTAATAGCTGGAATC    3240
Consensus         aactagcagcgtcgccattgctatattaatagctggaatc

PRR73-Q102.seq    ATGTAGTAGTAGTTCATCCATTCATTCGAAGATCGTGTTC    3280
PRR73-S122.seq    ATGTAGTAGTAGTTCATCCATTCATTCGAAGATCGTGTTC    3280
Consensus         atgtagtagtagttcatccattcattcgaagatcgtgttc

PRR73-Q102.seq    CAAATCAACACAAGTAGGGGTTTGCTTCTCCTCTTTCCAA    3320
PRR73-S122.seq    CAAATCAACACAAGTAGGGGTTTGCTTCTCCTCTTTCCAA    3320
Consensus         caaatcaacacaagtaggggtttgcttctcctctttccaa

PRR73-Q102.seq    GAAAAGTGGTTGTAGCGTGAGATGCAAATAGGTCTCTTGT    3360
PRR73-S122.seq    GAAAAGTGGTTGTAGCGTGAGATGCAAATAGGTCTCTTGT    3360
Consensus         gaaaagtggttgtagcgtgagatgcaaataggtctcttgt

PRR73-Q102.seq    GCTAGATCAAGCTCAATAAACTTTAGGCCGTGTTAGGCAG    3400
PRR73-S122.seq    GCTAGATCAAGCTCAATAAACTTTAGGCCGTGTTAGGCAG    3400
Consensus         gctagatcaagctcaataaactttaggccgtgttaggcag

PRR73-Q102.seq    CTGATCTACTTCACCAATGTGGCAAGGCATTCGTCGTATT    3440
PRR73-S122.seq    CTGATCTACTTCACCAATGTGGCAAGGCATTCGTCGTATT    3440
Consensus         ctgatctacttcaccaatgtggcaaggcattcgtcgtatt

PRR73-Q102.seq    CTGTTGACAGAAACAAGGTGTTCACTGCACGGGCATGCCG    3480
PRR73-S122.seq    CTGTTGACAGAAACAAGGTGTTCACTGCACGGGCATGCCG    3480
Consensus         ctgttgacagaaacaaggtgttcactgcacgggcatgccg

PRR73-Q102.seq    CACGACTCCTCGCAGATCAAAAGAGGGAACACGGCATGCT    3520
PRR73-S122.seq    CACGACTCCTCGCAGATCAAAAGAGGGAACACGGCATGCT    3520
Consensus         cacgactcctcgcagatcaaaagagggaacacggcatgct

PRR73-Q102.seq    TTTACCAAAATTATTTT                           3537
PRR73-S122.seq    TTTACCAAAATTATTTT                           3537
Consensus         tttaccaaaattatttt
```

C

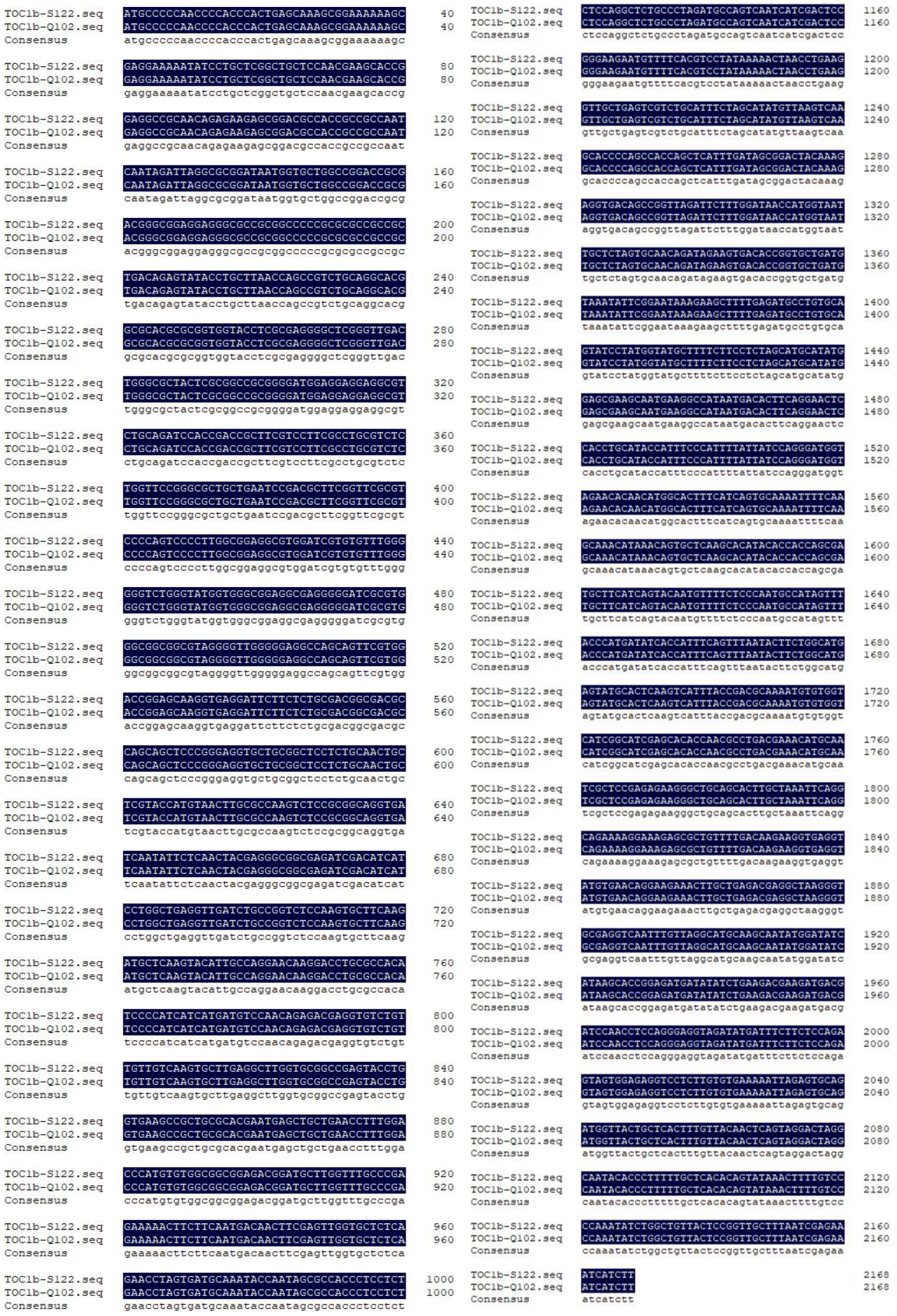

FigureS13: Objective gene sequence alignment results. (A) Sequence alignment of coding region of ZmPRR95 gene.(B) Sequence alignment of coding region of ZmPRR73 gene. (C) Sequence alignment of coding region of ZmTOC1b gene.

A

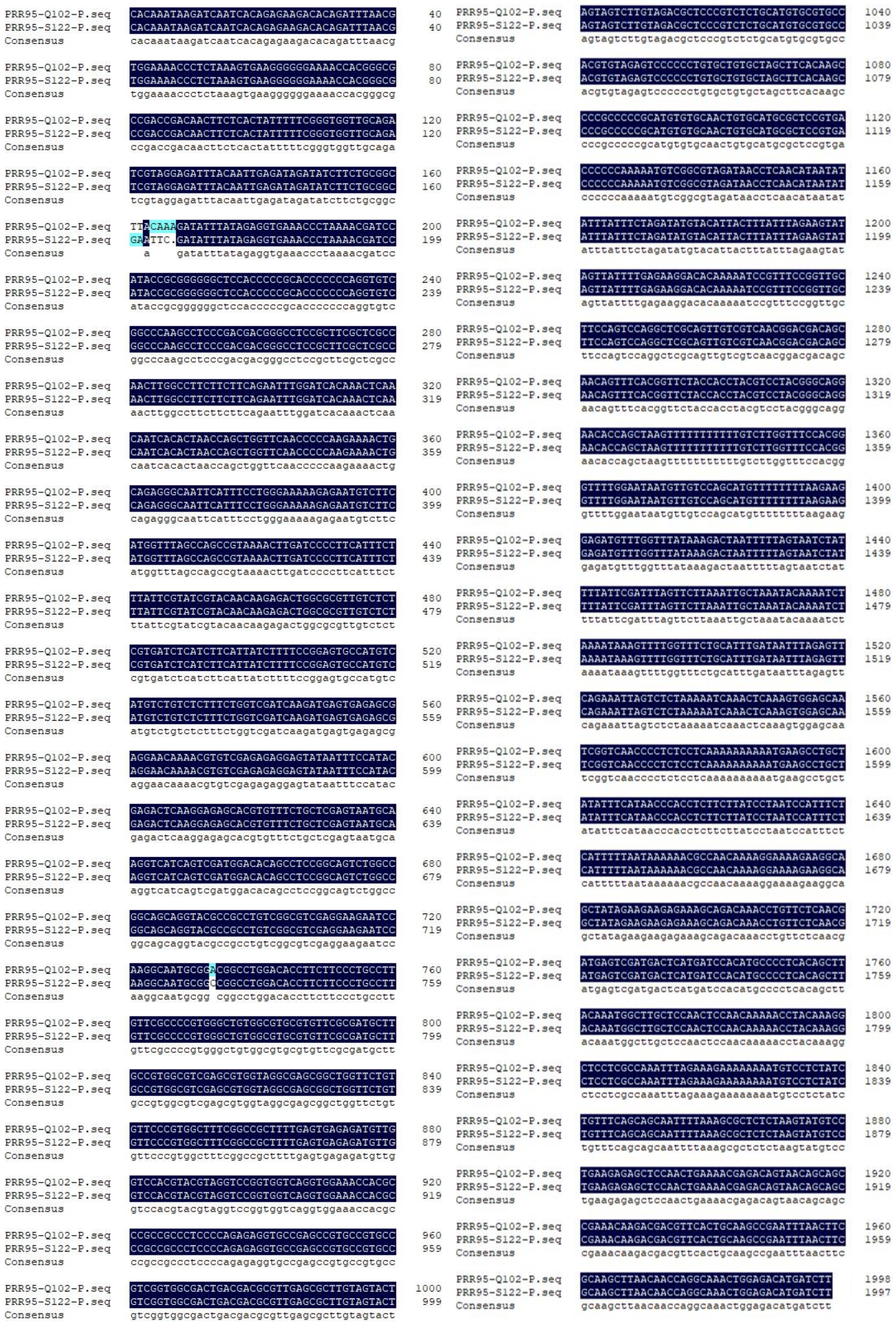

B

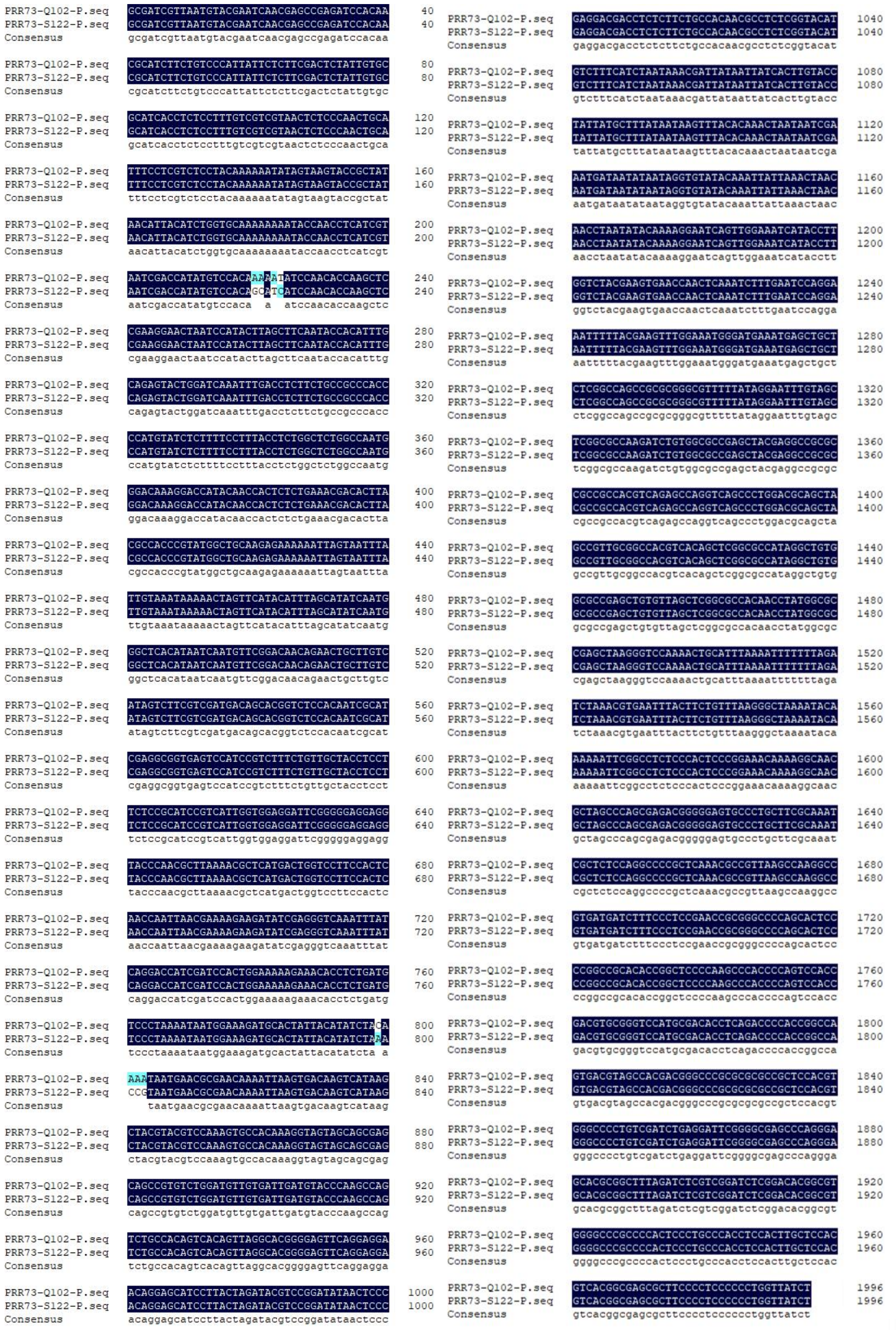

B

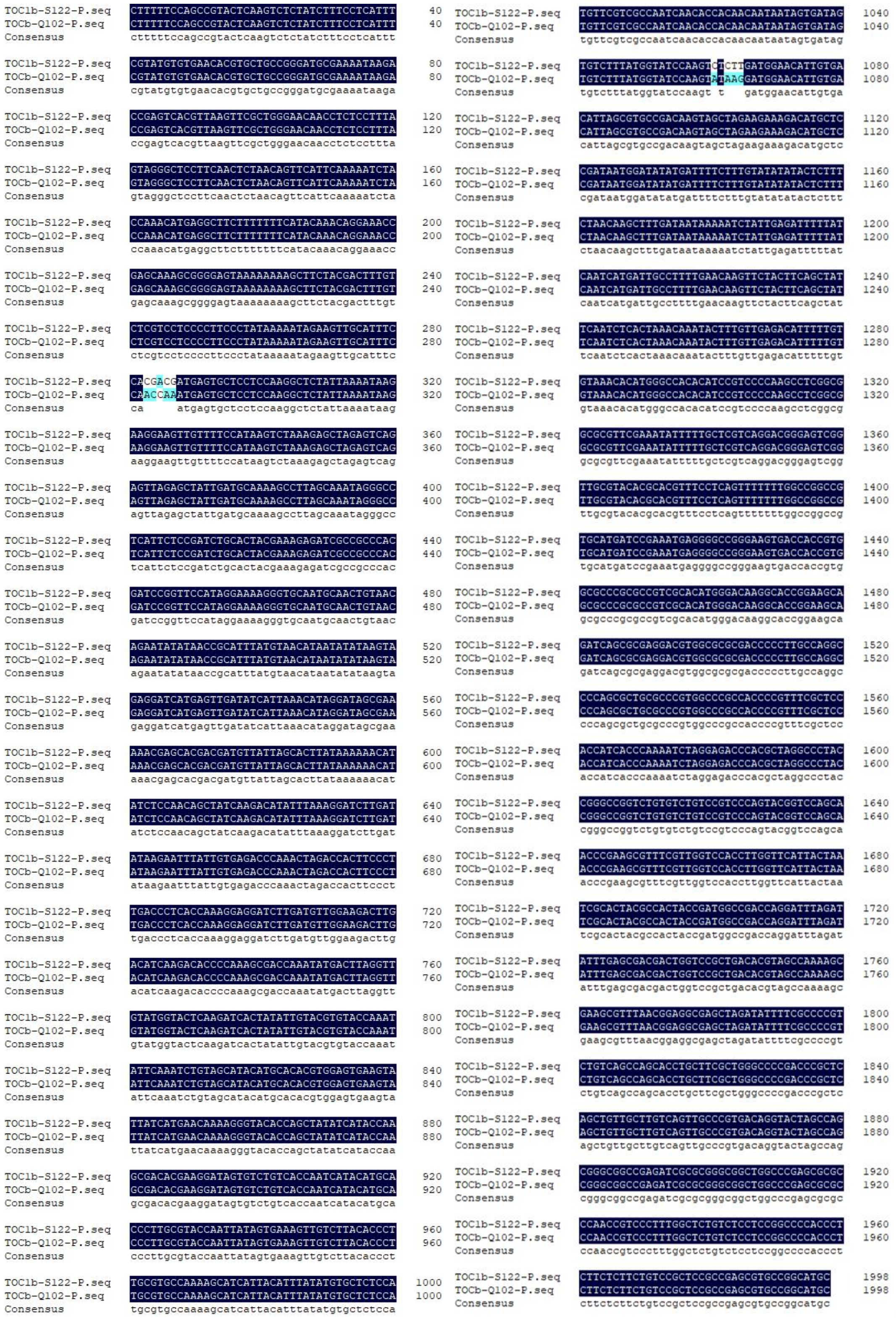

FigureS14: Sequence alignment results of object gene promoter regions. (A) ZmPRR95 promoter region sequence alignment results.(B) ZmPRR73 promoter region sequence alignment results. (C) ZmTOC1 promoter region sequence alignment results.
